# Comprehensive Evaluation of Agromorphological Traits, Yield Potential, and Essential Oil Profiles in 160 Coriander (*Coriandrum sativum* L.) Genotypes

**DOI:** 10.3390/biology15191787

**Published:** 2026-10-08

**Authors:** Eray Meric, Gulsum Yaldiz, Mahmut Camlica

**Affiliations:** 1 Institute of Graduate Studies, Bolu Abant İzzet Baysal University, 14030 Bolu, Türkiye; eray-meric@hotmail.com; 2 Faculty of Agriculture, Department of Field Crops, Bolu Abant İzzet Baysal University, 14030 Bolu, Türkiye; mcamlica25@gmail.com

**Keywords:** plant breeding, quality traits, cilantro, multivariate analysis

## Abstract

Coriander has become increasingly significant from both economic and nutritional perspectives owing to the biologically active compounds obtained from its powdered fruits, dried extracts, herbal infusions, tinctures, and decoctions. To identify high-quality coriander (*Coriandrum sativum*) genotypes, 160 genetic materials were grown under the same environmental conditions and comprehensively analyzed in terms of numerous morphological, agronomic, essential oil, and essential oil component characteristics. The experiment was conducted during the spring-summer season of 2024 in Bolu province located in a transitional climate zone in western Türkiye. Significant phenotypic variation was observed among the genotypes, with fruit yields ranging from 0.05 to 4.98 g/plant. The GN-37, GN-103, and GN-33 genotypes showed the highest fruit yield values. Essential oil content varied between 0.02% and 2.25%, with the GN-154 and GN-88 genotypes exhibiting the highest oil yields. The most important essential oil components of coriander genotypes and cultivars were linalool, β-pinene, γ-terpinene, camphor, trans-geraniol, trans-geranyl acetate, 13-tetradecenal and *myristic* acid. Among these, linalool was detected in all genotypes, ranging from 0.52% to 48.90%, and the GN-114 and GN-73 genotypes revealed the highest linalool content. These findings demonstrate that the investigated coriander genotypes exhibit superior yield and quality attributes, highlighting their considerable potential for economically viable cultivation under appropriate growing conditions. The cluster and principal analyses showed important results among the coriander genotypes for the specific traits examined such as camphor, trans-geraniol and biological yield. The results obtained are expected to form the basis for identifying suitable parental materials and developing new varieties for future coriander breeding programs, particularly through the selection of genotypes with high yield and desired quality characteristics. This comprehensive field assessment contributes not only to determining phenotypic differences among genotypes but also to identifying valuable genetic resources that can be used for coriander improvement. In this respect, the study makes a significant contribution to the evaluation of genetic resources in coriander and, in particular, to identifying superior genotypes usable for breeding in terms of yield, essential oil, and essential oil components.

## Simple Summary

Genetic variability is a fundamental prerequisite for plant breeding, as it provides the necessary variation for genetic improvement and the effective utilization of germplasm in breeding programs. Therefore, plant breeders benefit significantly from seed gene banks, which systematically collect and preserve germplasm under optimal conditions to support international scientific research. In this study, coriander genotypes were obtained from the United States Department of Agriculture (USDA) and the Canadian gene bank, along with local genotypes, to characterize and evaluate broad-based genetic material. A total of 160 genetic materials were grown under the same environmental conditions and comprehensively analyzed for numerous morphological, agronomic, essential oil, and essential oil component characteristics.

## 1. Introduction

*Coriandrum sativum* (coriander) is a multifunctional medicinal and aromatic plant, the economic value of which is largely controlled by its seed yield as well as the qualitative and quantitative composition of its essential oil and fixed oil constituents. The coriander plant originates from the Mediterranean basin and is now cultivated across a wide global geography and used in many fields, from the food industry to pharmaceutical applications. Coriander has reported pharmacological activities, including anticonvulsant [1], antimutagenic [2], antilipidemic, and antispasmodic [3] properties. In addition, the puree and paste obtained by briefly blanching coriander leaves and fruits in water are used as starting materials in the creation of various products [4], and these products are widely used in the fast-food industry today. Coriander contains chemical components that can bind toxic metals and facilitate their removal from cells, and therefore plays a role as a natural detoxification agent [5]. The essential oil, the main biological compound, possesses antimicrobial [6], antibacterial [7], antioxidant [8], and antidiabetic [9] properties. One study noted that coriander essential oil was evaluated for its anti-wrinkle cosmetic potential activity in UV-induced photoaging mice, and it was found that coriander essential oil showed the highest collagenase, elastase, hyaluronidase, and tyrosinase inhibitory activity [10]. The bioactive constituents of coriander essential oil and its naturally derived products have attracted growing scientific interest in recent years due to their promising potential for therapeutic applications. Therefore, the identification, selection, and incorporation of genotypes with high essential oil content into breeding programs are of particular importance for the development of superior cultivars. In the study, the wide differences within the germplasm collection are expected to provide a valuable resource for identifying genotypes with high essential oil content.

The limited genetic diversity in populations and the fact that observed phenotypic differences result from the combined effects of genetic and environmental factors limit breeding opportunities for developing high-yielding varieties [11]. Genetic diversity is important because it enables the selection of heritable traits and defines the range of heritable traits within a crop, making it a requirement for any breeding program [12]. A critical assessment of the nature and magnitude of variability in genetic material is a prerequisite for any productive breeding program and offers the opportunity to identify superior genotypes with desired yield and quality characteristics, thus enabling the development of a high-yielding variety [13]. Although coriander production makes a substantial contribution to the country’s economy, relatively limited attention has been devoted to its genetic improvement and breeding. Coriander cultivars available in Türkiye have generally been developed from a limited number of local genotypes. Therefore, in our long-standing coriander studies, our strategy is to utilize a broad genetic resource base by including different gene banks on a genotype basis and to evaluate existing local genotypes and cultivars together in terms of yield and quality. The identification and selection of superior genotypes are essential for breeders to facilitate the genetic improvement and further development of the crop. Furthermore, the advancement of plant breeding programs depends on the comprehensive characterization and evaluation of available genetic resources.

Gene banks are of great importance in developing these high-yielding varieties, as they possess a wide range of initial genetic material and encompass the entire range of variations found in nature, forming the basis for selection [14]. Therefore, in our study, coriander genotypes were supplied from the United States Department of Agriculture (USDA) and Canada gene banks along with local genotypes to characterize and evaluate the broad-based genetic material.

Previous studies have reported that coriander genotypes showed important phenotypic variations in plant height, branch number, number of umbels per plant and fruit yield among the domestic collections and breeding materials [15,16]. Moreover, it was reported that seed number per umbel and umbellet, node number and dry weight values were associated with seed yield, and these traits may be relevant to yield-related evaluation [17]. The yield and components of coriander essential oil vary considerably depending on plant age, origin, environmental factors, and growth stage [18,19]. Ecological and environmental conditions, cultural practices applied during crop cultivation, harvesting methods, and genotypes significantly affect the essential oil content and composition in leaves and seeds. Numerous researchers in various parts of the world have studied the degree of variation in essential oil content and composition of coriander genotypes [20]. This variability can lead to the emergence of different chemical variants or coriander chemotypes [21].

Genetic manipulation in coriander through plant breeding has been limited to the selection of different genotypes based on morphological characters and seed yield, as hybridization is laborious because of the small size of the flowers. Besides genetic diversity, knowledge of the scope and nature of the genetic architecture of key yield-related traits, and the correlations among traits is essential to improve the efficiency of breeding programs [22]. Although some studies similar to the present study have been conducted, including Chandrakala et al. [18] with 50 coriander genotypes, Mortazavian et al. [17] with 202 coriander genotypes, Sharangi et al. [15] with 15 coriander genotypes, and Başol et al. [23] with 119 coriander genotypes, the literature remains insufficient to guide comprehensive studies on sustainable coriander breeding.

Our studies with coriander began in 2014 with local genotypes and continued in 2019 with genotypes of different origins obtained from the USDA. This study included Canadian genotypes in addition to the existing genotypes, and for the first time, the essential oil values and components of all genotypes were determined, along with their UPOV, morphological, and yield characteristics.

This study targeted 160 coriander genotypes, consisting local genotypes and cultivars, as well as genotypes from the USDA and Canadian gene banks. This study targeted 160 coriander genotypes, consisting local genotypes and cultivars, as well as genotypes from the USDA and Canadian gene banks. Our objectives were: (1) to determine the adaptability of broad-based germplasm obtained from very different geographical conditions under the same ecological conditions; (2) to systematically analyze the variation in the phenological, agronomic, and chemical characteristics of genotypes grown in the field under the same ecological conditions; (3) to provide robust results for exploring the breeding potential of the genotypes. This study aimed to test the hypothesis that broad-based coriander genotypes possess unique characteristics through an integrated phenological, agronomic, and chemical analysis approach, thereby providing targeted information for breeding and conservation.

## 2. Materials and Methods

### 2.1. Plant Material

In this study, the fruits of 105 coriander genotypes obtained from the USDA (United States Department of Agriculture), 22 genotypes from Plant Gene Resources of Canada (PGRC), 26 domestic genotypes, 2 cultivars (Arslan and Gürbüz) obtained from Ankara University Faculty of Agriculture, 1 cultivar (Sancarbey) from the Seed Registration and Certification Center Directorate and 4 cultivars (Erbaa, Kudret-K, Gamze, and Pelmus) from the Black Sea Agricultural Research Institute were used. Agricultural and some quality characteristics of a total of 153 genotypes and 7 cultivars were examined under Bolu ecological conditions, in Bolu, Türkiye. In the experiment, 159 different genotypes were sown in the 2024 growing season and adapted to the ecological conditions of Bolu, except for six genotypes (CN-19164, CN-36066, CN-72581, and CN-33961 from Canada; PI 256146 from the USDA; and Denizli-2, a domestic genotype). The list of genotypes and cultivars used in the research is given in Table 1.

### 2.2. Experimental Details

The experiment was established on 17 April 2024, at the Bolu Abant İzzet Baysal University, Faculty of Agriculture Research and Application area (40°44′45″ N latitude and 31°37′46″ E longitude, with an elevation of 752 m above sea level), using an augmented block experimental design [24]. The field experiment was arranged in an Augmented Randomized Complete Block Design (ARCBD). The field layout and within-block randomization were generated using JMP statistical software by applying a uniform random number generation algorithm to ensure unbiased plot allocation within each block (Table S1). The experiment was established in 3 blocks, each containing 53 genotypes and 7 cultivars. Seven registered cultivars were utilized as standard checks (controls) and randomly allocated within each block, while the test genotypes were assigned once across the blocks. The spacing between blocks was 1 m, each block was 18 m long and 4 m wide, with a row spacing of 30 cm and a plant spacing of 10 cm. So, the total experimental area is 252 m^2^ (18 m × 14 m) including pathways. The soil properties of the experimental area were as follows: low in phosphorus (P) (75.80 kg/ha), rich in potassium (K) (947.40 kg/ha) and organic matter (1.06%), clayey and slightly alkaline (pH = 7.56), medium-lime (7.37%), and low salinity (0.04%). Average climatic data were recorded between 13.4 and 22.1 °C for temperature, 0.10 and 77.10 kg/m^2^ for rainfall, and 58.90% and 71.30% for humidity during the vegetation period of 2024 from April to August (Table S2) [25]. In the experiment, the fruits were sown on 17 April 2024, and sowing was conducted using an excessive number of fruits (approximately 50), enabling thinning to be performed 10–15 days after emergence to achieve the desired number of plants in each row. At sowing, 60 kg/ha of diammonium phosphate (DAP) and 25 kg/ha of ammonium sulfate (21% N) were applied as basal fertilizers; at the beginning of flowering, 25 kg/ha of ammonium sulfate was applied again as fertilizer. After the seedling days, irrigation was performed every day as much as the plants needed using the drip irrigation method, and weed control was performed by hand every two days. For morphological and yield values, 10 plants were randomly selected from each row (without excluding border plants), and the plants were harvested between 1 July and 28 August 2024.

### 2.3. Isolation Procedures

Coriander is protandrous and often cross-pollinated. Natural open pollination (cross-pollination) ranged from 25 to 70% depending on the genotype and presence of pollinators [26]. To prevent unwanted cross-pollination and genetic contamination among genotypes, fine-mesh isolation covers were applied row by row immediately prior to anthesis (flowering onset). Since genotypes exhibited variation in phenological development, the onset of flowering occurred progressively between 10 June and 30 June 2024, and mesh covers were installed in a staged manner corresponding to the onset of flowering of each row. During this process, the same genotypes in each row were allowed to undergo free pollination, but gene flow between rows was prevented. The mesh covers allowed sufficient light transmission and air circulation, ensuring microclimatic stability; no adverse effects on plant photosynthesis or generative growth were observed. Successful self-pollination and fruit setting under isolation were visually verified across all genotypes.

### 2.4. Essential Oil Content (%)

A 20 g sample of whole fruits, oven-dried at 35 °C for one week, was subjected to hydro-distillation in 200 mL of distilled water using a Clevenger-type apparatus for 4 h. The essential oil yield percentage was calculated by proportioning the volume of the separated oil (ml) to the initial amount of dry matter [27].

### 2.5. Essential Oil Components (%)

Determination of essential oil components was carried out at the Bolu Abant İzzet Baysal University, Scientific, Industrial and Technological Application and Research Center. Prior to analysis, samples were prepared as a solution by diluting with methanol (Sigma-Aldrich, Darmstadt, Germany) at a ratio of 1:100 [28]. Essential oil component analysis of the samples was performed using a GC/GC-MS (gas chromatography (Thermo Scientific Trace 1300, Rodano, Milano, Italy)-mass detector (Thermo Scientific ISQ QD, Austin, TX, USA) device with a capillary column (TG-624; 30.0 m × 0.25 mm × 1.4 μm). Helium 99.999% purity, La Barge, WY, USA) was used as the carrier gas at a flow rate of 1.00 mL/min, and samples were injected into the device at a rate of 1 μL. The injector temperature was maintained at 230 °C, and the column temperature program was set to 40 °C, then 40 °C to 130 °C at 5 °C/min (3 min), and 130 °C to 220 °C at 10 °C/min (5 min) in splitless mode. The total analysis time was determined according to this temperature program. The experiment lasted 35 min. For the mass detector, a scanning range (*m*/*z*) of 40–500 atomic mass units, electron bombardment ionization of 70 eV, a transfer line temperature of 250 °C, and an ion source temperature of 220 °C were used. The retention time was used to determine the concentration of essential oil composition. Data from the WILEY and NIST libraries were used for the identification of the essential oil components. The percentages of the components and their identification were obtained using an MS detector.

### 2.6. Morphological Characteristics and UPOV Criteria

The morphological characteristics examined in the study were as follows: the dates of 50% seedling emergence, 50% flowering, and 50% fruit setting were recorded as the number of days from sowing to the respective stages through phenological observations. In the experiment, the seedling days of coriander genotypes and cultivars were obtained by recording the day from planting to the date when 50% of the plants in each row were visible above the soil surface. The 50% flowering days were obtained by recording the day between the planting date and the date when flowering was observed in 50% of the plants. The 50% fruit setting days were obtained by recording the day between the planting date and the date when 50% of the plants were observed to have set fruit. Plant height was measured in centimeters from the soil surface to the highest point of 10 randomly selected plants that had reached physiological maturity. The number of branches was recorded as the average number of primary branches emerging from the main stem of 10 randomly selected plants at physiological maturity. The number of umbels was determined by counting the umbels on each of the 10 selected plants and calculating the average. The number of umbellets was calculated as the average number of umbellets per umbel across the 10 selected plants. Thousand-fruit weights were obtained by counting and weighing four replicates of 100 seeds each from the pure seeds of each row using a precision balance; the average of these four values was then multiplied by 10 to express the results in grams. Fruit yield per plant (g) was measured after threshing and cleaning individual plants. The harvest index was calculated using the following formula:

Harvest index (%) = (Fruit yield/Biological yield) × 100

The UPOV criteria examined in the coriander varieties and genotypes were determined according to UPOV standards [28]. The characteristics, scales, and descriptions examined are provided in Table 2.

### 2.7. Statistical Analysis

The data obtained in the study were subjected to analysis of variance (ANOVA) for each characteristic individually according to the augmented experimental design; significance tests were conducted using the F-test, and the grouping of mean differences was performed based on the Least Significant Difference (LSD) method using the AVCI statistical software. The results of the ANOVA by comparison of repeated averages (control coriander cultivars) are given in Table S3. The obtained data were subjected to adjusted mean values (AMV) before LSD grouping because of observing high variance among blocks. The AMV was performed according to Peterson [29] using the following formula:

ŷij = yij − aj, aj = x − x

where ŷij: the adjusted mean of the i-th selection in the j-th block; yij: the value of each character in the i-th selection in the j-th block; aj: the adjustment factor for the j-th block; x: the mean value of all checks in the j-th block; x: the general mean.

In addition, the Least Significant Difference (LSD) values were found by calculating the following formulas according to Peterson [29].

1. Comparison of repeated averages (control coriander cultivars);

LSD = t_0.05_ × (2 × MSE)/j

2. Comparison of two non-replicated means in the same block;

LSD = t_0.05_ × 2MSE

3. Comparison of two non-replicated means in different blocks;

LSD = t_0.05_ × (2j + 1 × MSE)/j

4. Comparison of repeated and non-repeated averages;

LSD = t_0.05_ × ((r + 1)(j + 1)MSE))/rj

where MSE: mean squared error; j = block number; r = control cultivar number;
t_0.05_ = represents the two-way table t-value at the 0.05 degree of freedom error level.

Descriptive statistics, including mean, standard deviation (SD), standard error of the mean (SEm), and minimum and maximum values, were computed for each morphological, yield and chemical property. The 95% confidence intervals (CI) for the genotype means were calculated as follows:

95% CI = Mean ± _tα/2, df_ × SEm

where t_α/2, df_ represents the critical value of Student’s t-distribution at α = 0.05 with n—1 degrees of freedom. The obtained results are presented in Table S4.

In the statistical evaluation, the uppercase letter “A”, the lowercase letter “z”, and numerals were used to indicate the differences between the examined characteristics. The cluster and PCA analyses were carried out using the JMP 13 statistical program.

## 3. Results

### 3.1. Observed Phenological Changes in Coriander Genotypes

The results of the study revealed that different coriander genotypes and cultivars exhibited statistically significant differences in terms of phenological characteristics at a 5% significance level. The data regarding the duration to 50% seedling emergence, flowering and 50% fruit setting days are provided in Table 3.

As seen in Table 3, the 50% seedling emergence days (CV = 1.39%) and the 50% flowering days (CV = 1.16%) showed relatively low to moderate variation, suggesting that these phenological characteristics were comparatively stable across genotypes. However, the 50% fruit setting days (CV = 6.12%) of coriander genotypes and cultivars exhibited slightly higher genotype-dependent variation.

The 50% seedling emergence days of the different coriander genotypes and cultivars varied between 18.95 and 27.10 days. The earliest 50% seedling emergence day was observed in the GN-87 (18.95 days), followed by GN-112 (21.95 days) and GN-130 (21.95 days) genotypes. Conversely, the latest 50% seedling emergence day was recorded in the genotypes GN-21, GN-23, GN-42, GN-79, GN-114, GN-121, and GN-124 (27.10 days). Relative to the overall mean of 23.44 days, 44 genotypes showed values above average, while 116 genotypes fell below.

When compared to the GN-76, GN-104, GN-107, GN-125 and GN-128 genotypes (23.00 days), which had the earliest seedling emergence day among the cultivars, 84 different genotypes had an earlier seedling emergence day, showing superior performance with results ranging from 18.95 to 23.00 days.

Analysis of variance for flowering days revealed a wide range of variation and statistically significant differences among all evaluated coriander genotypes (Table S3). The 50% flowering days among coriander genotypes and cultivars ranged between 55.52 and 74.67 days. The earliest 50% flowering was observed in the GN-52, GN-142 and GN-151 (55.52 days) genotypes originating from Oman, followed by the GN-114 and GN-115 (55.81 days) genotypes. On the other hand, the latest 50% flowering was recorded in the GN-1, GN-14 and GN-154 (74.67 days) genotypes. A total of 9 genotypes reached 50% flowering earlier than the GN-107 cultivar (58.67 days), and these genotypes exhibited a flowering period ranging from 58.67 to 67.67 days.

The 50% fruit setting days (FSD) of the different coriander genotypes and cultivars were found to be between 61.67 and 96.10 days (Table 3). The earliest 50% fruit setting day was observed in the GN-91 (61.67 days) genotype originating from Canada, followed by the GN-116 (64.10 days) genotype. Conversely, the latest 50% FSD was recorded in the GN-1 and GN-10 (96.10 days) genotypes (Table 3). Five genotypes achieved fruit set earlier than GN-160 (69.00 days), exhibiting accelerated generative development with periods ranging from 62.00 to 68.00 days.

### 3.2. Changes of Morphological and Yield Traits

The results of the study revealed that there were statistically significant differences among coriander genotypes and cultivars in terms of plant height (Table 4). The plant height values among coriander genotypes and cultivars varied between 38.73 and 112.03 cm, while the highest plant height was obtained from the GN-87 (112.03 cm) genotype, followed by GN-132 (90.39 cm) and GN-88 (89.63 cm). The lowest plant height was observed in the GN-101 (38.73 cm) genotype. Compared with the GN-125 cultivar (83.00 cm), which had the highest plant height among the cultivars, 9 genotypes exceeded this cultivar in plant height. These genotypes exhibited enhanced vegetative growth performance, with plant heights ranging from 83.00 to 112.03 cm. Plant height (PH) averaged 61.60 cm, with a 95% confidence interval ranging between 59.55 cm and 63.64 cm (Table S4).

Statistically significant differences were found among coriander genotypes and cultivars in terms of the number of branches (Table 4). The values for the number of branches among coriander genotypes and cultivars varied between 1.18 and 10.24. GN-87 (10.24) had the highest number of branches, followed by GN-117 (9.04) and GN-150 (8.79). GN-158 (1.18) had the lowest number of branches, followed by GN-118 and GN-134 (1.29). Relative to the overall mean of 4.87 branches, 64 genotypes had higher values and 96 had lower values. Compared to the GN-128 cultivar, which had the highest number of branches (5.77), 66 different genotypes had more branches than this cultivar, and these genotypes exhibited a higher vegetative branching performance with values ranging from 5.77 to 10.24. GN-87 showed high values for both traits, suggesting a positive association.

The results of the study revealed statistically significant differences among various coriander genotypes and cultivars in terms of the number of umbels (Table 5). The values for the number of umbels per plant among coriander genotypes and cultivars varied between 2.79 and 26.70, with an average value of 8.15. When the overall averages of different coriander genotypes and cultivars were evaluated, 64 genotypes exhibited values above the overall mean, whereas 96 genotypes exhibited values below it. GN-80 (26.70 per plant) had the highest number of umbels, followed by the GN-70 (21.10 per plant); the lowest number of umbels was observed in GN-53 (2.79 per plant) and followed by GN-72 (3.19 per plant). Eight genotypes recorded superior umbel counts (14.39–26.70 per plant) relative to the GN-128 cultivar (13.90 per plant).

Statistically significant differences were found among coriander genotypes and cultivars in terms of the number of umbellets per umbel (Table 5). The number of umbellets per umbel showed considerable variation among coriander genotypes and cultivars, ranging from 4.30 to 117.60. The highest number of umbellets per umbel was observed in GN-71 (117.60), followed by GN-15 (81.20). The lowest number of umbellets was recorded in GN-60 (4.30), followed by GN-136 (8.30) and GN-57 (8.40).

Compared with the GN-107 cultivar, which exhibited the highest number of umbellets (76.97 umbellets per umbel), 8 different genotypes were found to produce even greater numbers of umbellets, demonstrating superior productivity, with values ranging from 76.97 to 117.60 umbellets per umbel. While the study showed significant differences in umbel number among genotypes with a high coefficient of variation (CV = 30.06%), umbellet number exhibited a relatively low coefficient of variation (CV = 23.92%). These findings highlight significant differences among the studied coriander genotypes and emphasize their potential for future selection and breeding programs.

The results of the study revealed that there were statistically significant differences among coriander cultivars and genotypes in terms of thousand-fruit weight (Table 6). The thousand-fruit weight of the different genotypes and cultivars varied from 5.30 to 34.21 g, with an average of 17.31 g. The maximum values were observed in GN-146 (34.21 g) originating from Uzbekistan and GN-65 (29.26 g), while the minimum was found in GN-103 (5.30 g) originating from Türkiye, followed by the GN-100 (5.60 g). Twenty-eight genotypes outperformed the highest-yielding cultivar, GN-104 (28.32 g), exhibiting superior thousand-fruit weights ranging from 28.32 to 34.21 g.

The results revealed that there were statistically significant differences among coriander genotypes and cultivars in terms of fruit yield (Table 6). The fruit yield values among coriander genotypes and cultivars showed quite high variation, ranging from 0.05 to 4.98 g/plant. The highest fruit yield was obtained from GN-37 (4.98 g/plant) originating from Kazakhstan, followed by the GN-103 (3.82 g/plant) originating from Türkiye, and GN-33 (3.71 g/plant) originating from England genotypes. The lowest fruit yield was observed in the GN-89 (0.05 g/plant) originating from Canada. Compared with the GN-104 cultivar (1.85 g/plant), 14 different genotypes exhibited significantly greater fruit yields, indicating superior productive potential, with yields ranging from 1.89 to 4.98 g/plant.

The results revealed that there were statistically significant differences among coriander cultivars and genotypes in terms of biological yield (Table 6). Biological yield varied widely among genotypes, ranging from 0.97 to 49.24 g/plant. The highest biological yield was observed in the GN-114 (49.24 g/plant) originating from Pakistan, followed by GN-156 (16.08 g) originating from India. The lowest biological yield was seen in GN-142 (0.97 g) originating from Pakistan. One genotype outperformed GN-128 (7.57 g/plant), demonstrating superior biological yield (7.57–49.24 g/plant). Table S4 revealed that fruit yield showed a mean of 1.22 g/plant (95% CI: 1.12–1.33 g/plant), whereas biological yield exhibited a mean of 5.44 g/plant (95% CI: 4.81–6.07 g/plant).

The results revealed that there were statistically significant differences among coriander cultivars and genotypes in terms of harvest index (Table 6). The harvest index values among coriander genotypes and cultivars varied between 0.32% and 46.13%. The highest harvest index was observed in GN-1 (46.13%) originating from Israel, followed by GN-38 (35.71%) originating from Azerbaijan. The lowest harvest index was seen in the GN-11 (0.32%) originating from the Czech Republic. While the GN-128 cultivar exhibited the highest harvest index value among the cultivars (8.83%), 25 different genotypes significantly exceeded this value, producing results ranging from 8.83% to 46.13%.

In addition, the fruit yield (CV = 61.71%) and biological yield (CV = 45.24%) displayed the highest variability, whereas the harvest index values (CV = 27.33%) and the thousand-fruit weight values (CV = 29.81) of coriander genotypes and cultivars exhibited relatively low to moderate genotype-dependent variation.

### 3.3. Analysis Results of Essential Oil and Essential Oil Components

The results revealed that there were statistically significant differences among coriander cultivars and genotypes in terms of essential oil content (Table 7). The results of this study demonstrate variability in the essential oil content of coriander genotypes and cultivars of different origins grown under the same ecological conditions. Essential oil composition showed the widest range of variability, with β-pinene (CV = 79.77%), linalool (CV = 47.90%), trans-geraniol (CV = 54.65%), ɣ-terpinene (CV = 47.90%), 13-tetradecenal (CV = 55.28%), myristic acid (CV = 48.55%) exhibiting particularly high CV values, indicating pronounced differences among genotypes. In contrast, essential oil content (CV = 23.25%) was comparatively more stable.

Essential oil content of different coriander genotypes and cultivars showed a significant variation, ranging from 0.02% to 2.25%. The highest essential oil content was observed in GN-154 (2.25%) originating from India, followed by GN-88 (1.65%), GN-118 (1.55%) and GN-123 (1.52%) genotypes. The lowest essential oil content was recorded in GN-102 (0.02%) originating from Canada. Fifty-five genotypes significantly exceeded the essential oil yield of GN-128 (0.75%), displaying superior values ranging from 0.76% to 2.25%.

In the experiment, the most important essential oil components of coriander genotypes and cultivars were β-pinene, linalool, **γ**-terpinene, camphor, trans-geraniol, trans-geranyl acetate, 13-tetradecenal, and myristic acid. The percentages of these eight components are given in Table S6, and the percentages of other essential oil components are given in Table S10.

The linalool content of coriander genotypes and cultivars exhibited significant variation, ranging from 0.52% to 48.90%. The highest linalool levels were observed in the GN-114 (48.90%) originating from Pakistan, followed by the GN-73 (47.69%) originating from Türkiye. The lowest linalool level was observed in the GN-2 (0.52%) genotype, followed by GN-3 (0.65%). Linalool content recorded a mean of 24.44% (95% CI: 23.16–25.71%), ranging from 0.52% to 48.90% across the evaluated genotypes (Table S4).

The β-pinene values among coriander genotypes and cultivars varied between 0.04% and 23.73%. The genotype with the highest β-pinene ratio was the GN-128 (23.73%) cultivar, followed by the GN-106 (16.14%) and GN-107 (16.12%) cultivars. The lowest β-pinene ratio was observed in the GN-90 (0.04%) originating from Canada. Compared to the GN-104 cultivar (8.69%), which had the highest β-pinene content among the cultivars, 16 different genotypes exceeded this commercial value. However, genotypes such as GN-128 (23.73%), GN-106 (16.14%) and GN-107 (16.12%) stood out as having the highest β-pinene potential within the germplasm, following GN-104, which showed the highest performance among commercial cultivars.

The **γ**-terpinene ratios among coriander genotypes and cultivars varied between 0.31% and 26.87% (Table 7). The GN-106 (26.87%) cultivar had the highest **γ**-terpinene value, followed by GN-107 (24.25%) and the GN-128 (18.44%) cultivar. The lowest **γ**-terpinene ratio was observed in the GN-34 (0.31%) originating from England.

The mean values of camphor levels among coriander genotypes and cultivars varied between 0.03% and 12.56% (Table 7). The highest camphor level was obtained in GN-19 (12.56%) originating from the Czech Republic, followed by the GN-120 (12.35%) originating from Türkiye. The lowest camphor level was observed in the GN-134 (0.03%) genotype, followed by GN-46 (0.08%).

The mean values of *trans*-geraniol ratios among coriander genotypes and cultivars varied between 0.05% and 9.51%. The genotype with the highest *trans*-geraniol ratio was GN-19 (9.51%) originating from the Czech Republic, followed by GN-133 (8.81%) and GN-80 (7.75%). The lowest *trans*-geraniol ratio was observed in GN-150 and GN-151 (0.05%), followed by GN-34.

The mean *trans*-geranyl acetate ratios among coriander genotypes and cultivars varied between 0.15% and 24.90%. The genotype with the highest trans-geranyl acetate ratio was GN-84 (24.90%) originating from Canada, followed by the GN-152 (10.95%) originating from India. The lowest trans-geranyl acetate ratio was observed in the GN-9 (0.15%) genotype, followed by GN-34 (0.17%).

The mean 13-tetradecenal values among coriander genotypes and cultivars varied between 0.02% and 12.58%. The genotype with the highest 13-tetradecenal ratio was GN-31 (12.85%) originating from Poland, followed by the GN-23 (10.51%) originating from Russia. The lowest 13-tetradecenal ratio was observed in GN-53 (0.02%), followed by GN-72 and GN-103 (0.14%).

Myristic acid ratios among coriander genotypes and cultivars varied between 0.01% and 23.61%. The genotype with the highest myristic acid ratio was GN-53 (23.61%) originating from Oman, followed by the GN-140 (19.10%) originating from Afghanistan and GN-134 (16.09%). The lowest myristic acid ratio was observed in the GN-46, GN-61 and GN-73 (0.01%) genotypes.

### 3.4. Identification of UPOV Criteria

When the coriander genotypes and cultivars were evaluated in terms of UPOV criteria, the anthocyanin coloration of the hypocotyl was found to be absent or very weak (1) in all genotypes and cultivars (Table S11). The cotyledon shape was determined as narrow elliptic (1) in 75 genotypes and 4 cultivars (GN-76, GN-125, GN-128, GN-160), elliptic (2) in 77 genotypes and 3 cultivars (GN-104, GN-106, GN-107), and broad elliptic (3) in one genotype. Plant height was found to be short (3) in 70 genotypes and 5 cultivars (GN-76, GN-104, GN-107, GN-125, GN-160), medium (5) in 32 genotypes and 1 cultivar (GN-128), and tall (7) in 51 genotypes and 1 cultivar (GN-106). The number of basal leaves was found to be few (3) in 1 genotype, medium (5) in 25 genotypes and 1 cultivar (GN-125), and many (7) in 127 genotypes and 6 cultivars (GN-76, GN-104, GN-106, GN-107, GN-128, GN-160). Leaf density was determined as medium (5) in 36 genotypes and 2 cultivars (GN-104, GN-125) and high (7) in 117 genotypes and 5 cultivars (GN-76, GN-106, GN-107, GN-128, GN-160). The green color of the plant was found to be light green (1) in 5 genotypes, green (5) in 39 genotypes and 2 cultivars (GN-104, GN-128), and dark green (7) in 109 genotypes and 5 cultivars (GN-76, GN-106, GN-107, GN-125, GN-160). Leaf pubescence was found to be present (1) in all genotypes and cultivars. Terminal leaf size was determined as small (3) in 2 genotypes, medium (5) in 36 genotypes and 2 cultivars (GN-125, GN-128), and large (7) in 115 genotypes and 5 cultivars (GN-76, GN-104, GN-106, GN-107, GN-160). The density of leaf serration was found to be sparse (3) in 3 genotypes and 1 cultivar (GN-125), medium (5) in 22 genotypes, and dense (7) in 128 genotypes and 6 cultivars (GN-76, GN-104, GN-106, GN-107, GN-128, GN-160). Leaf margin attitude was determined as straight (3) in 27 genotypes and 1 cultivar (GN-125) and upward (5) in 126 genotypes and 6 cultivars (GN-76, GN-104, GN-106, GN-107, GN-128, GN-160). Petiole length was found to be short (3) in 5 genotypes, medium (5) in 73 genotypes and 5 cultivars (GN-104, GN-106, GN-125, GN-128, GN-160), and long (7) in 75 genotypes and 2 cultivars (GN-76, GN-107). Anthocyanin coloration of the flower was absent (1) in all genotypes and cultivars. Fruit size was determined as small (3) in 33 genotypes and 1 cultivar (GN-107), medium (5) in 61 genotypes and 4 cultivars (GN-76, GN-104, GN-106, GN-160), and large (7) in 59 genotypes and 2 cultivars (GN-125, GN-128). The intensity of brown color on the fruit was found to be light (1) in 25 genotypes and 1 cultivar (GN-128), medium (5) in 100 genotypes and 6 cultivars (GN-76, GN-104, GN-106, GN-107, GN-125, GN-160), and dark (7) in 28 genotypes. Fruit shape was determined as medium elliptic (1) in 80 genotypes and 3 cultivars (GN-104, GN-125, GN-128), broad elliptic (2) in 44 genotypes and 3 cultivars (GN-76, GN-107, GN-160), and circular (3) in 29 genotypes and 1 cultivar (GN-106). The time of beginning of flowering was found to be early (3) in 67 genotypes and 3 cultivars (GN-76, GN-125, GN-160), medium (5) in 39 genotypes and 3 cultivars (GN-104, GN-106, GN-107), and late (7) in 47 genotypes and 1 cultivar (GN-128).

### 3.5. Identification of the Genotype Differences by Cluster Analysis

**Figure 1:**
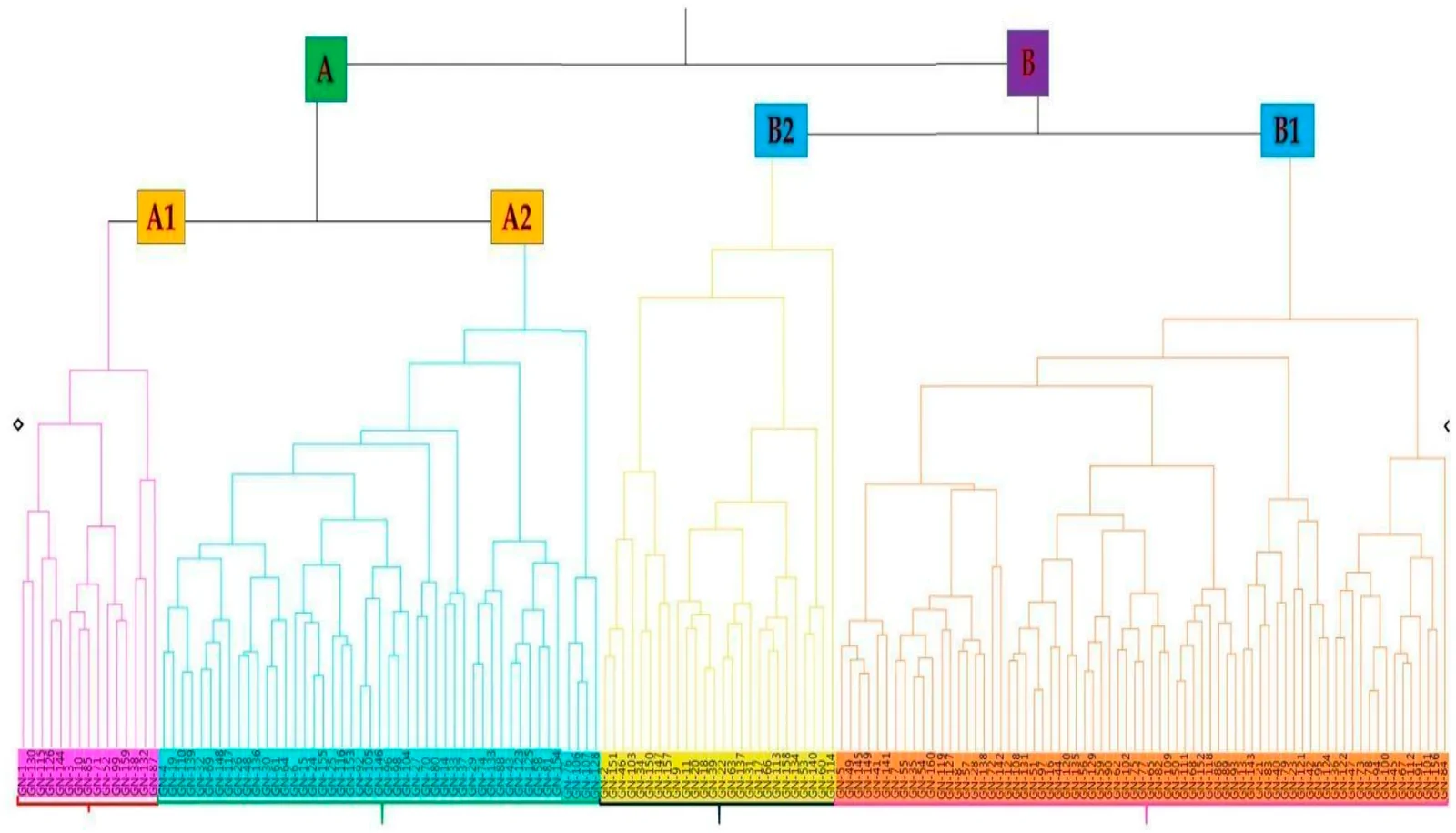
Cluster analysis result of the examined traits in coriander genotypes of different origin.

The cluster analysis was conducted to determine the relationships among some important traits (plant height, branch number, umbel number, umbellet number, biological yield, harvest index, 1000 fruit weight, fruit yield, essential oil content and major essential oil compositions) of different coriander genotypes. Also, the differences and groupings among coriander genotypes and cultivars were shown in a dendrogram based on data obtained from the cluster analysis. In general, the cluster analysis consisted of two main groups, A and B, and each main group contained subgroups A1, A2, B1 and B2 (Figure 1).

The information about the groups is summarized as follows: The A main group: This group is composed of 56 genotypes and six cultivars (GN-76, GN-104, GN-106, GN-107, GN-125 and GN-128). Plant height and branch number traits were the dominant separation parameters compared to the B main group. This main group was divided into two subgroups (A1 and A2). The A1 subgroup contained 15 genotypes. One of the three Tajikistan-origin genotypes was found in this group. In addition, GN-1 (originated from Israel) was noted in the same group as GN-130 (originated from Türkiye) because of their high harvest index values.

The A2 group contained 41 genotypes of different origin, such as GN-4 (Egypt), GN-26 (France), GN-29 (Poland), GN-43 (Russia), GN-54 (Oman) and GN-105 (Türkiye) and six cultivars. The GN-14 (Japan), GN-33 (England) and GN-37 (Kazakhstan) genotypes, which had the highest fruit yield values, were found in this subgroup. Similarly, the GN-154 originating from India, which had the highest essential oil content (2.25%), was detected in the A2 subgroup.

The B main group: It was noted that this main group, in which most of the genotypes were clustered, contained 97 genotypes and a cultivar (GN-160; Sancarbey) among the main groups according to the result of the cluster analysis. This group contained two-thirds of the genotypes originating from Ethiopia. The camphor and trans-geraniol among the essential oil components emerged as key factors in the separation of this group. The B main group was divided into two subgroups, B1 and B2, and most of the genotypes were found in the B1 subgroup. The GN-103 (Denizli, Türkiye) genotype, which had the second highest fruit yield value, was found in this subgroup. The B2 subgroup contained 26 genotypes of different origin, and two-thirds of the Tajikistan genotypes (GN-150 and GN-151) were found in this group.

### 3.6. Comparing Traits Examination by Correlation Analysis and Principal Coordinate Analysis (PCA)

Correlation analysis was conducted to determine the relative importance of each trait and its associations with other traits. The analysis revealed 36 significant correlations among the phenological, morphological, yield and essential oil content and composition traits (Table 8). The highest significant positive correlations were found between ɣ-terpinene and β-pinene (r = 0.775 **), and between *trans*-geranoil and camphor (r = 0.743 **). Fruit setting days were correlated positively with umbellet number (r = 0.202 *). Similarly, β-pinene was positively correlated with trans-geraniol with r = 0.202 *. The highly significant negative correlations were found between 13-tetradecenal and *trans*-geraniol. Moreover, myristic acid was highly significantly correlated with camphor (r = −0.255 **). Seven negative correlations were found among the traits, and myristic acid was negatively correlated with β-pinene. As a result, Table 8 shows that the lack of significant correlation between fruit yield and both essential oil content (r = 0.131, *p* > 0.05) and linalool content (r = 0.107, *p* > 0.05) indicates that these traits are inherited independently. This study suggests that high fruit yield and superior essential oil quality can be improved simultaneously in coriander breeding programs without compensating for one another. Furthermore, correlation analysis was performed between the UPOV descriptors and major essential oil constituents, excluding hypocotyl anthocyanin coloration, leaf pubescence, and leaf anthocyanin coloration, which were uniform across all genotypes and cultivars (Table 9). The results revealed a total of 64 correlations: 34 highly significant positive, 10 highly significant negative, 11 significant positive, and 9 significant negative correlations. Twenty-seven correlations were found among the UPOV descriptors. The highest significant positive correlations were found between the number of basal leaves and leaf density (r = 0.796 **), and petiole length showed the strongest significant negative correlation with 13-tetradecenal content (r = −0.343 **). In addition, β-pinene and trans-geraniol showed a positive correlation (r = 0.202 *), whereas β-pinene and myristic acid showed a negative correlation (r = −0.202 *). Moreover, positive correlations were found between petiole length and camphor, and negatively correlated with 13-tetradecenal. The time at which flowering began affected the essential oil composition of coriander. In particular, this criterion was positively correlated with β-pinene, ɣ-terpinene, camphor, and *trans*-geraniol, and negatively correlated with 13-tetradecenal and myristic acid. Similarly, the intensity of brown color in fruit criteria had a negative correlation with 13-tetradecenal, and fruit shape showed a positive correlation with myristic acid.

In the study, PCA was performed to group the coriander genotypes based on selected morphological, yield, essential oil content, and essential oil composition traits, and to investigate the relationships among them. In total, 17 PC axes were found with eigenvalues higher than 1, however two PC axes were selected for interpretation.

**Figure 2:**
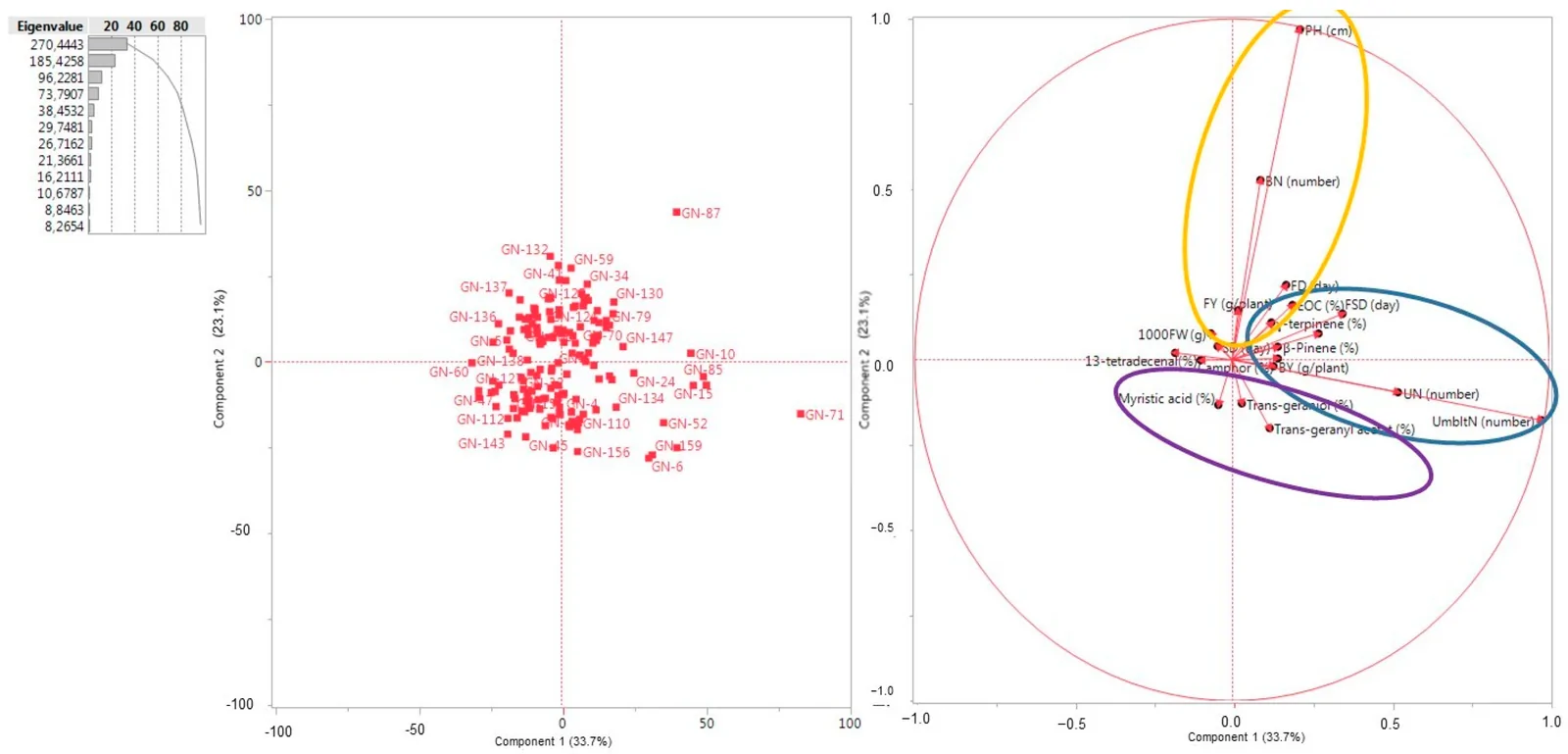
PCA results for the coriander genotypes and examined properties. PH: plant height; BN: branch number; UN: umbel number; UmbltN: umbellet number; BY: biological yield; HI: harvest index; EOC: essential oil content.

The Principal Component Analysis (PCA) revealed that the first two components accounted for 56.8% of the total variation (33.7% for PC1 and 23.1% for PC2) with eigenvalue greater than 1 based on the examined traits (Figure 2). PC1 was strongly associated with yield components such as number of umbels (UmbltN) and umbellets (UN), alongside essential oil content (EOC), whereas PC2 was predominantly defined by vegetative traits including plant height (PH) and branch number (BN). Moreover, PCA results showed that camphor, biological yield, and umbellet number were the most stable traits that can be used for breeding programs in the studied coriander genotypes.

The PCA distribution highlighted genotypes GN-71 and GN-87 as distinct outliers; GN-71 exhibited superior performance for umbel-related parameters and essential oil traits, while GN-87 combined high morphological and yield values, making them valuable parental genotypes for future breeding strategies.

## 4. Discussion

### 4.1. Phenological Characteristics

Phenological observations obtained from different genotypes provide valuable insights into the variation associated with plant developmental stages. Such observations contribute to a better understanding of genotype-specific responses and provide important information for assessing environmental adaptation and yield potential. Phenological characteristics, including seedling, flowering and fruit set time, enable comprehensive comparisons among genotypes and facilitate the identification of superior lines with desirable traits for breeding programs. Furthermore, the evaluation of these characteristics plays a crucial role in elucidating genotype-specific responses to environmental stresses, particularly those associated with climate change, and contributes to the development of appropriate and sustainable cultivation strategies.

The results obtained from this study for the 50% seedling emergence days were comparable to those reported in previous studies. Specifically, Karadoğan and Oral [30] reported a range of 22.50 to 31.00 days, and Basol et al. [23] observed between 24.37 and 40.97 days. However, our results showed later seedling emergence than those of the spring sowing on May 12 and the winter sowings on October 20, November 8, and November 21 reported by Yurum [31] (14.00–20.00 days). It is considered that the differences in 50% seedling emergence durations stem from variations in sowing times, genotypic differences, climatic conditions, and cultivation practices.

The 50% flowering days across coriander genotypes and cultivars compared to findings by other researchers, the results were similar to those obtained from the spring sowings of Karadoğan and Oral [30] (51.10–76.60 days), Kalkan [32] (59.17–68.38 days), and Basol et al. [23] (52.40–72.20 day). However, the 50% flowering durations were earlier than those reported by Yurum [31] (61.00–67.00 days) for winter cultivation. This difference is thought to arise from variations in genotypes and sowing times. The 50% FSD of the coriander cultivars and genotypes were found to be quite close to the values obtained by Basol et al. [23] (61.40–96.20 days) in spring sowings.

When the phenological characteristics examined in the present study were compared with those reported in the literature, these differences could be explained by variation in temperature, light intensity, and competition for resources between reproductive and vegetative tissues [33]. Moreover, the environmental conditions, plant genes and their interactions affect the number of days to flowering [34].

### 4.2. Morphological and Yield Traits of Coriander

The assessment of morphological characteristics and yield components across different genotypes enables the precise characterization of genetic variation within the germplasm and the selection of superior genotypes. Such assessments are fundamental to improving selection efficiency and accelerating genetic gains for target traits, thereby supporting the development of high-performing and agronomically desirable genotypes in breeding programs. When the plant heights of coriander genotypes and cultivars were compared with other studies, they showed similarity with the findings of Emiralioğlu and Yaldız [35] (25.50–84.30 cm), and the findings of Kalkan [32] (52.67–65.73 cm), whereas they were found to have lower values than the winter sowing findings of Çınarlıdere [36] (39–77 cm). These differences are likely attributable to ecological and genotypic variation, differences in sowing time, and variations in agronomic practices. Additionally, genotypes exhibiting later flowering and fruit set, as well as a greater number of branches, were observed to have greater plant height.

When the number of branches of the coriander genotypes and cultivars was compared with those reported in previous studies, the values obtained in the present study were higher than those reported by Sezek [37] (4.66–6.08 number) for spring and winter sowing, Emiralioğlu and Yaldız [35] (3.60–9.60 number) and Altun [38] (5.64–6.68 number). However, these results were comparable to the findings of Özyazıcı [39] (5.32–9.03 number) and Shiwangi et al. [40] (4.67–15.20 number).

It is thought that the differences in the number of branches in our study were influenced by the abundance and diversity of genotypes, along with ecological conditions and various applications. Additionally, it was determined that the number of branches was higher in genotypes with late seedling and flowering durations.

Compared with the results of other studies, the number of umbels per plant of coriander genotypes and cultivars was lower than that reported by Altun [38] (28.44–29.76 number) and Basol et al. [23] (2.81–72.68 number). In addition, our result was partly similar to that of Chandrakala et al. [18], who reported that the number of umbels per plant ranged from 8.05 to 20.15, with an average value of 12.69.

The number of umbellets obtained in this study exceeded those reported by Sezek [41] (16.43–19.88), Chandrakala et al. [18] (14.98–35.63), and was lower than the number reported by Basol et al. [23] (0.19–380.19). It is thought that the reasons for these differences are growing conditions, genotypic variations, and variability in sowing times.

When the thousand-fruit weight values of coriander genotypes and cultivars were compared with those reported in previous studies, our results were found to be consistent with the findings of Basol et al. [23] (1.34–21.49 g), whereas our values exceeded those reported by Özel et al. [41] (8.1–11.4 g), Özyazıcı [42] (6.45–9.34 g) and Gücük [43] (7.4–11.9 g) in their spring and winter sowings. This can be explained by the growing conditions, soil properties, ecological conditions, and genotype differences.

The reason for this variation in yield-related traits, such as seed yield and thousand-seed weight, is that the genotypes evaluated in the study were obtained from different countries and quite different coriander growing areas, representing variable soil and environmental conditions. Furthermore, as seen in the yield results, the prominence of genotypes from Kazakhstan, Türkiye and England, which have different ecological and climatic characteristics, indicates that genetic factors predominate.

The fruit yield values obtained in this study were found to be lower than those reported by Basol et al. [23] (0.02–9.58 g/plant), Chandrakala et al. [18] (2.52 to 10.97 g/plant) and Dharmatti et al. [44] (3.60 and 6.93 g/plant) among 21 genotypes of coriander from India and Hanifei et al. [45] (1.14–10.03 g). The production of non-staple fruits and vegetables can be limited by pollen limitation when they have diverse pollination requirements and insufficient pollinator communities [46]. In other words, pollen limitation results from pollinator abundance and flower visitation rates, and shows a positive relationship between the yield value and pollinator visitation [47]. Also, although the genotypes were isolated with a fine white mesh fabric in a way that would not negatively affect generative growth, differences in light intensity, photoperiod, and root zone temperature resulting from the isolation process may have affected fruit yield [17]. Thus, the low fruit set is thought to have resulted from the isolation treatment, in which plants were covered with a fine-mesh fabric prior to flowering to prevent cross-pollination. This covering likely restricted access by pollinating insects, particularly bees, which are essential for effective pollen transfer. Consequently, reduced pollinator activity impaired fertilization, leading to a lower fruit set. In addition, this variation may depend on the soil and climatic characteristics of the region where the research was conducted, the amount of precipitation, and genotypic differences. Despite being grown under identical ecological conditions, different genotypes may exhibit considerable variation in biological yield due to inherent differences in their genetic makeup, growth potential, resource-use efficiency, and biomass accumulation capacity. Genotypes can differ in leaf area, light utilization efficiency, tolerance to ecological conditions such as temperature, drought, and light, and photosynthetic rates, all of which can create variations in biological yield. Furthermore, late-maturing genotypes, which utilize water more efficiently, can grow longer and produce higher biological yields [33]. Therefore, one genotype may have a very high biological yield under the same ecological conditions, while another may have a lower yield.

When the biological yield values of coriander genotypes and cultivars were compared with other studies, it was determined that they were partly similar to the findings obtained by Basol et al. [23] (0.01–50.78 g/plant) but lower than the finding obtained by Thakur and Thakur [48] (31.54–60.61 g/plant). This variation is thought to stem from the differences in ecological conditions and genotypes. Nowak and Szemplinski [49] reported that sowing date affected the coriander yield and quality traits. In addition, they also determined that environmental conditions (precipitation and temperature) influence the yield and yield components of the coriander.

When the harvest index values of coriander genotypes and cultivars were compared with other studies, it was determined that they were lower than the findings of Basol et al. [23] (8.48–73.36%) and Emiroğlu and Yaldız [35] (6.30–60.00%), similar to the findings of Erdoğdu [50] (28.80–32.90%) and Gök [51] (29.00–42.00%); and higher compared to the findings of Thakur and Thakur [48] (6.55–11.47%) and Kandemir [52] (0.28–0.38%). It is thought that the reason for this variation is due to ecological conditions and genotypic differences. In this study, the wide variation in yield and yield characteristics between local genotypes and genotypes from different origins strengthens breeding efforts. Many studies have also reported similarly large differences in yield and yield characteristics among coriander genotypes [18,23].

### 4.3. Essential Oil Content and Compositions

The essential oil content of coriander fruits is the most important factor determining quality in coriander, and hot weather during fruit ripening increases the essential oil content [20]. Therefore, the fact that the average temperature in July, coinciding with fruit ripening in the vegetation period, was 2.3 °C higher than the long-term average increased the essential oil content of the genotypes (Table S1). Furthermore, the high variability in essential oil content among genotypes can be attributed to the genetic structures and environmental adaptations of broad-based coriander genotypes of different geographical origins [22]. When the studies were examined, our essential oil results (0.02–2.25%) were found to be higher than the values reported by Dobos and Novak [53] (0.2–1.3%) in 36 different coriander accessions from Austria, ref. [54] (0.40–0.55%), ref. [55] (0.17–0.33%), ref. [18] (0.08–0.56%), ref. [23] (0.05–1.86%), and ref. [45] (0.25–0.99%).

Comparison with the available literature revealed that the linalool concentrations detected were generally lower than those previously reported by Rashed and Darwesh [56] (65.1–89.41%) and Kamal et al. [57] (51.40–79.80%). However, the observed values were partially comparable to those reported by Basol et al. [23], who reported the linalool values between 3.13 and 45.70% in 114 coriander genotypes and five cultivars, as well as to the findings of Alamrani et al. [58] (48.41%). Conversely, the linalool concentrations determined in the present study were higher than those reported by Bhuiyan et al. [59] (37.70%) and Sumalan et al. [60] (45.38%). In the study, it was observed that the linalool content of the genotypes was generally low. This may be because, compared with long-term rainfall data (28.70 kg/m^2^) for the experimental area, significant rainfall (63.20 kg/m^2^) occurred during July, which coincided with fruit maturation and harvest (Table S2). Indeed, Sangwan et al. [61] stated that climatic conditions such as cloudy days, low temperatures, and heavy rainfall during coriander fruit maturation have a negative impact on the accumulation of linalool levels.

Linalool has been found to possess numerous biological properties, including antimicrobial, sedative, anticonvulsant, anticancer, anti-inflammatory, antidepressant, neuroprotective, hepatoprotective, and lung-protective activities [62,63]. Therefore, GN-114 and GN-73 genotypes may play a significant role in enhancing numerous medicinal effects and healthy living. When the β-pinene ratios of coriander genotypes and cultivars were compared with other studies, they were higher than the findings of Basol et al. [23] (0.10–2.68%), Emiralioğlu and Yaldız [35] (0.09–5.38%) and Altun [38] (1.00–1.42%). It is thought that this variation stems from the ecological conditions of the region where the research was conducted and genotypic differences. When the **γ**-terpinene contents of the coriander genotypes and cultivars were compared with those reported in previous studies, our results were higher than those reported by previous researchers, which ranged from 0.30 to 11.20% [64], (0.05–15.60%) [23], (1.57–9.16%) [38], (0.04–13.8%) [35] and (14.40%) [59]. Since γ-terpinene is known as a potential biofuel alternative [64,65], in this study, the GN-106 and GN-107 varieties, which have the highest **γ**-terpinene values, can be evaluated as biofuel alternatives. The camphor ratios of coriander genotypes and cultivars were particularly higher than the findings of Delibaltova [66] (4.00–8.40%), Chen et al. [67] (0.90–4.90%), and Nguyen et al. [68] (6.98%). However, the amount of camphor obtained in this study is consistent with the findings reported by Basol et al. [23] (0.05–11.18%). Chen et al. [69] reported that the camphor compound has insecticidal, antimicrobial, antiviral, anticoccidial, and antinociceptive effects. So, GN-19 and GN-120 genotypes are a good source of pharmacological properties and biological activities. Compared with previous studies conducted under different ecological conditions, the *trans*-geraniol ratio values obtained in our study were higher than those reported by Chen et al. [67] (0.50–3.00%), Bhuiyan et al. [59] (1.90%) and Al amrani et al. [58] (2.79%). It is thought that this variation stems from the ecological conditions of the region where the research was conducted and genotypic differences. Chen et al. [69] demonstrated that geraniol exhibits both insecticidal and repellent activities. Accordingly, the GN-19 and GN-133 genotypes, characterized by their high *trans*-geraniol content, may constitute promising candidates for the sustainable management of insect pests. Compared with previous studies conducted under different ecological conditions, the *trans*-geranyl acetate values obtained in our study were higher than those reported by Bhuiyan et al. [59] (17.60%), Gandova et al. [70] (5.05%), and Farooq et al. [71] (4.99%). Al-Khayri et al. [19] reported that the values of linalool and geranyl acetate, the main components of coriander, varied according to geographical location, and that the essential oil components of coriander grown under different ecological conditions showed significant variation. Therefore, comprehensive information and research on the essential oil content of coriander genetic resources grown under different ecological conditions are necessary.

Shahwar et al. [72] and Quintans-Júnior et al. [73] reported that geranyl acetate possesses antinociceptive activity. Therefore, the GN-84 and GN-152 genotypes may represent promising genetic resources for natural medicine. El-Soud et al. [74] found that linalool, geranyl acetate, and γ-terpinene in coriander essential oil could significantly reduce serum glucose and increase glutathione peroxidase levels in diabetic mice. In addition, the components of coriander essential oil, namely α-pinene, camphor, geraniol, geranyl acetate, γ-terpinen, linalool, and p-cymene, were found to be effective in reducing liver levels of aspartate aminotransferase and alanine aminotransferase. So, the GN-84 and GN-152 genotypes, as well as the GN-128 and GN-106 cultivars, are important for natural medicine and healthy living.

The 13-tetradecenal content obtained in the present study was higher than that reported by Türkmen and Mert [75], which ranged from 2.96 to 7.00% under different nitrogen doses. Likewise, Basol et al. [23] reported that the 13-tetradecenal ratios of different coriander populations ranged from 0.44 to 10.39%. In contrast, Inan et al. [76] found lower 13-tetradecenal content in six studied coriander cultivars, ranging from 0.02 to 0.15%. The myristic acid content obtained in the present study was significantly higher than that reported by Manhart [20], who observed myristic acid content ranging from 0.10 to 0.45% for the five tested coriander cultivars.

The variations in essential oil components between the current and previous studies can be attributed to factors such as the plant chemotypes, the plant part used, harvest time, geographical origin, environmental factors (including climatic conditions), and agronomic practices, as all of these can influence essential oil components and structure. Previous studies have shown that soil and environmental factors, prevailing plant health conditions, and crop management practices greatly influence the essential oil content and its constituents [18]. Contrary to the findings above, Msaada et al. [77] reported that the concentrations of α-pinene, limonene, sabinene, cis-dihydrocarvone, γ-terpinene, and geranial were not affected by regional factors, and emphasized the necessity of further research across diverse ecological conditions to fully evaluate essential oil variability. In this study, coriander genotypes originating from very different climatic and soil conditions were cultivated under the same ecological conditions, resulting in the expected variation in essential oil content traits and their compositions. So, these findings reveal significant differences among the 160 coriander genotypes in essential oil content and the major components of their essential oils, highlighting their potential for future selection, breeding, and conservation programs.

### 4.4. Correlation Analysis and Principal Coordinate Analysis (PCA)

In this study, the same origin genotypes showed differences in the UPOV criteria. This may be explained by the fact that UPOV criteria can show differences across different areas or growing seasons, because the measurement of the UPOV criteria may be influenced by environmental conditions and growing conditions [78]. Sharangi et al. [15] reported that fruit yield per plant was correlated with the primary branch number, umbel and umbellet number in 14 coriander genotypes. However, our results showed that no correlations were found between fruit yield and other traits, except for trans-geraniol essential oil composition. This may indicate that the evaluated traits were not closely associated with the fruit yield performance. A previous study reported that positive correlations were found among α-pinene, γ-terpinene and camphor [79]. However, in our study, camphor content was correlated with β-pinene and ɣ-terpinene. In light of these findings, experiments should be conducted under different geographical multiyear and growing conditions. The results indicated that a combination of high fruit yield and high linalool content was not achieved in a single coriander genotype. Therefore, advanced breeding strategies, such as molecular markers or recombinant breeding, are required to overcome this constraint. In this context, Hanifei et al. [45] reported that fruit yield and chemical characteristics, particularly essential oil content and fatty acids, represent the primary objectives in coriander breeding. However, to achieve substantial genetic progress, researchers need to understand the nature of gene action and quantify heterosis as well as inbreeding depression.

## 5. Conclusions

This study provides a comprehensive evaluation of 160 coriander genotypes originating from diverse geographical regions, focusing on morphological traits, yield parameters, and essential oil components under uniform agro-ecological conditions. Significant differences were found among the coriander genotypes from different origins based on plant height, umbel development, fruit yield, and essential oil characteristics. GN-91 can be selected for early fruit setting day. Generally, the GN-37, GN-103, and GN-33 genotypes had the highest fruit yield values, and these genotypes can be selected for use in coriander breeding programs. The essential oil composition was characterized by β-pinene, linalool, γ-terpinene, camphor, trans-geraniol, trans-geranyl acetate, 13-tetradecenal, and myristic acid, consistently identified as the predominant constituents across all evaluated genotypes. These phytochemicals are an important criterion for the selection and breeding of genotypes in terms of their functional, industrial and pharmacological applications. The GN-114 and GN-73 genotypes can be selected to obtain the highest linalool content, and the GN-154 genotype can be recommended for the highest essential oil content. The highest values for the evaluated traits were observed in different coriander genotypes, indicating that simple individual plant selection is insufficient. Therefore, it is essential to elucidate the nature of gene action and quantify heterosis as well as inbreeding depression in these genotypes using appropriate breeding methodologies.

The results of the cluster analysis revealed that the genotypes were divided into two main groups. The analysis results can be used in different breeding studies for specific traits examined. For the highest fruit yield and essential oil content, group B can be examined. The PCA represented over 65% in the first two PC axes.

The results obtained showed that this gene collection can be used by breeders to identify genotypes with desirable traits for inclusion in coriander breeding programs. Moreover, genotypes exhibiting high or comparable values to those of the standard cultivars can be considered for use in breeding programs. Hence, new suitable cultivars can be brought to the market. In conclusion, given the significant differences observed among genotypes from different origins in terms of the traits examined, further evaluations under various environmental conditions and over multiple years are needed to achieve more reliable and stable results.

## 6. Limitation and Future Prospects

While this study provides valuable insights, several limitations remain. Since the tested genotypes and cultivars were grown during a single vegetation period, genotype–environment interactions were limited to one year. However, variation in yield and quality can be affected by the interannual performance of the genotypes [80,81]. Indeed, the findings show that genotype evaluation in multiple seasons is important to assess performance consistency. Another limitation of our study concerns the extent to which the fine-mesh isolation method we used to prevent cross-pollination limited yield. For this, comparisons should be made between cultivation under isolation and controlled cultivation in the same year using sufficient seeds obtained from this study. In addition, the evaluation of the genetic variation in coriander genotypes using morphological, yield, and selected quality characteristics without using molecular markers is also a limitation of the study. The limited availability of seeds from gene banks meant that essential oils could only be obtained from genotypes sown in a single row. Therefore, similar to other characteristics, repeating the study by conducting experiments in a plot-based manner would be beneficial.

## Figures and Tables

**Table 1:** Detailed information on coriander genotypes used in the study.

No	Code	Genotype/Cultivar	Origin	No	Code	Genotype/Cultivar	Origin	No	Code	Genotype/Cultivar	Origin	No	Code	Genotype/Cultivar	Origin
1	GN-1	Ames 10234	Israel	41	GN-41	Ames 21655	Russia	81	GN-81	CN-19161	Canada	121	GN-121	Karamanlı	Türkiye
2	GN-2	Ames 10235	USA	42	GN-42	Ames 23614	Germany	82	GN-82	CN-19163	Canada	122	GN-122	Kerkük-f2	Türkiye
3	GN-3	Ames 13900	Tajikistan	43	GN-43	Ames 23616	Russia	83	GN-83	CN-19169	Canada	123	GN-123	Kırşehir	Türkiye
4	GN-4	Ames 14363	Egypt	44	GN-44	Ames 23618	Sudan	84	GN-84	CN-19172	Canada	124	GN-124	Kişniş Ankara	Türkiye
5	GN-5	Ames 1560	India	45	GN-45	Ames 23619	Bhutan	85	GN-85	CN-19176	Canada	125	GN-125	Kudret_K *	Türkiye
6	GN-6	Ames 158567	Morocco	46	GN-46	Ames 23620	Pakistan	86	GN-86	CN-19190	Canada	126	GN-126	Multan	Pakistan
7	GN-7	Ames 18559	Germany	47	GN-47	Ames 23621	Syria	87	GN-87	CN-30506	Canada	127	GN-127	Nazilli	Türkiye
8	GN-8	Ames 18563	France	48	GN-48	Ames 23622	Syria	88	GN-88	CN-30507	Canada	128	GN-128	Pelmus *	Türkiye
9	GN-9	Ames 18564	France	49	GN-49	Ames 23624	Syria	89	GN-89	CN-31472	Canada	129	GN-129	PI 170319	Türkiye
10	GN-10	Ames 18565	Germany	50	GN-50	Ames 23625	China	90	GN-90	CN-31475	Canada	130	GN-130	PI 170320	Türkiye
11	GN-11	Ames 18566	Czech Republic	51	GN-51	Ames 23627	Sudan	91	GN-91	CN-33962	Canada	131	GN-131	PI 171592	Türkiye
12	GN-12	Ames 18567	Spain	52	GN-52	Ames 23632	Oman	92	GN-92	CN-31477	Canada	132	GN-132	PI 172808	Türkiye
13	GN-13	Ames 18568	İtaly	53	GN-53	Ames 23634	Oman	93	GN-93	CN-37327	Canada	133	GN-133	PI 174129	Türkiye
14	GN-14	Ames 18569	Japan	54	GN-54	Ames 23635	Oman	94	GN-94	CN-37328	Canada	134	GN-134	PI 193769	Ethiopia
15	GN-15	Ames 18570	Japan	55	GN-55	Ames 23639	Oman	95	GN-95	CN-37329	Canada	135	GN-135	PI 193770	Ethiopia
16	GN-16	Ames 18571	Japan	56	GN-56	Ames 23641	Oman	96	GN-96	CN-37330	Canada	136	GN-136	PI 196843	Ethiopia
17	GN-17	Ames 18572	Netherlands	57	GN-57	Ames 23642	Pakistan	97	GN-97	CN-37331	Canada	137	GN-137	PI 249115	India
18	GN-18	Ames 18573	Netherlands	58	GN-58	Ames 24907	Bulgaria	98	GN-98	CN-37466	Canada	138	GN-138	PI 253146	Iran
19	GN-19	Ames 18574	Czech Republic	59	GN-59	Ames 24909	USA	99	GN-99	CN-37469	Canada	139	GN-139	PI 256061	Afghanistan
20	GN-20	Ames 18575	Russia	60	GN-60	Ames 24917	Portugal	100	GN-100	CN-37467	Canada	140	GN-140	PI 268378	Afghanistan
21	GN-21	Ames 18577	Russia	61	GN-61	Ames 24923	Georgia	101	GN-101	CN-37468	Canada	141	GN-141	PI 269470	Pakistan
22	GN-22	Ames 18578	Russia	62	GN-62	Ames 24926	Azerbaijan	102	GN-102	CN-72582	Canada	142	GN-142	PI 269472	Pakistan
23	GN-23	Ames 18580	Russia	63	GN-63	Ames 25170	USA	103	GN-103	Denizli-1	Türkiye	143	GN-143	PI 274290	India
24	GN-24	Ames 18581	Germany	64	GN-64	Ames 27392	Uzbekistan	104	GN-104	Erbaa *	Türkiye	144	GN-144	PI 478378	China
25	GN-25	Ames 18582	Denmark	65	GN-65	Ames 27770	Palestine	105	GN-105	Fındıklı-rize	Türkiye	145	GN-145	PI 483232	Chile
26	GN-26	Ames 18583	France	66	GN-66	Ames 27771	Palestine	106	GN-106	Gamze *	Türkiye	146	GN-146	PI 502320	Uzbekistan
27	GN-27	Ames 18584	Germany	67	GN-67	Ames 27772	Palestine	107	GN-107	Gürbüz *	Türkiye	147	GN-147	PI 531293	Hungary
28	GN-28	Ames 18585	Czech Republic	68	GN-68	Ames 27870	Palestine	108	GN-108	İ.T.P-1	Türkiye	148	GN-148	PI 531293	Hungary
29	GN-29	Ames 18588	Poland	69	GN-69	Ames 29172	Georgia	109	GN-109	İ.T.P-2	Türkiye	149	GN-149	PI 633685	Canada
30	GN-30	Ames 18589	Poland	70	GN-70	Ames 29173	Georgia	110	GN-110	İ.T.P-3	Türkiye	150	GN-150	PI 664512	Tajikistan
31	GN-31	Ames 18590	Poland	71	GN-71	Ames 29174	Georgia	111	GN-111	İ.T.P-4	Türkiye	151	GN-151	PI 664512	Tajikistan
32	GN-32	Ames 18591	Germany	72	GN-72	Ames 33640	Russia	112	GN-112	İ.T.P-5	Türkiye	152	GN-152	PI 669959	India
33	GN-33	Ames 18592	England	73	GN-73	Ames 4998	Türkiye	113	GN-113	İ.T.P-6	Türkiye	153	GN-153	PI 669960	India
34	GN-34	Ames 18594	England	74	GN-74	Ankara genotipi	Türkiye	114	GN-114	İslamabad-1	Pakistan	154	GN-154	PI 669961	India
35	GN-35	Ames 18596	Morocco	75	GN-75	Ankara-1	Türkiye	115	GN-115	İslamabad-2	Pakistan	155	GN-155	PI 669963	India
36	GN-36	Ames 19032	Kazakhstan	76	GN-76	Arslan *	Türkiye	116	GN-116	K.T.Erzurum-9	Türkiye	156	GN-156	PI 669964	India
37	GN-37	Ames 19089	Kazakhstan	77	GN-77	B.T. Mardin	Türkiye	117	GN-117	K.T.P-1	Türkiye	157	GN-157	PI 669965	India
38	GN-38	Ames 20046	Azerbaijan	78	GN-78	Burdur-2	Türkiye	118	GN-118	K.T.P-2	Türkiye	158	GN-158	PI 669965	India
39	GN-39	Ames 20048	Kazakhstan	79	GN-79	Burdur-İ. Şengül-1	Türkiye	119	GN-119	K.T.P-3	Türkiye	159	GN-159	PI 669966	India
40	GN-40	Ames 21108	India	80	GN-80	Burdur-İ. Şengül-2	Türkiye	120	GN-120	Karaman	Türkiye	160	GN-160	Sancarbey *	Türkiye

* Standard cultivars.

**Table 2:** UPOV criteria of the coriander genotypes of different origin.

No	Criteria	Score	Explanation	No	Criteria	Score	Explanation
1	Anthocyanin coloration in the flower	1	Absent	7	Number of leaflets in basal leaf	1	Three
		9	Present			2	Five
2	Intensity of anthocyanin coloration in the flowers	3	Weak	8	Size of terminal leaflet in leaf	3	Small
		5	Medium			5	Medium
		7	Strong			7	Large
3	Anthocyanin coloration of hypocotyl in seedling	1	Absent/very weak	9	Structure of feathering in basal leaf	1	Fine
		3	Weak			2	Medium
		5	Medium			3	Coarse
		7	Strong	10	Density of incisions on margin in leaflet	3	Sparse
		9	Very strong			5	Medium
4	Cotyledon shape	1	Narrow elliptic			7	Dense
		2	Elliptic	11	Fruit shape	1	Rounded
		3	Broad elliptic			2	Elongated
5		3	Sparse			3	Elliptic
	Density of foliage in plant	5	Medium	12	Intensity of brown color in fruit	3	Light
		7	Dense			5	Medium
6		1	Yellowish green				
	Coloration in foliage	3	Green				
		5	Dark green			7	Dark

**Table 3:** Phenological characteristics of the top three and bottom three coriander genotypes with the check cultivar.

Trait/Category	Parameter/Genotype	50% SD (Days)	Origin	50% FD (Days)	Origin	50% FSD (Days)	Origin
Top Genotypes	Top 1	GN-87 (18.95 j)	Canada	GN-52 (55.52 v–a)	Oman	GN-91 (61.67 lm)	Canada
	Top 2	GN-112 (21.95 i)	Türkiye	GN-142 (55.52 v–a)	Pakistan	GN-116 (64.10 klm)	Türkiye
	Top 3	GN-130 (21.95 i)	Türkiye	GN-151 (55.52 v–a)	Tajikistan	GN-22 (66.10 j–m)	Russia
Bottom Genotypes	Bottom 1	GN-21 (27.10 a)	Russia	GN-1 (74.67 a)	Israel	GN-1 (96.10 a)	Israel
	Bottom 2	GN-23 (27.10 a)	Russia	GN-14 (74.67 a)	Japan	GN-10 (96.10 a)	Germany
	Bottom 3	GN-42 (27.10 a)	Germany	GN-76 (74.67 a)	India	GN-135 (95.24 a)	Ethiopia
Standard Checks	Check Cultivar	GN-76 (23.00 g)	Türkiye	GN-107 (58.67 m–r)	Türkiye	GN-160 (69.00 h–m)	Türkiye

Differences between means indicated with the same letter are not significant. SD: seedling emergence days; FD: flowering days; FSD: fruit setting days. Full datasets for all 160 genotypes are provided in Supplementary Material Table S5.

**Table 4:** Plant height and branch number values of the top three and bottom three coriander genotypes with the check cultivar.

Trait/Category	Parameter/Genotype	PH	Origin	BN	Origin
Top Genotypes	Top 1	GN-87 (112.03 a)	Canada	GN-87 (10.24 a)	Canada
	Top 2	GN-132 (90.39 b)	Türkiye	GN-117 (9.04 ab)	Türkiye
	Top 3	GN-88 (89.63 bc)	Canada	GN-150 (8.79 abc)	Tajikistan
Bottom Genotypes	Bottom 1	GN-101 (38.73 z12)	Canada	GN-158 (1.18 q–l)	India
	Bottom 2	GN-143 (38.93 z12)	India	GN-118 (1.19 q–l)	Türkiye
	Bottom 3	GN-6 (39.03 y–z)	Morocco	GN-134 (1.29 l–j)	Ethiopia
Standard Checks	Check Cultivar	GN-125 (83.00 b–h)	Türkiye	GN-128 (5.77 g–y)	Türkiye

Differences between means indicated with the same letter are not significant; PH: plant height (cm); BN: branch number (per/plant). Full datasets for all 160 genotypes are provided in Supplementary Material Table S6.

**Table 5:** Number of umbels and umbellets values of the top three and bottom three coriander genotypes with the check cultivar.

Trait/Category	Parameter/Genotype	UN	Origin	UmbltN	Origin
Top Genotypes	Top 1	GN-80 (26.70 a)	Türkiye	GN-71 (117.60 a)	Georgia
	Top 2	GN-70 (21.10 b)	Georgia	GN-15 (81.20 b)	Japan
	Top 3	GN-38 (18.90 bc)	Azerbaijan	GN-3 (79.75 b)	Tajikistan
Bottom Genotypes	Bottom 1	GN-53 (2.79 yz)	Oman	GN-60 (4.30 s–z)	Portugal
	Bottom 2	GN-72 (3.19 vyz)	Russia	GN-136 (8.30 mn)	Ethiopia
	Bottom 3	GN-136 (3.20 vyz)	Ethiopia	GN-57 (8.40 mn)	Pakistan
Standard Checks	Check Cultivar	GN-128 (13.90 b–j)	Türkiye	GN-107 (76.97 n–m)	Türkiye

Differences between means indicated with the same letter are not significant; UN: umbel number, UmbltN: umbellet number. Full datasets for all 160 genotypes are provided in Supplementary Material Table S7.

**Table 6:** Yield and yield attribute values of the top three and bottom three coriander genotypes with the check cultivar.

**Trait/Category**	**Parameter/Genotype**	**1000 FW**	**Origin**	**FY**	**Origin**
Top Genotypes	Top 1	GN-146 (34.21 a–c)	Uzbekistan	GN-37 (4.98 a)	Kazakhstan
	Top 2	GN-33 (31.81 a–e)	England	GN-103 (3.82 b)	Türkiye
	Top 3	GN-65 (29.26 a–g)	Palestine	GN-33 (3.71 a–v)	England
Bottom Genotypes	Bottom 1	GN-103 (5.30 a–e)	Türkiye	GN-89 (0.05 z–l)	Canada
	Bottom 2	GN-100 (5.60 a–e)	Canada	GN-131 (0.06 z–l)	Türkiye
	Bottom 3	GN-118 (7.02 a–e)	Türkiye	GN-129 (0.12 z–l)	Türkiye
Standard Checks	Check Cultivar	GN-104 (28.32 a–e)	Türkiye	GN-104 (1.85 a–k)	Türkiye
**Trait/Category**	**Parameter/Genotype**	**BY**	**Origin**	**HI**	**Origin**
Top Genotypes	Top 1	GN-114 (49.24 a)	Pakistan	GN-1 (46.13 a)	Israel
	Top 2	GN-156 (16.08 b)	India	GN-38 (35.71 ab)	Azerbaijan
	Top 3	GN-144 (14.40 bc)	China	GN-130 (33.08 abc)	Türkiye
Bottom Genotypes	Bottom 1	GN-142 (0.97 op)	Pakistan	GN-11 (0.32 lm)	Czech Republic
	Bottom 2	GN-26 (1.04 nop)	France	GN-133 (0.59 lm)	Türkiye
	Bottom 3	GN-3 (1.21 m–p)	Tajikistan	GN-27 (0.67 lm)	Germany
Standard Checks	Check Cultivar	GN-128 (7.57 c–o)	Türkiye	GN-128 (8.83 g–l)	Türkiye

Differences between means indicated with the same letter are not significant; 1000 FW: 1000 fruit yield; FY: fruit yield; BY: biological yield; HI: harvest index. Full datasets for all 160 genotypes are provided in Supplementary Material Table S8.

**Table 7:** Essential oil content and composition values of the top three and bottom three coriander genotypes with the check cultivar.

**Trait/Category**	**Parameter/Genotype**	**EOC (%)**	**Origin**	**β-Pinene (%)**	**Origin**	**ɣ-Terpinene (%)**	**Origin**
Retention time				14.29		17.36	
Top Genotypes (HV)	Top 1	GN-154 (2.25 a)	İndia	GN-128 (23.73 a)	Türkiye	GN-106 (26.87 a)	Türkiye
	Top 2	GN-88 (1.65 b)	Canada	GN-106 (16.14 ab)	Türkiye	GN-107 (24.25 ab)	Türkiye
	Top 3	GN-118 (1.55 bc)	Türkiye	GN-107 (16.12 abc)	Türkiye	GN-128 (18.44 bc)	Türkiye
Bottom Genotypes (LV)	Bottom 1	GN-102 (0.02 uv)	Canada	GN-90 (0.04 h–k)	Canada	GN-34 (0.31 r–v)	England
	Bottom 2	GN-47 (0.06 tuv)	Syria	GN-53 (0.06 h–k)	Oman	GN-11 (0.37 q–v)	Czech Republic
	Bottom 3	GN-122 (0.10 s–v)	Türkiye	GN-34 (0.30 g–k)	England	GN-158 (0.41 q–v)	India
Standard Checks	Check Cultivar	GN-128 (0.75 j–k)	Türkiye	GN-104 (8.69 b–j)	Türkiye	GN-106 (7.08 e–s)	Türkiye
**Trait/Category**	**Parameter/Genotype**	**Linalool (%)**	**Origin**	**Camphor (%)**	**Origin**	***Trans*-geraniol (%)**	**Origin**
Retention time		21.7		24.07		26.31	
Top Genotypes	Top 1	GN-114 (48.90 a)	Pakistan	GN-19 (12.56 a)	Czech Republic	GN-19 (9.51 a)	Czech Republic
	Top 2	GN-73 (47.69 ab)	Türkiye	GN-120 (12.35 ab)	Türkiye	GN-133 (8.81 a–n)	Türkiye
	Top 3	GN-47 (44.33 abc)	Syria	GN-45 (11.43 abc)	Bhutan	GN-80 (7.75 ab)	Türkiye
Bottom Genotypes	Bottom 1	GN-2 (0.52 a)	Usa	GN-134 (0.03 12)	Ethiopia	GN-150 (0.05 vy)	Tajikistan
	Bottom 2	GN-3 (0.65 a)	Tajikistan	GN-46 (0.08 12)	Pakistan	GN-151 (0.05 vy)	Tajikistan
	Bottom 3	GN-103 (0.70 a–z)	Türkiye	GN-113 (0.10 12)	Türkiye	GN-34 (0.06 uvy)	England
Standard Checks	Check Cultivar	GN-106 (25.62 e–s)	Türkiye	GN-106 (7.98 c–y)	Türkiye	GN-106 (6.00 a–n)	Türkiye
**Trait/Category**	**Parameter/Genotype**	***Trans-*** **geranyl acetate (%)**	**Origin**	**13-tetradecenal (%)**	**Origin**	**Myristic acid (%)**	**Origin**
Retention time		28.27		30.27		30.64	
Top Genotypes	Top 1	GN-84 (24.90 a)	Canada	GN-31 (12.58 a)	Poland	GN-53 (23.61 a)	Oman
	Top 2	GN-152 (10.95 b)	India	GN-23 (10.51 ab)	Russia	GN-140 (19.10 ab)	Afghanistan
	Top 3	GN-47 (10.59 bc)	Syria	GN-121 (10.33 abc)	Türkiye	GN-134 (16.09 bc)	Ethiopia
Bottom Genotypes	Bottom 1	GN-9 (0.15 vy)	France	GN-53 (0.02 vy)	Oman	GN-46 (0.01 kl)	Pakistan
	Bottom 2	GN-34 (0.17 vy)	England	GN-72 (0.14 uvy)	Russia	GN-61 (0.01 kl)	Georgia
	Bottom 3	GN-103 (0.20 uvy)	Türkiye	GN-103 (0.14 uvy)	Türkiye	GN-73 (0.01 kl)	Türkiye
Standard Checks	Check Cultivar	GN-128 (8.00 b–l)	Türkiye	GN-160 (6.82 a–s)	Türkiye	GN-125 (6.32 g–z)	Türkiye

Differences between means indicated with the same letter are not significant. EOC: essential oil content. Full datasets for all 160 genotypes are provided in Supplementary Material Table S9.

**Table 8:** Correlation analysis of the phenological, morphological, yield, essential oil and essential oil compositions in coriander genotypes and cultivars.

Parameters	FD (Day)	FSD (Day)	PH (cm)	BN (Number)	UN (Number)	UmbltN (Number)	1000 FW (g)	FY (g/Plant)	BY (g/Plant)	HI (%)	EOC (%)	β-Pinene (%)	ɣ-Terpinene (%)	Linalool (%)	Camphor (%)	Trans-Geraniol (%)	Trans-Geranyl Acetate (%)	13-Tetradecenal (%)	Myristic Acid (%)
SD (day)	−0.027	0.103	0.02	0.097	−0.027	−0.067	−0.01	0.125	0.24 **	−0.017	−0.009	−0.175 *	−0.161 *	−0.052	−0.154	−0.052	−0.026	0.077	−0.001
FD (day)		0.318 **	0.197 *	0.001	0.166 *	0.09	0.108	0.096	−0.098	0.091	0.143	0.021	0.028	−0.017	−0.049	−0.028	−0.109	0.033	0.021
FSD (day)			0.101	0.083	0.147	0.202 *	0.164 *	0.108	0.146	0.234 **	0.131	0.082	0.176 *	−0.108	0.085	0.069	−0.109	−0.033	−0.065
PH (cm)				0.532 **	0.014	0.052	0.029	0.131	0.002	0.095	0.191 *	0.053	0.107	−0.007	0.032	−0.122	−0.146	−0.02	−0.124
BN (number)					0.048	−0.002	0.029	0.095	0.042	0.026	−0.024	0.014	0.051	−0.005	−0.086	−0.158 *	−0.16 *	−0.058	−0.07
UN (number)						0.504 **	−0.028	−0.082	0.193 *	0.244 **	0.159 *	0.134	0.126	−0.079	0.131	0.095	0.066	0.08	−0.048
UmbltN (number)							−0.09	−0.024	0.113	0.198 *	0.146	0.117	0.08	−0.124	0.125	0.038	0.168 *	−0.088	0.007
1000 FW (g)								0.09	−0.013	−0.067	−0.063	0.062	0.114	−0.006	−0.127	−0.108	−0.013	0.17 *	0.07
FY (g/plant)									0.099	0.016	−0.021	0.094	0.063	−0.153	−0.057	−0.162 *	−0.146	−0.104	0.014
BY (g/plant)										0.064	−0.063	0.032	−0.013	0.111	−0.021	−0.021	0.036	−0.011	−0.053
HI (%)											0.018	0.023	0.014	−0.023	−0.032	−0.1	−0.048	−0.11	−0.004
EOC (%)												0.04	0.132	−0.105	0.165 *	0.072	−0.114	0.093	0.021
β-pinene (%)													0.775 **	−0.022	0.37 **	0.202 *	0.141	−0.219 **	−0.202 *
ɣ-terpinene (%)														0.067	0.372 **	0.301 **	0.071	−0.045	−0.171 *
Linalool (%)															−0.031	0.03	0.077	0.079	0.308 **
Camphor (%)																0.743 **	0.337 **	−0.227 **	−0.255 **
Trans-geraniol (%)																	0.35 **	0.073	−0.133
Trans-geranyl acetate (%)																		0.047	−0.047
13-tetradecenal (%)																			0.039

*: significant at 0.05 level probability **: significant at 0.05 level probability; SD: seedling emergence day; FSD: fruit setting day; PH: plant height; UN: umbel number; UmbltN: umbellet number; FW: fruit weight; FY: fruit yield; BY: biological yield; HI: harvest index; EOC: essential oil content.

**Table 9:** Correlation analysis of the UPOV criteria and essential oil compositions in coriander genotypes and cultivars.

Parameters	PH	NBL	LD	GCP	TLS	DLS	LMS	PL	FS	FBCI	FSH	TBF	β-Pinene (%)	ɣ-Terpinene (%)	Linalool (%)	Camphor (%)	Trans-Geraniol (%)	Trans-Geranyl Acetate (%)	13-Tetradecenal (%)	Myristic Acid (%)
CS	0.194 *	−0.04	−0.064	−0.05	−0.045	−0.076	−0.025	−0.021	−0.146	0.063	0.069	0.111	0.141	0.068	0.025	0.13	0.166 *	0.094	−0.082	−0.072
PH		0.083	0.076	0.073	0.064	−0.006	−0.277 **	0.154	−0.028	0.085	−0.051	0.63 **	0.255 **	0.178 *	−0.073	0.261 **	0.148	0.049	−0.116	−0.251 **
NBL			0.796 **	0.601 **	0.746 **	0.262 **	−0.017	0.454 **	−0.087	0.054	−0.132	0.163 *	0.055	0.041	0.176 *	0.191 *	0.107	0.087	−0.068	0.042
LD				0.663 **	0.709 **	0.281 **	0.026	0.491 **	−0.032	0.025	−0.078	0.128	0.069	0.033	−0.011	0.258 **	0.131	0.174 *	−0.095	−0.027
GCP					0.546 **	0.14	−0.062	0.375 **	−0.024	0.067	−0.092	0.022	−0.042	0.033	0.098	0.204 **	0.092	0.124	−0.025	−0.053
TLS						0.449 **	0.039	0.46 **	−0.038	0.069	−0.177 *	0.063	−0.017	−0.026	0.067	0.153	0.066	0.076	−0.052	0.022
DLS							0.263 **	0.291 **	0.016	0 **	−0.196 *	−0.005	−0.189 *	−0.229 **	−0.059	0.044	0.019	0.064	−0.083	0.007
LMS								−0.124	−0.003	−0.138	0.065	−0.101	−0.179 *	−0.212 **	0.004	−0.088	−0.11	0.024	−0.074	0.025
PL									0.134	0.193 *	−0.197 *	0.246 **	0.077	−0.026	−0.047	0.272 **	0.132	0.1	−0.343 **	−0.021
FS										0.225 **	−0.101	−0.051	−0.067	−0.011	0.062	−0.117	−0.082	0.007	0.018	0.187 *
FBCI											−0.209 **	0.038	−0.001	0.029	0.035	0.094	0.052	−0.052	−0.187 *	0.081
FSH												−0.042	0.1	0.036	−0.055	−0.004	0.053	0.017	0.012	0.011
TBF													0.29 **	0.184 *	−0.026	0.312 **	0.163 *	0.115	−0.18 *	−0.255 **
β-Pinene (%)														0.775 **	−0.022	0.37 **	0.202 *	0.141	−0.219 **	−0.202 *
ɣ-terpinene (%)															0.067	0.372 **	0.301 **	0.071	−0.045	−0.171 *
Linalool (%)																−0.031	0.03	0.077	0.079	0.308 **
Camphor (%)																	0.743 **	0.337 **	−0.227 **	−0.255 **
Trans-geraniol (%)																		0.35 **	0.073	−0.133
Trans-geranyl acetate (%)																			0.047	−0.047
13-tetradecenal (%)																				0.039

*: significant at the 0.05 probability level; **: significant at the 0.05 probability level; CS: shape of cotyledon; PH: plant height; NBL: number of basal leaves; LD: leaf density; GCP: green color of plant; TLS: size of terminal leaf; DLS: density of leaf serration; PL: petiole length; FS: fruit size; FBCI: intensity of brown color in fruit; FSH: fruit shape; TBF: time of beginning of flowering.

## Data Availability

The data can be provided by the corresponding author.

## Supplementary Materials

The following supporting information can be downloaded at: https://www.mdpi.com/article/10.3390/biology15191787/s1, Table S1: Randomized field layout and plot sequence of the augmented randomized complete block design (ARCBD) generated using JMP software; Table S2: Climatic data for vegetation period and long-term (1929 to 2024); Table S3: ANOVA analysis results by comparison of repeated averages; Table S4: Descriptive statistics and 95% confidence intervals of morphological, yield and quality properties of the coriander genotypes; Table S5: Phenological characteristics of coriander genotypes; Table S6: Plant height and branch number values of coriander genotypes; Table S7: Number of umbel and umbellet of coriander genotypes; Table S8: Yield and yield attributes of coriander genotypes; Table S9: Essential oil content and major essential oil compositions of coriander genotypes; Table S10: Minor essential oil compositions of coriander genotypes; Table S11: UPOV criteria results of coriander genotypes.

## Abbreviations

The following abbreviations are used in this manuscript:

GC	Gas Chromatography
PH	Plant height
PCA	Principal Coordinate Analysis
EOC	Essential oil content
FY	Fruit yield
ED	Emergency day
FD	Flowering day
FSD	Fruit setting day
BY	Biological yield
HI	Harvest index
UN	Umbel number
