# Supplementary material for: Comprehensive Evaluation of Agromorphological Traits, Yield Potential, and Essential Oil Profiles in 160 Coriander (Coriandrum sativum L.) Genotypes
Source: Biology (Basel). 2026 Oct 8;15(19):1787. doi: 10.3390/biology15191787 (PMC13664577; doi:10.3390/biology15191787)
Supplement: Supplementary file 1 [file biology-15-01787-s001.zip › biology-4490713-supplementary.pdf]

Supplementary Tables

Table S1. Randomized field layout and plot sequence of the augmented randomized complete block design (ARCBD) generated using JMP software.

Block	No	Genotypes/ cultivars	Type	Random order	Block	No	Genotypes/ cultivars	Type	Random order	Block	No	Genotypes/ cultivars	Type	Random order
1	1	K.T.P-3-2013	Test genotype	0.122	2	61	Ames 33640	Test genotype	0.493	3	121	Ames 23641	Test genotype	0.648
1	2	CN-19163	Test genotype	0.690	2	62	PI 170320	Test genotype	0.239	3	122	CN-72581	Test genotype	0.844
1	3	Ames 23635	Test genotype	0.311	2	63	Ames 18589	Test genotype	0.298	3	123	PI 196843	Test genotype	0.806
1	4	PI 669965	Test genotype	0.421	2	64	Erbaa	Cultivar-2	0.860	3	124	PI 669965	Test genotype	0.833
1	5	PI 253146	Test genotype	0.853	2	65	Ames 23619	Test genotype	0.109	3	125	Ames 1560	Test genotype	0.782
1	6	CN-37327	Test genotype	0.218	2	66	CN-33962	Test genotype	0.018	3	126	Kudret K	Cultivar-6	0.254
1	7	PI 531293	Test genotype	0.583	2	67	Ames 18563	Test genotype	0.125	3	127	Ames 18596	Test genotype	0.839
1	8	Ames 27770	Test genotype	0.703	2	68	CN-19161	Test genotype	0.999	3	128	Ames 24909	Test genotype	0.213
1	9	Ames 23639	Test genotype	0.055	2	69	Ames 158561	Test genotype	0.578	3	129	Ames 25170	Test genotype	0.996
1	10	Ames 23620	Test genotype	0.264	2	70	Gürbüz	Cultivar-4	0.659	3	130	Pelmus	Cultivar-5	0.212
1	11	Ames 24923	Test genotype	0.342	2	71	Ames 18591	Test genotype	0.946	3	131	Ames 18569	Test genotype	0.110
1	12	Ames 27771	Test genotype	0.644	2	72	PI 669963	Test genotype	0.823	3	132	Ames 18570	Test genotype	0.083
1	13	Ames 18567	Test genotype	0.602	2	73	Sancarbey	Cultivar-7	0.178	3	133	PI 171592	Test genotype	0.484
1	14	Ames 4998	Test genotype	0.331	2	74	Ames 29172	Test genotype	0.149	3	134	Ames 19032	Test genotype	0.422
1	15	Nazilli	Test genotype	0.251	2	75	Ankara-1	Test genotype	0.132	3	135	Gürbüz	Cultivar-4	0.700
1	16	Ames 23625	Test genotype	0.400	2	76	CN-19176	Test genotype	0.441	3	136	PI 531293	Test genotype	0.376
1	17	PI 664512	Test genotype	0.195	2	77	PI 268378	Test genotype	0.868	3	137	Ames 29174	Test genotype	0.161
1	18	Ames 23621	Test genotype	0.538	2	78	Ames 20048	Test genotype	0.164	3	138	PI 174129	Test genotype	0.719
1	19	PI 193770	Test genotype	0.358	2	79	Ames 27772	Test genotype	0.433	3	139	Ames 18592	Test genotype	0.696
1	20	PI 669959	Test genotype	0.033	2	80	Karaman	Test genotype	0.472	3	140	Ames 29173	Test genotype	0.222
1	21	Ames 24926	Test genotype	0.752	2	81	CN-37467	Test genotype	0.664	3	141	Arslan	Cultivar-1	0.117
1	22	Gamze	Cultivar-3	0.865	2	82	Pelmus	Cultivar-5	0.651	3	142	PI 478378	Test genotype	0.293

1	23	Ames 18582	Test genotype	0.311	2	83	PI 669960	Test genotype	0.695	3	143	Ames 18566	Test genotype	0.510
1	24	PI 483232	Test genotype	0.455	2	84	CN-30506	Test genotype	0.416	3	144	PI 502320	Test genotype	0.990
1	25	Kudret-K	Cultivar-6	0.448	2	85	PI 669964	Test genotype	0.339	3	145	Ames 18565	Test genotype	0.059
1	26	PI 193769	Test genotype	0.638	2	86	PI 256061	Test genotype	0.642	3	146	Ames 27392	Test genotype	0.989
1	27	PI 269470	Test genotype	0.369	2	87	CN-19190	Test genotype	0.551	3	147	Ames 18584	Test genotype	0.582
1	28	Ames 18578	Test genotype	0.989	2	88	CN-37328	Test genotype	0.350	3	148	Ames 23622	Test genotype	0.683
1	29	Ames 23642	Test genotype	0.868	2	89	CN-31475	Test genotype	0.870	3	149	Erbaa	Cultivar-2	0.885
1	30	Ames 21655	Test genotype	0.841	2	90	PI 669961	Test genotype	0.185	3	150	PI 172808	Test genotype	0.439
1	31	Ames 23624	Test genotype	0.429	2	91	CN-30507	Test genotype	0.825	3	151	Ames 18590	Test genotype	0.026
1	32	Ames 10234	Test genotype	0.729	2	92	Burdur-2	Test genotype	0.847	3	152	Ames 18573	Test genotype	0.703
1	33	Arslan	Cultivar-1	0.125	2	93	CN-19169	Test genotype	0.929	3	153	Gamze	Cultivar-3	0.758
1	34	CN-37466	Test genotype	0.541	2	94	Arslan	Cultivar-1	0.443	3	154	CN-33961	Test genotype	0.037
1	35	CN-37330	Test genotype	0.339	2	95	PI 274290	Test genotype	0.715	3	155	Ames 23632	Test genotype	0.486
1	36	PI 170319	Test genotype	0.931	2	96	Ankara genotipi	Test genotype	0.709	3	156	Ames 18585	Test genotype	0.164
1	37	Ames 23616	Test genotype	0.810	2	97	Kerkük-F2	Test genotype	0.506	3	157	Ames 18571	Test genotype	0.546
1	38	Sancarbey	Cultivar-7	0.992	2	98	CN-31472	Test genotype	0.195	3	158	Ames 18588	Test genotype	0.706
1	39	CN-37469	Test genotype	0.845	2	99	Ames 13900	Test genotype	0.276	3	159	Ames 20046	Test genotype	0.721
1	40	Ames 18559	Test genotype	0.895	2	100	CN-37468	Test genotype	0.895	3	160	Sancarbey	Cultivar-7	0.884
1	41	K.T.P-2-2013	Test genotype	0.816	2	101	CN-72582	Test genotype	0.726	3	161	Ames 19089	Test genotype	0.020
1	42	Erbaa	Cultivar-2	0.316	2	102	Ames 10235	Test genotype	0.688	3	162	Ames 18564	Test genotype	0.169
1	43	Kırşehir	Test genotype	0.425	2	103	CN-19172	Test genotype	0.951	3	163	CN-37329	Test genotype	0.680
1	44	İ.T.P-1-2013	Test genotype	0.305	2	104	B.T. Mardin	Test genotype	0.867	3	164	Ames 24907	Test genotype	0.890
1	45	Ames 27870	Test genotype	0.009	2	105	Ames 18568	Test genotype	0.970	3	165	Ames 23614	Test genotype	0.728
1	46	Gürbüz	Cultivar-4	0.780	2	106	K.T.P-12013	Test genotype	0.212	3	166	Ames 18580	Test genotype	0.861
1	47	CN-37331	Test genotype	0.587	2	107	İ.T.P-3-2013	Test genotype	0.593	3	167	Kişniş Ankara	Test genotype	0.817
1	48	Ames 23627	Test genotype	0.626	2	108	Fındıklı-Rize	Test genotype	0.415	3	168	Karamanlı	Test genotype	0.527
1	49	İ.T.P-2-2013	Test genotype	0.391	2	109	K.T.Erzurum-97	Test genotype	0.139	3	169	Burdur-İ. şengül	Test genotype	0.504
1	50	Pelmus	Cultivar-5	0.743	2	110	Kudret-K	Cultivar-6	0.195	3	170	İslamabad	Test genotype	0.136
1	51	İ.T.P-4-2014	Test genotype	0.456	2	111	Ames 23634	Test genotype	0.659	3	171	PI 256146	Test genotype	0.630
1	52	İ.T.P-6-2013	Test genotype	0.348	2	112	Ames 18574	Test genotype	0.010	3	172	Ames 23618	Test genotype	0.971

1	53	PI 269472	Test genotype	0.958	2	113	Gamze	Cultivar-3	0.585	3	173	İslamabad	Test genotype	0.805
1	54	Ames 18572	Test genotype	0.379	2	114	Denizli-1	Test genotype	0.707	3	174	Denizli-2	Test genotype	0.327
1	55	PI 664512	Test genotype	0.156	2	115	Burdur-İ. şengül	Test genotype	0.281	3	175	PI 669966	Test genotype	0.925
1	56	PI 633685	Test genotype	0.891	2	116	İ.T.P-5-2013	Test genotype	0.506	3	176	Ames 18577	Test genotype	0.041
1	57	Ames 21108	Test genotype	0.960	2	117	CN-31477	Test genotype	0.806	3	177	PI 249115	Test genotype	0.189
1	58	Ames 18575	Test genotype	0.044	2	118	Ames 18583	Test genotype	0.745	3	178	Multan	Test genotype	0.252
1	59	Ames 24917	Test genotype	0.053	2	119	Ames 18581	Test genotype	0.459	3	179	Ames 18594	Test genotype	0.024
1	60	CN-19164	Test genotype	0.582	2	120	CN-36066	Test genotype	0.925	3	180	Ames 14363	Test genotype	0.602

Note: The experimental area was partitioned into 3 blocks. Check cultivars (Cultivars 1–7) were replicated across all blocks, whereas test genotypes were randomly assigned to plots within each block using the pseudo-random number generator algorithm in JMP.

Table S2. Climatic data for vegetation period and long-term (1929 to 2024).

Years	Parameter	March	April	May	June	July	August
2024	Mean temperature (°C)	7.1	14.3	13.4	21.9	22.1	21.7
	Total precipitation (mm)	31.6	20.7	77.1	6.7	63.2	0.1
	Mean relative humidity (%)	70.3	58.9	71.3	60.9	66.3	63.1
1929-2024	Mean temperature (°C)	4.7	9.7	14.1	17.4	19.8	20.0
	Total precipitation (mm)	50.7	50.5	60.6	59.3	28.7	24.4
	Mean relative humidity (%)	72.6	69.8	72.0	71.9	68.9	68.1

Tables S3. ANOVA analysis results by comparison of repeated averages.

	Source of Variation	DF	SS	MS	F	P	CV (%)	LSD (0.05)
**50% Seedling emergence day**	Block	2	0.10	0.05	0.46	0.64		0.52
	Cultivars	6	0.48	0.08	0.77	0.61		0.80*
	Error	12	1.24	0.10			1.39	
	Total	20	1.81					
	LSD (0.05) Test			Standard Error			LSD	
	Comparison of two non-replicated means in different blocks			0.52			1.12	
	Comparison of repeated (control) and non-repeated averages			0.43			0.91	
	Comparison of two non-replicated means in the same block			0.45			0.97	
	Comparison of repeated averages (control coriander cultivars)			0.26			0.56	
**50% Flowering day**	Source of Variation	DF	SS	MS	F	P	CV (%)	LSD (0.05)
	Block	2	4.95	2.48	4.66	0.03		1.19
	Cultivars	6	213.62	35.60	66.96	1.60		1.82*
	Error	12	6.38	0.53			1.16	
	Total	20	224.95					
	LSD (0.05) Test			Standard Error			LSD	
	Comparison of two non-replicated means in different blocks			1.19			2.54	
	Comparison of repeated (control) and non-repeated averages			0.97			2.07	
	Comparison of two non-replicated means in the same block			1.03			2.20	
	Comparison of repeated averages (control coriander cultivars)			0.60			1.27	
**50% Fruit setting day**	Source of Variation	DF	SS	MS	F	P	CV (%)	LSD (0.05)
	Block	2	69.24	34.62	1.33	0.30		8.34
	Cultivars	6	1312.95	218.83	8.40	0.00		12.73*
	Error	12	312.76	26.06			6.12	
	Total	20	1694.95					
	LSD (0.05) Test			Standard Error			LSD	
	Comparison of two non-replicated means in different blocks			8.34			17.77	
	Comparison of repeated (control) and non-repeated averages			6.81			14.51	

	Comparison of two non-replicated means in the same block			7.22			15.39	
	Comparison of repeated averages (control coriander cultivars)			4.17			8.89	
**Plant height**	Source of Variation	DF	SS	MS	F	P	CV (%)	LSD (0.05)
	Block	2	42.70	21.35	0.77	0.48		8.57
	Cultivars	6	2929.02	488.17	17.71	2.60		13.09*
	Error	12	330.82	27.57			8.32	
	Total	20	3302.54					
	LSD (0.05) Test			Standard Error			LSD	
	Comparison of two non-replicated means in different blocks			8.57			18.28	
	Comparison of repeated (control) and non-repeated averages			7.00			14.92	
	Comparison of two non-replicated means in the same block			7.43			15.83	
	Comparison of repeated averages (control coriander cultivars)			4.29			9.14	
**Branch number**	Source of Variation	DF	SS	MS	F	P	CV (%)	LSD (0.05)
	Block	2	0.60	0.30	0.43	0.66		1.37
	Cultivars	6	7.05	1.17	1.67	0.21		2.09*
	Error	12	8.44	0.70			16.70	
	Total	20	16.09					
	LSD (0.05) Test			Standard Error			LSD	
	Comparison of two non-replicated means in different blocks			1.37			2.92	
	Comparison of repeated (control) and non-repeated averages			1.12			2.38	
	Comparison of two non-replicated means in the same block			1.19			2.53	
	Comparison of repeated averages (control coriander cultivars)			0.68			1.46	
**Umbel number**	Source of Variation	DF	SS	MS	F	P	CV (%)	LSD (0.05)
	Block	2	3.07	1.53	0.23	0.79		4.18
	Cultivars	6	143.66	23.94	3.65	0.03		6.38*
	Error	12	78.61	6.55			30.06	
	Total	20	225.34					
	LSD (0.05) Test			Standard Error			LSD	
	Comparison of two non-replicated means in different blocks			4.18			8.91	
	Comparison of repeated (control) and non-repeated averages			3.41			7.28	

	Comparison of two non-replicated means in the same block			3.62			7.72	
	Comparison of repeated averages (control coriander cultivars)			2.09			4.46	
**Umbellet number**	Source of Variation	DF	SS	MS	F	P	CV (%)	LSD (0.05)
	Block	2	57.45	28.73	0.48	0.63		12.69
	Cultivars	6	1397.55	232.92	3.85	0.02		19.39*
	Error	12	725.21	60.43			23.92	
	Total	20	2180.21					
	LSD (0.05) Test			Standard Error			LSD	
	Comparison of two non-replicated means in different blocks			12.69			27.06	
	Comparison of repeated (control) and non-repeated averages			10.37			22.10	
	Comparison of two non-replicated means in the same block			10.99			23.44	
	Comparison of repeated averages (control coriander cultivars)			6.35			13.53	
**1000 Fruit weight**	Source of Variation	DF	SS	MS	F	P	CV (%)	LSD (0.05)
	Block	2	3.66	1.83	0.07	0.94		8.61
	Cultivars	6	444.21	74.04	2.66	0.07		13.15*
	Error	12	333.52	27.79			29.81	
	Total	20	781.38					
	LSD (0.05) Test			Standard Error			LSD	
	Comparison of two non-replicated means in different blocks			8.61			18.35	
	Comparison of repeated (control) and non-repeated averages			7.03			14.99	
	Comparison of two non-replicated means in the same block			7.46			15.89	
	Comparison of repeated averages (control coriander cultivars)			4.30			9.18	
**Fruit yield**	Source of Variation	DF	SS	MS	F	P	CV (%)	LSD (0.05)
	Block	2	3232.88	1616.44	0.23	0.80		136.76
	Cultivars	6	65018.73	10836.45	1.54	0.25		208.91*
	Error	12	84197.60	7016.47			61.71	
	Total	20	152449.21					
	LSD (0.05) Test			Standard Error			LSD	
	Comparison of two non-replicated means in different blocks			136.79			291.61	
	Comparison of repeated (control) and non-repeated averages			111.69			238.10	

	Comparison of two non-replicated means in the same block			118.46			252.54	
	Comparison of repeated averages (control coriander cultivars)			68.39			145.80	
**Biological yield**	Source of Variation	DF	SS	MS	F	P	CV (%)	LSD (0.05)
	Block	2	666.78	333.39	0.47	0.64		43.70
	Cultivars	6	2508.04	418.01	0.58	0.74		66.76*
	Error	12	8597.53	716.46			45.24	
	Total	20	11772.36					
	LSD (0.05) Test			Standard Error			LSD	
	Comparison of two non-replicated means in different blocks			43.71			93.18	
	Comparison of repeated (control) and non-repeated averages			35.69			76.08	
	Comparison of two non-replicated means in the same block			37.85			80.70	
	Comparison of repeated averages (control coriander cultivars)			21.85			46.59	
**Harvest index**	Source of Variation	DF	SS	MS	F	P	CV (%)	LSD (0.05)
	Block	2	18.02	9.01	0.40	0.68		7.76
	Cultivars	6	900.11	150.02	6.64	0.00		11.85*
	Error	12	270.97	22.58			27.33	
	Total	20	1189.09					
	LSD (0.05) Test			Standard Error			LSD	
	Comparison of two non-replicated means in different blocks			7.76			16.54	
	Comparison of repeated (control) and non-repeated averages			6.34			13.51	
	Comparison of two non-replicated means in the same block			6.72			14.33	
	Comparison of repeated averages (control coriander cultivars)			3.88			8.27	
**Essential oil content**	Source of Variation	DF	SS	MS	F	P	CV (%)	LSD (0.05)
	Block	2	0.02	0.01	0.33	0.73		0.28
	Cultivars	6	2.38	0.40	13.29	0.00		0.43*
	Error	12	0.36	0.03			23.25	
	Total	20	2.76					
	LSD (0.05) Test			Standard Error			LSD	
	Comparison of two non-replicated means in different blocks			0.28			0.60	
	Comparison of repeated (control) and non-repeated averages			0.23			0.49	

	Comparison of two non-replicated means in the same block			0.24			0.52	
	Comparison of repeated averages (control coriander cultivars)			0.14			0.30	
**Linalool**	Source of Variation	DF	SS	MS	F	P	CV (%)	LSD (0.05)
	Block	2	13.79	6.89	0.91	0.43		4.50
	Cultivars	6	96.51	16.09	2.11	0.13		6.88*
	Error	12	91.31	7.61			47.90	
	Total	20	201.61					
	LSD (0.05) Test			Standard Error			LSD	
	Comparison of two non-replicated means in different blocks			4.50			9.60	
	Comparison of repeated (control) and non-repeated averages			3.68			7.84	
	Comparison of two non-replicated means in the same block			3.90			8.32	
	Comparison of repeated averages (control coriander cultivars)			2.25			4.80	
**β-pinene**	Source of Variation	DF	SS	MS	F	P	CV (%)	LSD (0.05)
	Block	2	4.40	2.20	0.20	0.82		5.37
	Cultivars	6	116.16	19.36	1.79	0.18		8.20*
	Error	12	129.74	10.81			79.77	
	Total	20	250.30					
	LSD (0.05) Test			Standard Error			LSD	
	Comparison of two non-replicated means in different blocks			5.37			11.45	
	Comparison of repeated (control) and non-repeated averages			4.38			9.35	
	Comparison of two non-replicated means in the same block			4.65			9.91	
	Comparison of repeated averages (control coriander cultivars)			2.68			5.72	
**γ-terpinene**	Source of Variation	DF	SS	MS	F	P	CV (%)	LSD (0.05)
	Block	2	13.79	6.89	0.91	0.43		4.50
	Cultivars	6	96.51	16.09	2.11	0.13		6.88*
	Error	12	91.31	7.61			47.90	
	Total	20	201.61					
	LSD (0.05) Test			Standard Error			LSD	
	Comparison of two non-replicated means in different blocks			4.50			9.60	
	Comparison of repeated (control) and non-repeated averages			3.68			7.84	

	Comparison of two non-replicated means in the same block			3.90			8.32	
	Comparison of repeated averages (control coriander cultivars)			2.25			4.80	
**Camphor**	Source of Variation	DF	SS	MS	F	P	CV (%)	LSD (0.05)
	Block	2	14.52	7.26	1.90	0.19		3.19
	Cultivars	6	62.36	10.39	2.73	0.07		4.87*
	Error	12	45.76	3.81			36.62	
	Total	20	122.64					
	LSD (0.05) Test			Standard Error			LSD	
	Comparison of two non-replicated means in different blocks			3.19			6.80	
	Comparison of repeated (control) and non-repeated averages			2.60			5.55	
	Comparison of two non-replicated means in the same block			2.76			5.89	
	Comparison of repeated averages (control coriander cultivars)			1.59			3.40	
**trans-geraniol**	Source of Variation	DF	SS	MS	F	P	CV (%)	LSD (0.05)
	Block	2	2.33	1.17	0.19	0.83		4.01
	Cultivars	6	31.81	5.30	0.88	0.54		6.13*
	Error	12	72.47	6.04			54.65	
	Total	20	106.62					
	LSD (0.05) Test			Standard Error			LSD	
	Comparison of two non-replicated means in different blocks			4.01			8.56	
	Comparison of repeated (control) and non-repeated averages			3.28			6.99	
	Comparison of two non-replicated means in the same block			3.48			7.41	
	Comparison of repeated averages (control coriander cultivars)			2.01			4.28	
**trans-geranyl acetate**	Source of Variation	DF	SS	MS	F	P	CV (%)	LSD (0.05)
	Block	2	12.56	6.28	1.00	0.40		4.08
	Cultivars	6	36.53	6.09	0.97	0.48		6.24*
	Error	12	75.08	6.26			37.33	
	Total	20	124.17					
	LSD (0.05) Test			Standard Error			LSD	
	Comparison of two non-replicated means in different blocks			4.08			8.71	
	Comparison of repeated (control) and non-repeated averages			3.34			7.11	

	Comparison of two non-replicated means in the same block			3.54			7.54	
	Comparison of repeated averages (control coriander cultivars)			2.04			4.35	
**13-tetradecenal**	Source of Variation	DF	SS	MS	F	P	CV (%)	LSD (0.05)
	Block	2	6.12	3.06	0.36	0.70		4.75
	Cultivars	6	25.92	4.32	0.51	0.79		7.25*
	Error	12	101.41	8.45			55.28	
	Total	20	133.45					
	LSD (0.05) Test			Standard Error			LSD	
	Comparison of two non-replicated means in different blocks			4.75			10.12	
	Comparison of repeated (control) and non-repeated averages			3.88			8.26	
	Comparison of two non-replicated means in the same block			4.11			8.76	
	Comparison of repeated averages (control coriander cultivars)			2.37			5.06	
**Myristic acid**	Source of Variation	DF	SS	MS	F	P	CV (%)	LSD (0.05)
	Block	2	7.76	3.88	0.67	0.53		3.92
	Cultivars	6	55.06	9.18	1.59	0.23		5.99*
	Error	12	69.31	5.78			48.55	
	Total	20	132.13					
	LSD (0.05) Test			Standard Error			LSD	
	Comparison of two non-replicated means in different blocks			3.92			8.37	
	Comparison of repeated (control) and non-repeated averages			3.20			6.83	
	Comparison of two non-replicated means in the same block			3.40			7.25	
	Comparison of repeated averages (control coriander cultivars)			1.96			4.18	

DF: Degrees of freedom, SS: Sum of squares, MS: Mean square, CV (%): Coefficient of variation, P: Probability, LSD: Least significant difference ($p < 0.05$)

Table S4. Descriptive statistics and 95% confidence intervals of morphological, yield and quality properties of the coriander genotypes.

Properties	Mean ± sd	SEm	95% CI (Lower Limit)	95% CI (Upper Limit)	Min – Max	Range
SD (day)	23.41 ± 1.06	0.08	23.25	23.57	18.95 – 27.10	8.15
FD (day)	62.76 ± 4.70	0.36	62.06	63.47	55.52 – 74.67	19.15
FSD (day)	79.44 ± 9.93	0.76	77.95	80.93	61.67 – 96.10	34.43
PH (cm)	61.60 ± 13.63	1.04	59.56	63.64	38.73– 112.03	73.30
BN (number)	4.92 ± 1.37	0.10	4.72	5.12	2.19 – 10.24	8.05
UN (number)	8.15 ± 3.45	0.26	7.64	8.66	2.79 – 26.70	23.91
UmbltN (number)	32.01 ± 15.56	1.18	29.70	34.32	4.30 – 117.60	113.30
1000-FW (g)	17.55 ± 5.74	0.44	16.69	18.41	5.30 – 34.21	28.91
FY (g/plant)	1.22± 0.67	0.05	1.12	1.32	0.05 – 4.98	4.93
BY (g/plant)	5.44 ± 4.21	0.32	4.81	6.07	0.97 – 49.24	48.27
HI (%)	5.80 ± 6.15	0.47	4.88	6.72	0.32 – 46.13	45.81
EOC (%)	0.66 ± 0.34	0.03	0.61	0.71	0.02 – 2.25	2.23
β-pinene (%)	5.91 ± 4.20	0.32	5.28	6.54	0.04 – 23.73	23.69
γ-terpinene (%)	8.15 ± 4.96	0.35	7.40	8.89	0.31 – 26.87	26.56
Linalool (%)	24.44 ± 8.51	0.65	23.17	25.71	0.52 – 48.90	48.38
Camphor (%)	4.74 ± 2.54	0.19	4.35	5.12	0.03 – 9.64	9.61
Trans-geraniol (%)	4.57 ± 1.97	0.15	4.27	4.87	0.05 – 9.51	9.46
Trans-geranyl acetate (%)	6.43 ± 2.61	0.20	6.04	6.82	0.15 – 24.90	24.75
1,3-tetradecenal (%)	4.62 ± 2.42	0.18	4.25	4.98	0.02 – 12.58	12.56
Myristic acid (%)	4.59 ± 3.52	0.27	4.06	5.12	0.01 – 23.61	23.60

SD: Seedling emergence day, FD: Flowering day, FSD: Fruit setting day, PH: Plant height, BN: Branch number, UN: Umbel number, UmbltN: Umbellete number, FW: Fruit weight, FY: Fruit yield, BY: Biological Yield, HI: Harvest index, EOC: Essential oil content, sd: Standard deviation, SEm: Standard errors of mean, CI: Confidence interval (Lower Limit – Upper Limit), Min: Minimum, Max: Maximum.

Table S5. Phenological characteristics of coriander genotypes.

G/C	50% SD	G/C	50% SD	G/C	50% FD	G/C	50% FD	G/C	50% FSD	G/C	50% FSD
GN-1	22.95 gh	GN-81	23.95 ef	GN-1	74.67 a	GN-81	63.52 hı	GN-1	96.10 a	GN-81	66.10 j-m
GN-2	22.95 gh	GN-82	22.95 gh	GN-2	58.52 n-t	GN-82	63.52 hı	GN-2	82.24 a-h	GN-82	74.67 d-l
GN-3	22.95 gh	GN-83	24.95 cd	GN-3	64.67 gh	GN-83	63.52 hı	GN-3	87.67 a-d	GN-83	90.24 abc
GN-4	25.10 bc	GN-84	22.95 gh	GN-4	69.67 bcd	GN-84	63.52 hı	GN-4	87.67 a-d	GN-84	66.67 j-m
GN-5	23.10 fg	GN-85	22.95 gh	GN-5	57.81 n-u	GN-85	63.52 hı	GN-5	67.10 j-m	GN-85	87.67 a-d
GN-6	22.95 gh	GN-86	22.95 gh	GN-6	63.81 gh	GN-86	63.52 hı	GN-6	92.10 ab	GN-86	87.67 a-d
GN-7	22.95 gh	GN-87	18.95 j	GN-7	58.67 m-r	GN-87	63.33 hı	GN-7	66.67 j-m	GN-87	87.67 a-d
GN-8	22.95 gh	GN-88	22.95 gh	GN-8	58.52 n-t	GN-88	61.67 j	GN-8	82.24 a-h	GN-88	79.67 b-j
GN-9	23.10 fg	GN-89	22.95 gh	GN-9	71.67 b	GN-89	61.52 j-k	GN-9	79.67 b-j	GN-89	79.67 b-j
GN-10	23.10 fg	GN-90	24.95 cd	GN-10	70.81 bc	GN-90	60.81 jkl	GN-10	96.10 a	GN-90	87.67 a-d
GN-11	23.10 fg	GN-91	22.95 gh	GN-11	68.81 cde	GN-91	60.67 j-m	GN-11	79.10 b-j	GN-91	61.67 lm
GN-12	22.95 gh	GN-92	22.95 gh	GN-12	63.81 gh	GN-92	60.67 j-m	GN-12	92.10 ab	GN-92	89.67 abc
GN-13	25.95 b	GN-93	22.95 gh	GN-13	59.52 k-p	GN-93	60.67 j-m	GN-13	69.24 g-m	GN-93	79.67 b-j
GN-14	23.10 fg	GN-94	22.95 gh	GN-14	74.67 a	GN-94	60.67 j-m	GN-14	79.67 b-j	GN-94	69.24 g-m
GN-15	23.10 fg	GN-95	25.10 bc	GN-15	58.81 l-q	GN-95	59.81 j-n	GN-15	77.10 c-k	GN-95	79.67 b-j
GN-16	23.10 fg	GN-96	22.95 gh	GN-16	65.81 fg	GN-96	59.67 j-o	GN-16	92.10 ab	GN-96	84.10 a-f
GN-17	22.95 gh	GN-97	22.95 gh	GN-17	58.81 l-q	GN-97	59.52 k-p	GN-17	71.10 f-l	GN-97	90.24 abc
GN-18	23.10 fg	GN-98	22.95 gh	GN-18	68.52 de	GN-98	59.52 k-p	GN-18	70.24 f-m	GN-98	90.24 abc
GN-19	23.95 ef	GN-99	22.95 gh	GN-19	66.81 ef	GN-99	59.52 k-p	GN-19	92.10 ab	GN-99	82.24 a-h
GN-20	22.95 gh	GN-100	22.95 gh	GN-20	69.67 bcd	GN-100	59.52 k-p	GN-20	87.67 a-d	GN-100	69.24 g-m
GN-21	27.10 a	GN-101	22.95 gh	GN-21	63.52 hı	GN-101	59.52 k-p	GN-21	90.24 abc	GN-101	66.67 j-m
GN-22	22.95 gh	GN-102	22.95 gh	GN-22	57.81 n-u	GN-102	58.00 n-t	GN-22	66.10 j-m	GN-102	66.67 j-m
GN-23	27.10 a	GN-103	23.95 ef	GN-23	71.52 b	GN-103	58.81 l-q	GN-23	95.24 a	GN-103	79.67 b-j
GN-24	23.95 ef	GN-104	23.00 g	GN-24	68.81 cde	GN-104	58.81 l-q	GN-24	92.10 ab	GN-104	87.67 a-d
GN-25	22.95 gh	GN-105	22.95 gh	GN-25	69.67 bcd	GN-105	58.81 l-q	GN-25	81.67 a-ı	GN-105	83.67 a-g
GN-26	22.95 gh	GN-106	23.33 efg	GN-26	71.52 b	GN-106	58.81 l-q	GN-26	82.24 a-h	GN-106	89.67 abc

GN-27	23.10 fg	GN-107	23.00 g	GN-27	71.67 b	GN-107	58.67 m-r	GN-27	89.67 abc	GN-107	90.00 abc
GN-28	24.10 de	GN-108	22.95 gh	GN-28	68.81 cde	GN-108	58.67 m-r	GN-28	79.10 b-j	GN-108	87.33 a-d
GN-29	23.10 fg	GN-109	22.95 gh	GN-29	57.81 n-u	GN-109	68.52 de	GN-29	71.10 f-l	GN-109	82.24 a-h
GN-30	23.10 fg	GN-110	22.95 gh	GN-30	68.81 cde	GN-110	60.67 j-m	GN-30	92.10 ab	GN-110	94.24 a
GN-31	23.10 fg	GN-111	22.95 gh	GN-31	64.67 gh	GN-111	57.52 p-v	GN-31	87.67 a-d	GN-111	66.67 j-m
GN-32	23.10 fg	GN-112	21.95 ı	GN-32	68.81 cde	GN-112	58.67 m-r	GN-32	79.10 b-j	GN-112	69.24 g-m
GN-33	22.95 gh	GN-113	22.95 gh	GN-33	64.67 gh	GN-113	68.52 de	GN-33	87.67 a-d	GN-113	66.67 j-m
GN-34	25.10 bc	GN-114	27.10 a	GN-34	70.81 bc	GN-114	55.81 u-z	GN-34	92.10 ab	GN-114	90.24 abc
GN-35	23.10 fg	GN-115	25.10 bc	GN-35	57.81 n-u	GN-115	55.81 u-z	GN-35	67.10 j-m	GN-115	92.10 ab
GN-36	23.10 fg	GN-116	23.95 ef	GN-36	57.81 n-u	GN-116	69.67 bcd	GN-36	71.10 f-l	GN-116	64.10 klm
GN-37	23.10 fg	GN-117	22.95 gh	GN-37	68.81 cde	GN-117	64.67 gh	GN-37	92.10 ab	GN-117	87.67 a-d
GN-38	22.95 gh	GN-118	22.95 gh	GN-38	70.81 bc	GN-118	56.52 s-y	GN-38	83.10 a-h	GN-118	81.67 a-ı
GN-39	22.95 gh	GN-119	22.95 gh	GN-39	70.81 bc	GN-119	61.52 ıjk	GN-39	83.10 a-h	GN-119	69.24 g-m
GN-40	23.95 ef	GN-120	22.95 gh	GN-40	69.67 bcd	GN-120	56.67 r-y	GN-40	72.67 e-l	GN-120	90.24 abc
GN-41	24.95 cd	GN-121	27.10 a	GN-41	58.52 n-t	GN-121	63.81 gh	GN-41	69.24 g-m	GN-121	66.67 j-m
GN-42	27.10 a	GN-122	22.95 gh	GN-42	63.52 hı	GN-122	56.67 r-y	GN-42	82.24 a-h	GN-122	77.10 c-k
GN-43	22.95 gh	GN-123	22.95 gh	GN-43	59.81 j-n	GN-123	59.52 k-p	GN-43	71.10 f-l	GN-123	66.67 j-m
GN-44	25.10 bc	GN-124	27.10 a	GN-44	68.52 de	GN-124	56.81 q-v	GN-44	90.24 abc	GN-124	90.24 abc
GN-45	22.95 gh	GN-125	23.00 g	GN-45	57.81 n-u	GN-125	64.67 gh	GN-45	71.10 f-l	GN-125	71.10 f-l
GN-46	22.95 gh	GN-126	25.10 bc	GN-46	58.67 m-r	GN-126	57.81 n-u	GN-46	66.67 j-m	GN-126	89.67 abc
GN-47	22.95 gh	GN-127	22.95 gh	GN-47	56.52 s-y	GN-127	59.52 k-p	GN-47	69.24 g-m	GN-127	67.10 j-m
GN-48	23.10 fg	GN-128	23.00 g	GN-48	68.52 de	GN-128	67.67 def	GN-48	90.24 abc	GN-128	71.24 f-l
GN-49	22.95 gh	GN-129	22.95 gh	GN-49	58.81 l-q	GN-129	63.52 hı	GN-49	71.10 f-l	GN-129	90.00 abc
GN-50	22.95 gh	GN-130	21.95 ı	GN-50	59.52 k-p	GN-130	61.67 ıjl	GN-50	71.24 f-l	GN-130	90.24 abc
GN-51	22.95 gh	GN-131	23.10 fg	GN-51	63.52 hı	GN-131	60.81 jkl	GN-51	90.24 abc	GN-131	87.67 a-d
GN-52	23.10 fg	GN-132	23.10 fg	GN-52	55.52 v-a	GN-132	66.81 ef	GN-52	69.24 g-m	GN-132	77.10 c-k
GN-53	23.95 ef	GN-133	23.10 fg	GN-53	56.81 q-v	GN-133	68.81 cde	GN-53	71.10 f-l	GN-133	79.10 b-j
GN-54	22.95 gh	GN-134	22.95 gh	GN-54	57.67 o-u	GN-134	68.52 de	GN-54	66.67 j-m	GN-134	92.10 ab
GN-55	22.95 gh	GN-135	22.95 gh	GN-55	64.00 gh	GN-135	57.52 p-v	GN-55	69.24 g-m	GN-135	95.24 a
GN-56	22.95 gh	GN-136	23.10 fg	GN-56	64.67 gh	GN-136	63.81 gh	GN-56	82.24 a-h	GN-136	79.24 b-j

GN-57	22.95 gh	GN-137	24.10 de	GN-57	64.67 gh	GN-137	57.81 n-u	GN-57	66.67 j-m	GN-137	71.10 f-l
GN-58	24.10 de	GN-138	22.95 gh	GN-58	64.67 gh	GN-138	68.52 de	GN-58	82.24 a-h	GN-138	66.24 j-m
GN-59	23.10 fg	GN-139	22.95 gh	GN-59	64.67 gh	GN-139	60.67 j-m	GN-59	84.10 a-f	GN-139	87.67 a-d
GN-60	22.95 gh	GN-140	22.95 gh	GN-60	64.67 gh	GN-140	59.67 j-o	GN-60	79.10 b-j	GN-140	66.67 j-m
GN-61	22.95 gh	GN-141	22.95 gh	GN-61	64.67 gh	GN-141	57.52 p-v	GN-61	90.24 abc	GN-141	69.24 g-m
GN-62	22.95 gh	GN-142	23.95 ef	GN-62	64.67 gh	GN-142	55.52 v-a	GN-62	67.24 ı-m	GN-142	69.24 g-m
GN-63	23.10 fg	GN-143	24.95 cd	GN-63	64.67 gh	GN-143	64.67 gh	GN-63	69.24 g-m	GN-143	66.67 j-m
GN-64	23.10 fg	GN-144	23.10 fg	GN-64	64.67 gh	GN-144	58.81 l-q	GN-64	92.10 ab	GN-144	92.10 ab
GN-65	22.95 gh	GN-145	23.95 ef	GN-65	64.67 gh	GN-145	56.52 s-y	GN-65	92.10 ab	GN-145	69.24 g-m
GN-66	22.95 gh	GN-146	23.10 fg	GN-66	64.67 gh	GN-146	70.81 bc	GN-66	71.24 f-l	GN-146	92.10 ab
GN-67	22.95 gh	GN-147	23.95 ef	GN-67	64.52 gh	GN-147	68.81 cde	GN-67	69.24 g-m	GN-147	92.10 ab
GN-68	22.95 gh	GN-148	23.10 fg	GN-68	64.33 gh	GN-148	63.52 hı	GN-68	66.67 j-m	GN-148	90.24 abc
GN-69	22.95 gh	GN-149	23.95 ef	GN-69	63.81 gh	GN-149	57.52 p-v	GN-69	71.24 f-l	GN-149	67.24 ı-m
GN-70	25.10 bc	GN-150	24.95 cd	GN-70	63.81 gh	GN-150	63.52 hı	GN-70	87.67 a-d	GN-150	84.24 a-f
GN-71	23.10 fg	GN-151	23.95 ef	GN-71	63.81 gh	GN-151	55.52 v-a	GN-71	90.10 abc	GN-151	67.24 ı-m
GN-72	22.95 gh	GN-152	24.95 cd	GN-72	63.81 gh	GN-152	56.52 s-y	GN-72	92.10 ab	GN-152	69.24 g-m
GN-73	22.95 gh	GN-153	22.95 gh	GN-73	63.81 gh	GN-153	71.67 b	GN-73	66.67 j-m	GN-153	79.67 b-j
GN-74	22.95 gh	GN-154	22.95 gh	GN-74	63.81 gh	GN-154	74.67 a	GN-74	69.24 g-m	GN-154	79.67 b-j
GN-75	22.95 gh	GN-155	22.95 gh	GN-75	63.52 hı	GN-155	64.67 gh	GN-75	79.67 b-j	GN-155	87.67 a-d
GN-76	23.00 g	GN-156	22.95 gh	GN-76	63.52 hı	GN-156	56.67 r-y	GN-76	87.67 a-d	GN-156	66.67 j-m
GN-77	22.95 gh	GN-157	24.95 cd	GN-77	63.52 hı	GN-157	63.81 gh	GN-77	74.00 d-l	GN-157	86.10 a-e
GN-78	22.95 gh	GN-158	23.10 fg	GN-78	63.52 hı	GN-158	57.52 p-v	GN-78	66.67 j-m	GN-158	69.24 g-m
GN-79	27.10 a	GN-159	24.10 de	GN-79	63.52 hı	GN-159	57.81 n-u	GN-79	79.67 b-j	GN-159	71.10 f-l
GN-80	23.95 ef	GN-160	23.33 efg	GN-80	63.52 hı	GN-160	58.67 m-r	GN-80	87.67 a-d	GN-160	69.00 h-m

Differences between means indicated with the same letter are not significant. SD: Seedling days, FD: Flowering days, FSD: Fruit setting days.

Table S6. Plant height and branch number values of coriander genotypes.

G/C	PH	G/C	PH	G/C	BN	G/C	BN
GN-1	57.88 a-t	GN-81	70.23 e-y	GN-1	4.59 f-h	GN-81	2.61 l-j
GN-2	49.73 j-2	GN-82	52.28 f-1	GN-2	4.24 p-l	GN-82	3.39 y-j
GN-3	64.43 l-j	GN-83	45.13 p-2	GN-3	4.24 p-l	GN-83	4.34 n-l
GN-4	50.99 h-2	GN-84	55.73 y-u	GN-4	4.08 r-j	GN-84	4.04 r-j
GN-5	66.79 j-g	GN-85	66.63 f-t	GN-5	7.88 a-g	GN-85	4.54 f-j
GN-6	39.03 y-2	GN-86	52.83 e-1	GN-6	5.54 g-b	GN-86	1.84 j-h
GN-7	52.68 e-1	GN-87	112.03 a	GN-7	4.29 o-l	GN-87	10.24 a
GN-8	67.23 t-e	GN-88	89.63 bc	GN-8	4.44 m-l	GN-88	5.54 g-b
GN-9	76.59 b-p	GN-89	53.93 c-y	GN-9	5.28 f-e	GN-89	4.51 l-j
GN-10	70.39 e-y	GN-90	76.93 b-o	GN-10	3.68 t-j	GN-90	6.74 b-m
GN-11	72.69 e-t	GN-91	64.93 k-l	GN-11	4.98 j-t	GN-91	3.64 t-j
GN-12	47.28 m-2	GN-92	78.13 b-m	GN-12	3.29 a-j	GN-92	4.94 j-g
GN-13	68.23 h-d	GN-93	44.58 q-2	GN-13	4.54 f-j	GN-93	3.49 v-j
GN-14	43.79 s-2	GN-94	63.63 m-k	GN-14	3.48 v-j	GN-94	4.51 l-j
GN-15	65.79 j-h	GN-95	76.79 b-p	GN-15	4.28 o-l	GN-95	5.08 j-e
GN-16	50.39 t-2	GN-96	71.08 e-v	GN-16	3.08 c-j	GN-96	6.59 c-p
GN-17	48.58 -12	GN-97	59.18 r-y	GN-17	4.39 m-l	GN-97	2.19 j
GN-18	57.49 u-t	GN-98	68.88 f-b	GN-18	4.28 o-l	GN-98	5.29 f-e
GN-19	63.73 l-k	GN-99	46.38 o-2	GN-19	4.14 q-l	GN-99	4.39 m-l
GN-20	55.08 z-u	GN-100	46.53 n-2	GN-20	4.79 j-h	GN-100	3.84 s-j
GN-21	47.79 m-2	GN-101	38.73 l2	GN-21	4.58 f-h	GN-101	5.04 j-e
GN-22	77.48 b-n	GN-102	69.13 l-a	GN-22	5.39 h-c	GN-102	3.74 t-j
GN-23	72.79 e-t	GN-103	64.63 k-j	GN-23	5.68 g-z	GN-103	3.44 y-j
GN-24	62.03 o-m	GN-104	66.17 j-g	GN-24	3.94 s-j	GN-104	5.70 g-z
GN-25	63.18 n-l	GN-105	70.33 e-y	GN-25	4.59 f-h	GN-105	5.34 h-d
GN-26	68.13 h-d	GN-106	66.53 l-f	GN-26	3.74 t-j	GN-106	4.83 j-h
GN-27	68.79 f-c	GN-107	56.57 v-t	GN-27	4.38 m-l	GN-107	5.27 f-e
GN-28	49.39 k-2	GN-108	44.28 r-2	GN-28	5.88 g-u	GN-108	3.49 v-j
GN-29	68.89 f-b	GN-109	44.38 r-2	GN-29	7.68 b-h	GN-109	3.49 v-j
GN-30	83.23 b-g	GN-110	41.13 s-2	GN-30	6.64 c-o	GN-110	5.24 f-e
GN-31	72.29 e-u	GN-111	49.48 k-2	GN-31	5.28 f-e	GN-111	4.29 o-l
GN-32	58.63 s-z	GN-112	43.83 s-2	GN-32	5.14 j-e	GN-112	2.94 d-j
GN-33	77.59 b-n	GN-113	48.48 -12	GN-33	4.68 j-h	GN-113	4.19 q-l
GN-34	83.49 b-f	GN-114	69.79 f-z	GN-34	8.28 a-f	GN-114	5.98 f-t
GN-35	50.89 h-2	GN-115	41.59 u-2	GN-35	3.78 t-j	GN-115	3.08 c-j
GN-36	61.99 p-m	GN-116	72.83 e-s	GN-36	3.58 u-j	GN-116	5.94 f-u
GN-37	63.39 m-l	GN-117	49.33 k-2	GN-37	4.28 o-l	GN-117	9.04 ab
GN-38	72.39 e-u	GN-118	54.28 a-v	GN-38	4.28 o-l	GN-118	1.19 q-l
GN-39	68.43 g-d	GN-119	59.48 r-q	GN-39	3.74 t-j	GN-119	4.69 j-h
GN-40	72.68 e-t	GN-120	46.73 n-2	GN-40	5.39 h-c	GN-120	3.34 z-j
GN-41	87.78 bcd	GN-121	69.99 e-z	GN-41	5.69 g-z	GN-121	4.98 j-f
GN-42	47.49 m-2	GN-122	45.13 p-2	GN-42	5.88 g-u	GN-122	4.34 n-l
GN-43	75.38 c-q	GN-123	67.88 l-d	GN-43	3.89 s-j	GN-123	4.19 q-l

GN-44	46.39 o-2	GN-124	77.19 b-n	GN-44	3.18 b-j	GN-124	7.88 a-g
GN-45	39.83 v-2	GN-125	83.00 b-h	GN-45	4.14 q-l	GN-125	5.10 j-e
GN-46	47.18 u-2	GN-126	66.19 j-g	GN-46	3.59 u-j	GN-126	3.28 a-j
GN-47	46.58 n-2	GN-127	52.58 e-1	GN-47	4.99 j-f	GN-127	3.69 t-j
GN-48	52.39 e-1	GN-128	72.43 e-t	GN-48	3.48 v-j	GN-128	5.77 g-y
GN-49	74.88 c-q	GN-129	66.81 f-t	GN-49	7.59 b-i	GN-129	4.19 q-l
GN-50	47.68 m-2	GN-130	79.93 b-i	GN-50	3.69 t-j	GN-130	6.64 c-o
GN-51	47.78 m-2	GN-131	50.49 z-2	GN-51	2.59 g-j	GN-131	3.68 t-j
GN-52	53.69 d-z	GN-132	90.39 b	GN-52	2.48 h-j	GN-132	8.28 a-f
GN-53	48.63 l-2	GN-133	75.29 c-q	GN-53	4.84 j-h	GN-133	5.28 f-e
GN-54	54.08 b-v	GN-134	51.48 g-1	GN-54	3.69 t-j	GN-134	1.29 l-j
GN-55	55.18 z-u	GN-135	52.88 e-1	GN-55	2.99 d-j	GN-135	4.19 q-l
GN-56	69.59 f-z	GN-136	68.39 g-d	GN-56	4.98 j-t	GN-136	4.28 o-l
GN-57	49.48 k-2	GN-137	79.89 b-j	GN-57	3.99 s-j	GN-137	6.98 b-k
GN-58	79.39 h-k	GN-138	61.38 q-n	GN-58	4.58 f-h	GN-138	5.09 j-e
GN-59	89.19 bc	GN-139	54.13 b-v	GN-59	6.38 d-r	GN-139	4.64 k-h
GN-60	55.48 y-u	GN-140	50.93 h-2	GN-60	5.59 g-a	GN-140	4.74 j-h
GN-61	69.98 e-z	GN-141	78.18 b-m	GN-61	5.59 g-a	GN-141	8.69 a-d
GN-62	71.38 e-v	GN-142	49.28 k-2	GN-62	4.59 f-h	GN-142	8.49 a-e
GN-63	76.69 b-p	GN-143	38.93 z-12	GN-63	4.38 m-l	GN-143	5.34 h-d
GN-64	77.39 b-n	GN-144	73.89 d-r	GN-64	6.48 c-q	GN-144	5.48 h-b
GN-65	44.38 r-2	GN-145	73.18 d-s	GN-65	5.39 h-c	GN-145	4.79 j-h
GN-66	51.48 g-1	GN-146	76.59 b-p	GN-66	3.79 t-j	GN-146	4.68 j-h
GN-67	48.53 t-2	GN-147	68.78 f-c	GN-67	3.74 t-j	GN-147	6.89 b-l
GN-68	44.38 r-2	GN-148	66.39 j-g	GN-68	3.39 y-j	GN-148	6.18 e-s
GN-69	80.23 b-j	GN-149	68.88 f-b	GN-69	5.84 g-v	GN-149	6.89 b-l
GN-70	72.29 e-u	GN-150	78.58 b-l	GN-70	6.68 b-u	GN-150	8.79 abc
GN-71	63.89 l-k	GN-151	51.18 h-1	GN-71	5.58 g-a	GN-151	4.39 m-l
GN-72	57.33 v-t	GN-152	51.18 h-1	GN-72	5.04 j-e	GN-152	4.49 m-l
GN-73	64.78 k-l	GN-153	61.13 q-o	GN-73	4.89 j-g	GN-153	4.54 f-j
GN-74	84.83 b-e	GN-154	80.13 b-j	GN-74	7.04 b-j	GN-154	4.24 p-l
GN-75	66.73 f-t	GN-155	59.63 r-p	GN-75	3.74 t-j	GN-155	4.04 r-j
GN-76	50.23 t-2	GN-156	39.63 v-2	GN-76	4.37 m-l	GN-156	4.64 k-h
GN-77	49.13 k-2	GN-157	81.39 b-i	GN-77	3.94 s-j	GN-157	4.79 j-h
GN-78	73.93 d-r	GN-158	41.88 p-2	GN-78	5.44 h-c	GN-158	1.18 q-l
GN-79	77.03 b-n	GN-159	43.69 t-2	GN-79	5.84 g-v	GN-159	3.18 b-j
GN-80	69.59 f-z	GN-160	46.86 n-2	GN-80	6.58 c-p	GN-160	4.13 q-j

Differences between means indicated with the same letter are not significant, PH: Plant height (cm), BN: Branch number (per/plant).

Table S7. Number of umbel and umbellets of coriander genotypes.

G/C	UN	G/C	UN	G/C	UmbltN	G/C	UmbltN
GN-1	11.51 d-s	GN-81	5.19 o-z	GN-1	36.55 f-l	GN-81	28.80 k-m
GN-2	6.29 n-y	GN-82	7.11 j-y	GN-2	31.05 j-l	GN-82	22.95 s-n
GN-3	14.39 b-h	GN-83	7.09 j-y	GN-3	79.75 b	GN-83	32.35 j-k
GN-4	11.50 d-s	GN-84	9.39 g-y	GN-4	32.40 j-k	GN-84	35.45 f-g
GN-5	4.90 o-z	GN-85	10.29 e-v	GN-5	15.10 t-n	GN-85	79.75 b
GN-6	13.09 c-n	GN-86	13.59 c-m	GN-6	65.35 b-g	GN-86	27.05 n-m
GN-7	7.91 h-y	GN-87	10.69 e-u	GN-7	24.45 o-m	GN-87	62.65 b-h
GN-8	4.39 r-z	GN-88	7.89 h-y	GN-8	19.05 a-n	GN-88	26.55 n-m
GN-9	6.70 j-y	GN-89	8.79 g-y	GN-9	16.80 c-n	GN-89	34.85 f-l
GN-10	13.80 c-k	GN-90	4.89 o-z	GN-10	71.90 bcd	GN-90	46.15 g-o
GN-11	5.00 o-z	GN-91	8.99 g-y	GN-11	20.60 v-n	GN-91	16.45 d-n
GN-12	5.51 o-z	GN-92	6.29 n-y	GN-12	20.45 v-n	GN-92	35.15 f-l
GN-13	5.79 o-z	GN-93	11.71 c-q	GN-13	26.65 n-m	GN-93	38.45 f-d
GN-14	7.60 h-y	GN-94	7.39 h-y	GN-14	32.20 j-k	GN-94	33.55 j-k
GN-15	11.60 d-r	GN-95	7.80 h-y	GN-15	81.20 b	GN-95	28.05 l-m
GN-16	8.00 g-y	GN-96	10.21 e-v	GN-16	36.70 f-n	GN-96	31.20 j-l
GN-17	5.41 o-z	GN-97	7.21 h-y	GN-17	26.65 n-m	GN-97	45.15 g-q
GN-18	8.80 g-y	GN-98	9.91 f-y	GN-18	27.60 m-m	GN-98	19.85 z-n
GN-19	9.59 g-y	GN-99	17.21 b-e	GN-19	36.35 f-f	GN-99	77.15 bc
GN-20	6.41 m-y	GN-100	5.99 n-z	GN-20	22.05 u-n	GN-100	41.35 h-z
GN-21	6.60 k-y	GN-101	16.99 b-f	GN-21	25.10 o-m	GN-101	35.85 f-f
GN-22	6.01 n-z	GN-102	5.99 n-z	GN-22	24.35 o-m	GN-102	45.05 g-r
GN-23	6.30 n-y	GN-103	3.69 u-z	GN-23	23.20 q-n	GN-103	40.05 f-a
GN-24	9.89 f-y	GN-104	9.20 g-y	GN-24	56.25 c-l	GN-104	13.15 h-n
GN-25	8.91 g-y	GN-105	8.89 g-y	GN-25	39.15 l-b	GN-105	34.80 j-l
GN-26	4.39 r-z	GN-106	8.33 g-y	GN-26	18.55 a-n	GN-106	34.25 j-l
GN-27	18.20 bcd	GN-107	6.90 j-y	GN-27	40.50 l-a	GN-107	76.97 n-m
GN-28	7.20 h-y	GN-108	6.51 l-y	GN-28	28.00 l-m	GN-108	20.35 y-n
GN-29	6.40 m-y	GN-109	11.61 d-r	GN-29	25.70 o-m	GN-109	40.65 h-a
GN-30	5.49 o-z	GN-110	8.09 g-y	GN-30	24.75 o-m	GN-110	38.75 f-e
GN-31	7.30 h-y	GN-111	9.71 g-y	GN-31	19.80 z-n	GN-111	39.05 f-b
GN-32	9.09 g-y	GN-112	4.49 q-z	GN-32	34.15 j-j	GN-112	18.95 a-n
GN-33	6.80 j-y	GN-113	8.61 g-y	GN-33	23.50 p-m	GN-113	30.65 k-l
GN-34	7.30 h-y	GN-114	7.40 h-y	GN-34	32.70 j-k	GN-114	29.90 k-m
GN-35	3.60 u-z	GN-115	8.40 g-y	GN-35	13.50 g-n	GN-115	36.70 f-f
GN-36	5.30 o-z	GN-116	9.99 e-y	GN-36	15.50 l-n	GN-116	36.50 f-l
GN-37	7.00 j-y	GN-117	9.39 g-y	GN-37	21.30 v-n	GN-117	45.45 g-p
GN-38	18.90 bc	GN-118	5.91 n-z	GN-38	42.50 h-v	GN-118	26.50 n-m
GN-39	5.49 o-z	GN-119	6.71 j-y	GN-39	22.75 s-n	GN-119	26.15 o-m
GN-40	10.61 e-u	GN-120	3.59 u-z	GN-40	44.15 g-t	GN-120	17.15 b-n
GN-41	8.01 g-y	GN-121	6.20 n-y	GN-41	32.75 j-k	GN-121	20.60 v-n
GN-42	12.10 c-o	GN-122	5.69 o-z	GN-42	39.60 l-a	GN-122	22.25 s-n
GN-43	6.91 j-y	GN-123	7.81 h-y	GN-43	29.55 k-m	GN-123	39.05 f-b

GN-44	6.00 n-z	GN-124	8.80 g-y	GN-44	21.30 v-n	GN-124	31.30 j-l
GN-45	5.09 o-z	GN-125	9.60 g-y	GN-45	25.75 o-m	GN-125	40.60 h-a
GN-46	9.71 g-y	GN-126	8.20 g-y	GN-46	33.35 j-k	GN-126	28.40 k-m
GN-47	5.81 o-z	GN-127	4.91 o-z	GN-47	16.35 e-n	GN-127	15.35 f-n
GN-48	7.50 h-y	GN-128	13.90 b-j	GN-48	16.70 c-n	GN-128	43.93 g-u
GN-49	8.11 g-y	GN-129	6.11 n-y	GN-49	23.25 q-m	GN-129	21.25 v-n
GN-50	8.51 g-y	GN-130	11.29 d-l	GN-50	41.95 h-y	GN-130	41.85 h-z
GN-51	7.51 h-y	GN-131	5.80 o-z	GN-51	32.45 j-k	GN-131	23.20 q-n
GN-52	15.20 b-g	GN-132	7.10 j-y	GN-52	71.80 b-e	GN-132	23.00 r-n
GN-53	2.79 yz	GN-133	5.30 o-z	GN-53	9.75 lmn	GN-133	20.30 y-n
GN-54	5.91 n-z	GN-134	11.11 d-l	GN-54	11.70 k-n	GN-134	53.05 d-j
GN-55	8.71 g-y	GN-135	7.31 h-y	GN-55	21.55 v-n	GN-135	29.75 k-m
GN-56	4.10 t-z	GN-136	3.20 v-yz	GN-56	31.15 j-l	GN-136	8.30 mn
GN-57	5.41 o-z	GN-137	4.80 p-z	GN-57	8.40 mn	GN-137	13.40 g-n
GN-58	10.10 e-v	GN-138	5.51 o-z	GN-58	23.75 p-m	GN-138	22.05 u-n
GN-59	4.30 s-z	GN-139	6.89 j-y	GN-59	30.70 k-l	GN-139	30.55 k-l
GN-60	4.11 t-z	GN-140	5.79 o-z	GN-60	4.30 s-z	GN-140	27.85 l-m
GN-61	8.11 g-y	GN-141	10.61 e-u	GN-61	12.75 j-n	GN-141	44.45 g-s
GN-62	7.71 h-y	GN-142	3.81 u-z	GN-62	45.45 g-p	GN-142	13.15 f-n
GN-63	8.20 g-y	GN-143	4.79 p-z	GN-63	30.75 k-l	GN-143	20.35 y-n
GN-64	5.50 o-z	GN-144	9.00 g-y	GN-64	45.10 g-q	GN-144	39.00 f-b
GN-65	6.11 n-z	GN-145	8.21 g-y	GN-65	21.50 v-n	GN-145	22.75 s-n
GN-66	10.51 e-u	GN-146	9.20 g-y	GN-66	30.35 k-m	GN-146	39.90 f-a
GN-67	6.79 j-y	GN-147	9.60 g-y	GN-67	44.25 g-t	GN-147	48.50 f-n
GN-68	7.11 j-y	GN-148	6.70 j-y	GN-68	32.85 j-k	GN-148	25.05 o-m
GN-69	8.39 g-y	GN-149	7.01 j-y	GN-69	35.25 i-h	GN-149	27.45 m
GN-70	21.10 b	GN-150	5.71 o-z	GN-70	34.05 j	GN-150	23.35 q-m
GN-71	9.80 f-y	GN-151	5.61 o-z	GN-71	117.60 a	GN-151	19.85 z-n
GN-72	3.19 v-z	GN-152	9.31 g-y	GN-72	29.20 k-m	GN-152	41.65 h-z
GN-73	7.91 h-y	GN-153	13.69 c-l	GN-73	11.85 k-n	GN-153	50.25 d-k
GN-74	6.49 l-y	GN-154	6.79 j-y	GN-74	38.05 u-e	GN-154	36.15 f-f
GN-75	6.59 k-y	GN-155	11.79 c-p	GN-75	23.85 p-m	GN-155	49.25 f-m
GN-76	6.20 n-y	GN-156	14.29 b-l	GN-76	31.85 j-k	GN-156	44.05 g-u
GN-77	4.29 s-z	GN-157	8.21 g-y	GN-77	24.67 o-m	GN-157	34.25 j-l
GN-78	8.99 g-y	GN-158	7.00 j-y	GN-78	20.35 y-n	GN-158	21.40 v-n
GN-79	5.29 o-z	GN-159	13.90 b-j	GN-79	49.75 e-l	GN-159	70.30 b-f
GN-80	26.70 a	GN-160	5.48 o-z	GN-80	25.05 o-m	GN-160	20.00 y-n

Differences between means indicated with the same letter are not significant, UN: Umbel number, UmbltN: Umbellet number.

Table S8. Yield and yield attributes of coriander genotypes.

G/C	1000 FW	G/C	1000 FW	G/C	FY	G/C	FY	G/C	BY	G/C	BY	G/C	HI	G/C	HI
GN-1	17.51 a	GN-81	11.23 a-e	GN-1	1.24 b	GN-81	1.08 a-k	GN-1	3.91 h-o	GN-81	4.38 g-o	GN-1	46.13 a	GN-81	6.98 h-l
GN-2	18.42 a	GN-82	26.96 ab	GN-2	1.24 b	GN-82	0.14 z-l	GN-2	3.61 h-p	GN-82	3.67 h-p	GN-2	5.14 h-l	GN-82	2.25 lm
GN-3	19.74 a-e	GN-83	22.48 a-e	GN-3	1.37 ab	GN-83	0.32 e-l	GN-3	1.21 m-p	GN-83	3.84 h-p	GN-3	12.61 e-l	GN-83	8.45 g-l
GN-4	12.56 a-e	GN-84	9.11 a-e	GN-4	1.76 a-x	GN-84	0.82 p-l	GN-4	8.65 b-n	GN-84	10.37 b-l	GN-4	2.68 klm	GN-84	3.54 kl
GN-5	18.94 a-e	GN-85	11.20 a-e	GN-5	1.76 a-x	GN-85	2.13 a-s	GN-5	4.56 f-o	GN-85	6.10 e-o	GN-5	1.12 lm	GN-85	21.79 c-g
GN-6	18.96 a-e	GN-86	11.29 a-e	GN-6	1.70 b-j	GN-86	0.32 e-l	GN-6	11.78 b-g	GN-86	10.70 b-h	GN-6	2.56 lm	GN-86	4.75 h-l
GN-7	16.48 a-e	GN-87	18.24 a-e	GN-7	0.27 z-l	GN-87	0.79 e-l	GN-7	7.11 c-o	GN-87	4.99 f-o	GN-7	7.05 h-l	GN-87	9.72 f-l
GN-8	18.28 a-e	GN-88	11.56 a-e	GN-8	1.06 a-k	GN-88	0.95 e-l	GN-8	2.29 k-p	GN-88	2.04 ı-p	GN-8	6.45 h-l	GN-88	1.32 lm
GN-9	8.79 a-e	GN-89	7.55 a-e	GN-9	0.33 e-l	GN-89	0.05 z-l	GN-9	3.98 h-o	GN-89	3.01 ı-p	GN-9	1.15 lm	GN-89	2.23 lm
GN-10	18.81 a-e	GN-90	18.48 a-e	GN-10	1.35 a-c	GN-90	0.42 p-l	GN-10	9.92 b-j	GN-90	1.76 ı-p	GN-10	16.10 d-k	GN-90	3.94 jkl
GN-11	17.41 a-e	GN-91	15.41 a-e	GN-11	1.89 a-k	GN-91	0.33 e-l	GN-11	6.24 e-o	GN-91	12.16 b-f	GN-11	0.32 lm	GN-91	2.43 lm
GN-12	21.71 a-c	GN-92	23.64 a-e	GN-12	0.76 e-l	GN-92	0.52 i-l	GN-12	4.12 h-o	GN-92	5.59 e-o	GN-12	0.72 lm	GN-92	8.93 g-l
GN-13	15.35 a-e	GN-93	11.89 a-e	GN-13	0.56 q-l	GN-93	0.21 p-l	GN-13	2.50 j-p	GN-93	4.10 h-o	GN-13	3.73 jkl	GN-93	8.07 h-l
GN-14	26.84 ab	GN-94	10.03 a-e	GN-14	3.09 ab	GN-94	0.74 e-l	GN-14	6.78 d-o	GN-94	2.88 ı-p	GN-14	2.87 klm	GN-94	3.24 kl
GN-15	27.01 a-e	GN-95	18.89 a-e	GN-15	1.25 b-h	GN-95	0.69 i-l	GN-15	8.02 c-o	GN-95	8.69 b-m	GN-15	1.49 lm	GN-95	6.15 h-l
GN-16	19.01 a-e	GN-96	19.21 a-e	GN-16	0.24 z-l	GN-96	1.96 a-x	GN-16	7.91 c-o	GN-96	3.30 h-p	GN-16	4.47 h-l	GN-96	3.19 kl
GN-17	27.04 a-e	GN-97	11.26 a-e	GN-17	0.58 i-l	GN-97	0.95 e-l	GN-17	7.07 c-o	GN-97	3.92 h-o	GN-17	6.70 h-l	GN-97	3.96 jkl
GN-18	20.15 a-e	GN-98	17.20 a-e	GN-18	1.26 b-h	GN-98	0.63 e-l	GN-18	5.79 e-o	GN-98	7.65 c-o	GN-18	3.43 kl	GN-98	9.59 f-l
GN-19	11.89 a-e	GN-99	8.59 a-e	GN-19	1.56 a-f	GN-99	0.29 e-l	GN-19	5.41 f-o	GN-99	4.41 g-o	GN-19	1.46 lm	GN-99	4.39 ı-l
GN-20	21.26 a-e	GN-100	5.60 a-e	GN-20	0.96 e-l	GN-100	0.29 e-l	GN-20	3.85 h-o	GN-100	3.35 h-p	GN-20	3.21 kl	GN-100	3.04 kl
GN-21	17.53 a-e	GN-101	17.89 a-e	GN-21	1.75 a-x	GN-101	0.27 z-l	GN-21	3.35 h-p	GN-101	6.01 e-o	GN-21	7.77 h-l	GN-101	2.25 lm
GN-22	19.94 a-e	GN-102	22.71 a-e	GN-22	1.58 a-e	GN-102	1.13 a-i	GN-22	5.12 f-o	GN-102	2.29 k-p	GN-22	2.06 lm	GN-102	5.81 h-l
GN-23	21.26 a-e	GN-103	5.30 a-e	GN-23	1.37 a-c	GN-103	3.82 a	GN-23	4.13 h-o	GN-103	2.08 ı-p	GN-23	5.26 h-l	GN-103	2.27 lm
GN-24	19.24 a-e	GN-104	28.32 a-e	GN-24	1.00 a-k	GN-104	1.85 a-k	GN-24	7.12 c-o	GN-104	5.55 e-o	GN-24	2.56 lm	GN-104	0.79 lm
GN-25	19.71 a-e	GN-105	22.56 a-e	GN-25	1.47 a-d	GN-105	0.33 e-l	GN-25	4.10 h-o	GN-105	4.15 h-o	GN-25	9.71 f-l	GN-105	3.07 kl
GN-26	15.98 a-e	GN-106	24.88 a-g	GN-26	1.95 a-x	GN-106	1.65 a-e	GN-26	1.04 nop	GN-106	5.21 f-o	GN-26	3.99 jkl	GN-106	3.32 kl
GN-27	13.08 a-e	GN-107	17.81 a-e	GN-27	1.16 a-i	GN-107	1.82 a-k	GN-27	7.10 c-o	GN-107	7.53 c-o	GN-27	0.67 lm	GN-107	6.71 h-l

GN-28	18.18 a-e	GN-108	15.15 a-e	GN-28	1.18 a-h	GN-108	0.54 i-l	GN-28	4.22 g-o	GN-108	6.78 d-o	GN-28	4.30 ı-l	GN-108	5.67 h-l
GN-29	11.35 a-e	GN-109	21.98 a-e	GN-29	1.13 a-i	GN-109	0.54 i-l	GN-29	5.07 f-o	GN-109	5.60 e-o	GN-29	2.62 klm	GN-109	4.08 ı-l
GN-30	11.34 a-e	GN-110	11.89 a-e	GN-30	1.16 a-i	GN-110	1.75 a-x	GN-30	4.16 h-o	GN-110	3.36 h-p	GN-30	4.39 ı-l	GN-110	4.13 ı-l
GN-31	27.09 a-e	GN-111	17.75 a-e	GN-31	1.70 b-k	GN-111	0.62 e-l	GN-31	5.40 f-o	GN-111	4.61 f-o	GN-31	2.69 klm	GN-111	1.64 lm
GN-32	15.34 a-e	GN-112	11.23 a-e	GN-32	1.53 a-e	GN-112	1.35 a-c	GN-32	4.95 f-o	GN-112	2.79 ı-p	GN-32	6.94 h-l	GN-112	2.61 klm
GN-33	31.81 a-e	GN-113	21.36 a-e	GN-33	3.71 a-v	GN-113	1.30 a-d	GN-33	5.46 e-o	GN-113	5.32 f-o	GN-33	2.41 lm	GN-113	1.71 lm
GN-34	11.45 a-e	GN-114	17.24 a-e	GN-34	1.93 a-x	GN-114	2.22 a-s	GN-34	4.43 g-o	GN-114	49.24 a	GN-34	8.38 g-l	GN-114	3.96 jkl
GN-35	16.04 a-e	GN-115	17.24 a-e	GN-35	1.28 a-g	GN-115	1.23 b-h	GN-35	1.31 m-p	GN-115	7.68 c-o	GN-35	2.24 lm	GN-115	26.24 bcd
GN-36	12.12 a-e	GN-116	16.94 a-e	GN-36	1.89 a-k	GN-116	0.19 z-l	GN-36	4.29 g-o	GN-116	4.73 f-o	GN-36	12.13 e-l	GN-116	5.63 h-l
GN-37	16.14 a-e	GN-117	19.16 a-e	GN-37	4.98 a	GN-117	1.56 a-f	GN-37	3.89 h-o	GN-117	1.71 m-p	GN-37	3.35 kl	GN-117	5.12 h-l
GN-38	15.04 a-e	GN-118	7.02 a-e	GN-38	1.14 a-i	GN-118	1.15 a-h	GN-38	7.13 c-o	GN-118	5.83 e-o	GN-38	35.71 ab	GN-118	1.07 lm
GN-39	21.16 a-e	GN-119	20.23 a-e	GN-39	1.47 a-d	GN-119	1.09 a-k	GN-39	4.17 g-o	GN-119	5.13 f-o	GN-39	7.06 h-l	GN-119	4.03 jkl
GN-40	21.21 a-e	GN-120	28.21 a-e	GN-40	1.87 a-k	GN-120	0.96 e-l	GN-40	3.85 h-o	GN-120	2.34 j-p	GN-40	3.43 kl	GN-120	3.81 jkl
GN-41	17.96 a-e	GN-121	29.21 a-e	GN-41	1.35 a-c	GN-121	1.70 b-k	GN-41	2.94 ı-p	GN-121	4.22 g-o	GN-41	4.27 ı-l	GN-121	3.14 kl
GN-42	17.96 a-e	GN-122	19.35 a-e	GN-42	1.35 a-c	GN-122	1.56 a-f	GN-42	9.74 b-k	GN-122	3.57 h-p	GN-42	7.81 h-l	GN-122	17.20 d-j
GN-43	20.96 a-e	GN-123	15.44 a-e	GN-43	1.59 a-e	GN-123	1.56 a-f	GN-43	2.12 ı-p	GN-123	7.18 c-o	GN-43	1.15 lm	GN-123	2.60 klm
GN-44	17.29 a-e	GN-124	19.89 a-e	GN-44	1.00 a-k	GN-124	1.29 a-h	GN-44	3.31 h-p	GN-124	7.22 c-o	GN-44	6.22 h-l	GN-124	6.35 h-l
GN-45	9.89 a-e	GN-125	17.51 a-e	GN-45	1.10 a-k	GN-125	0.62 e-l	GN-45	7.28 c-o	GN-125	5.57 e-o	GN-45	1.55 lm	GN-125	3.87 jkl
GN-46	16.81 a-e	GN-126	11.20 a-e	GN-46	1.58 a-e	GN-126	1.18 a-h	GN-46	7.98 c-o	GN-126	13.04 b-e	GN-46	2.75 klm	GN-126	23.06 b-f
GN-47	19.65 a-e	GN-127	27.08 a-e	GN-47	1.14 a-i	GN-127	1.20 a-h	GN-47	3.80 h-p	GN-127	9.33 b-l	GN-47	6.57 h-l	GN-127	2.88 kl
GN-48	19.78 a-e	GN-128	19.05 a-e	GN-48	1.09 a-k	GN-128	1.70 b-k	GN-48	4.77 f-o	GN-128	7.57 c-o	GN-48	3.16 kl	GN-128	8.83 g-l
GN-49	10.55 a-e	GN-129	21.21 a-e	GN-49	0.50 e-l	GN-129	0.12 z-l	GN-49	6.10 e-o	GN-129	5.13 f-o	GN-49	5.67 h-l	GN-129	9.11 g-l
GN-50	10.88 a-e	GN-130	16.94 a-e	GN-50	1.05 a-k	GN-130	1.21 a-h	GN-50	4.11 h-o	GN-130	5.13 f-o	GN-50	4.00 jkl	GN-130	33.08 abc
GN-51	10.65 a-e	GN-131	17.11 a-e	GN-51	0.20 z-l	GN-131	0.06 z-l	GN-51	3.47 h-p	GN-131	4.23 g-o	GN-51	4.19 ı-l	GN-131	5.06 h-l
GN-52	11.63 g-a	GN-132	18.15 a-e	GN-52	1.05 a-k	GN-132	1.37 a-b	GN-52	4.02 h-o	GN-132	4.92 f-o	GN-52	17.54 d-l	GN-132	24.13 b-e
GN-53	9.10 g-a	GN-133	10.11 a-e	GN-53	1.49 a-d	GN-133	0.53 e-l	GN-53	2.07 ı-p	GN-133	3.79 h-p	GN-53	5.42 h-l	GN-133	0.59 lm
GN-54	9.89 g-a	GN-134	19.18 a-e	GN-54	0.58 i-l	GN-134	0.56 q-l	GN-54	2.99 ı-p	GN-134	5.53 e-o	GN-54	1.45 lm	GN-134	4.89 h-l
GN-55	12.35 a-e	GN-135	28.74 a-g	GN-55	1.09 a-k	GN-135	0.76 e-l	GN-55	6.35 d-o	GN-135	3.31 h-p	GN-55	5.18 h-l	GN-135	5.76 h-l
GN-56	19.28 a-e	GN-136	14.23 a-e	GN-56	0.94 e-l	GN-136	1.25 b-h	GN-56	2.77 ı-p	GN-136	3.08 ı-p	GN-56	8.75 g-l	GN-136	2.47 lm
GN-57	14.44 a-e	GN-137	16.24 a-e	GN-57	1.14 a-i	GN-137	2.38 a-p	GN-57	2.57 j-p	GN-137	3.09 ı-p	GN-57	1.99 lm	GN-137	1.64 lm

GN-58	9.09 a-e	GN-138	15.95 a-e	GN-58	0.52 i-l	GN-138	0.76 e-l	GN-58	4.06 h-o	GN-138	6.52 d-o	GN-58	4.59 h-l	GN-138	6.22 h-l
GN-59	14.04 a-e	GN-139	15.96 a-e	GN-59	1.14 a-i	GN-139	2.52 b-e	GN-59	3.31 h-p	GN-139	4.61 f-o	GN-59	8.90 g-l	GN-139	2.70 klm
GN-60	21.34 a-e	GN-140	20.81 a-e	GN-60	1.15 a-h	GN-140	1.67 a-e	GN-60	2.31 k-p	GN-140	6.60 d-o	GN-60	1.80 lm	GN-140	4.98 h-l
GN-61	16.25 a-e	GN-141	22.21 a-e	GN-61	1.00 a-k	GN-141	0.96 e-l	GN-61	8.55 b-o	GN-141	4.56 f-o	GN-61	4.38 ı-l	GN-141	4.28 ı-l
GN-62	27.86 a-d	GN-142	15.20 a-e	GN-62	0.78 e-l	GN-142	0.96 e-l	GN-62	5.35 f-o	GN-142	0.97 op	GN-62	4.32 ı-l	GN-142	9.07 g-l
GN-63	20.65 a-d	GN-143	14.61 a-e	GN-63	1.58 a-e	GN-143	1.18 a-h	GN-63	3.92 h-o	GN-143	5.34 f-o	GN-63	2.88 kl	GN-143	3.13 kl
GN-64	21.29 a-e	GN-144	8.09 a-e	GN-64	1.24 b-h	GN-144	1.52 b-h	GN-64	13.94 bcd	GN-144	14.40 bc	GN-64	5.03 h-l	GN-144	17.93 d-h
GN-65	29.26 a-g	GN-145	15.71 a-e	GN-65	0.20 z-l	GN-145	1.09 a-k	GN-65	5.30 f-o	GN-145	1.80 ı-p	GN-65	4.30 ı-l	GN-145	1.87 lm
GN-66	27.25 a-e	GN-146	34.21 a-c	GN-66	1.20 a-h	GN-146	1.12 a-i	GN-66	4.06 h-o	GN-146	5.72 e-o	GN-66	3.59 kl	GN-146	3.95 jkl
GN-67	11.23 a-e	GN-147	10.89 a-e	GN-67	1.54 a-e	GN-147	1.27 a-g	GN-67	5.00 f-o	GN-147	7.61 c-o	GN-67	2.08 lm	GN-147	2.90 kl
GN-68	12.65 a-e	GN-148	25.71 a-e	GN-68	1.58 a-e	GN-148	1.65 a-e	GN-68	5.94 e-o	GN-148	4.54 f-o	GN-68	10.66 e-l	GN-148	10.32 f-l
GN-69	19.76 a-e	GN-149	11.71 a-e	GN-69	1.58 a-e	GN-149	1.60 a-e	GN-69	5.34 f-o	GN-149	6.90 c-o	GN-69	6.84 h-l	GN-149	2.93 kl
GN-70	19.18 a-e	GN-150	14.86 a-e	GN-70	1.98 a-x	GN-150	1.49 a-d	GN-70	6.21 e-o	GN-150	4.70 f-o	GN-70	2.61 klm	GN-150	7.05 h-l
GN-71	12.65 a-e	GN-151	25.58 a-e	GN-71	1.33 a-c	GN-151	0.76 e-l	GN-71	7.93 c-o	GN-151	3.39 h-p	GN-71	3.61 kl	GN-151	3.75 jkl
GN-72	19.21 a-c	GN-152	11.26 a-e	GN-72	1.06 a-k	GN-152	0.95 e-l	GN-72	3.37 h-p	GN-152	3.28 h-p	GN-72	1.57 lm	GN-152	5.05 h-l
GN-73	11.76 a-e	GN-153	15.20 a-e	GN-73	1.09 a-k	GN-153	0.20 z-l	GN-73	4.21 g-o	GN-153	2.74 j-p	GN-73	6.07 h-l	GN-153	1.15 lm
GN-74	12.76 a-e	GN-154	17.02 a-e	GN-74	1.23 b-h	GN-154	1.87 a-k	GN-74	1.32 m-p	GN-154	1.56 m-p	GN-74	3.86 jkl	GN-154	11.65 e-l
GN-75	21.28 a-e	GN-155	19.86 a-e	GN-75	1.25 b-h	GN-155	0.84 a-l	GN-75	7.47 c-o	GN-155	5.97 e-o	GN-75	1.32 lm	GN-155	3.89 jkl
GN-76	24.81 a-g	GN-156	9.39 a-e	GN-76	1.73 a-x	GN-156	1.53 a-e	GN-76	4.47 g-o	GN-156	16.08 b	GN-76	5.50 h-l	GN-156	8.46 g-l
GN-77	23.02 a-g	GN-157	19.26 a-e	GN-77	0.70 i-l	GN-157	1.56 a-f	GN-77	3.04 ı-p	GN-157	5.07 f-o	GN-77	2.34 lm	GN-157	9.35 g-l
GN-78	8.98 a-e	GN-158	21.54 a-e	GN-78	0.50 e-l	GN-158	1.75 a-x	GN-78	1.91 ı-p	GN-158	4.70 f-o	GN-78	4.64 h-l	GN-158	2.78 klm
GN-79	13.74 a-e	GN-159	11.89 a-e	GN-79	1.33 a-c	GN-159	0.76 e-l	GN-79	7.38 c-o	GN-159	4.15 h-o	GN-79	2.86 klm	GN-159	11.38 e-l
GN-80	24.12 a-e	GN-160	14.25 a-e	GN-80	1.38 a-b	GN-160	1.26 b-h	GN-80	8.17 c-o	GN-160	5.51 e-o	GN-80	8.34 g-l	GN-160	5.08 h-l

Differences between means indicated with the same letter are not significant, 1000 FW: 1000 fruit yield, FY: Fruit yield, BY: Biological yield, HI: Harvest index.

Table S9. Essential oil content and major essential oil compositions of coriander genotypes.

G/C	EOC (%)	β-Pinene (%)	ɣ-terpinene (%)	Linalool (%)	Camphor (%)	*Trans*-geraniol (%)	*Trans*-geranyl *acetat* (%)	13-tetradecanal (%)	Myristic acid (%)
GN-1	0.58 t-s	5.32 e-j	9.74 e-n	29.40 c-r	5.72 k-q	4.77 a-v	7.63 b-l	5.21 b-v	7.58 f-p
GN-2	0.56 t-s	0.64 jk	1.07 tuv	0.52 a	1.03 t-2	0.61 n-y	2.77 y	0.16 uvy	0.72 d-l
GN-3	0.95 d-z	4.54 e-j	8.09 e-r	0.65 a	8.31 a-u	5.57 a-s	7.91 b-l	1.66 l-y	5.58 h-k
GN-4	0.60 r-s	6.34 d-j	10.59 d-l	21.84 ı-v	9.02 a-r	6.83 a-g	5.55 b-y	2.72 e-y	0.37 g-l
GN-5	0.32 g-v	7.43 b-j	8.53 e-p	22.04 ı-v	7.74 a-c	5.14 a-v	7.04 b-o	4.28 b-v	0.28 ı-l
GN-6	1.23 b-k	5.50 d-j	8.48 e-p	21.81 ı-v	8.20 b-v	5.57 a-s	7.31 b-m	2.73 e-y	5.53 h-l
GN-7	0.52 v-t	4.89 e-j	7.19 e-r	23.74 f-u	6.49 f-1	3.63 b-y	6.15 b-r	3.63 d-v	3.35 l-k
GN-8	0.49 y-t	7.16 b-j	8.74 e-o	20.41 j-y	3.14 d-2	4.88 a-v	7.21 b-n	8.59 a-h	0.98 a-l
GN-9	0.28 j-v	1.47 f-k	3.22 k-v	41.05 a-g	1.31 r-2	1.97 d-y	0.15 vy	7.48 a-o	2.22 q-k
GN-10	0.89 f-b	6.81 b-j	11.23 c-l	19.90 j-y	7.86 c-z	3.58 b-y	6.43 b-q	4.37 b-v	0.84 c-l
GN-11	0.42 b-v	0.69 f-k	0.37 q-v	31.40 a-r	1.98 n-2	0.08 uvy	6.65 b-o	2.61 e-y	5.70 h-e
GN-12	0.48 y-t	3.65 e-k	9.46 e-n	17.78 k-a	5.28 n-t	5.62 a-s	8.04 b-l	7.70 a-m	4.84 ı-k
GN-13	0.25 m-v	3.15 f-k	3.51 v-v	22.87 h-u	4.76 r-v	6.14 a-l	6.65 b-o	4.06 c-v	4.85 ı-k
GN-14	0.46 z-v	6.49 d-j	10.24 d-m	21.65 ı-v	7.55 c-e	4.62 a-v	7.67 b-l	4.83 b-v	4.74 ı-k
GN-15	0.77 ı-j	7.81 b-j	9.51 e-n	25.74 e-s	7.54 c-e	4.49 a-v	8.16 b-l	4.21 b-v	0.99 a-l
GN-16	1.25 b-ı	5.49 d-j	7.23 e-r	17.65 k-a	4.42 t-z	4.51 a-v	5.00 d-y	6.77 a-s	5.78 h-e
GN-17	0.18 q-v	4.34 e-j	5.38 f-v	33.10 a-o	0.11 -12	0.16 t-y	4.13 f-y	4.46 b-v	9.24 e-j
GN-18	0.70 n-o	0.76 f-k	2.11 n-v	33.79 a-m	4.42 t-z	5.24 a-v	5.95 b-s	7.44 a-o	2.47 o-k
GN-19	0.64 r-q	2.59 f-k	7.86 e-r	27.07 c-s	12.56 a	9.51 a	6.97 b-o	1.67 l-y	0.94 a-l
GN-20	0.55 t-t	2.44 f-k	4.09 g-v	30.28 b-r	0.46 y-2	4.02 b-y	1.10 p-y	2.47 f-y	5.21 h-k
GN-21	0.66 r-q	3.24 e-k	5.74 f-u	30.73 b-r	1.56 p-2	1.78 e-y	8.21 b-l	2.04 j-y	4.03 jk
GN-22	0.62 r-q	5.00 e-j	9.55 e-n	28.46 c-r	5.22 n-u	1.61 f-y	1.79 n-y	6.49 a-u	5.66 f-h
GN-23	1.27 b-h	0.90 f-k	3.76 h-v	26.11 e-s	4.76 r-v	4.89 a-v	6.25 b-r	10.51 ab	5.46 h-j
GN-24	0.57 t-s	9.74 b-f	10.03 e-m	27.80 c-r	7.31 c-d	5.64 a-s	7.84 b-l	2.36 h-y	2.33 k-p
GN-25	1.22 b-ı	6.69 d-j	10.24 e-m	23.48 g-u	5.70 k-q	5.32 a-v	8.08 b-l	5.15 b-v	0.05 kl
GN-26	0.37 d-v	7.61 b-j	10.85 c-k	25.84 e-s	10.71 a-g	5.98 a-n	7.55 b-l	1.28 n-y	1.64 l-t
GN-27	0.61 r-q	7.56 b-j	11.08 c-j	24.08 f-u	3.27 c-2	3.63 b-y	5.35 c-y	5.22 b-v	1.57 l-t
GN-28	0.54 t-t	6.64 d-j	7.76 e-r	19.21 j-y	0.58 v-2	3.65 b-y	5.36 c-y	7.79 a-m	2.98 kl
GN-29	1.37 b-f	7.62 b-j	11.49 c-h	21.65 ı-v	7.68 a-c	5.56 a-t	5.54 b-y	1.79 k-y	7.88 e-n
GN-30	0.45 a-v	8.16 b-j	8.80 e-n	23.51 f-u	7.33 c-d	4.86 a-v	7.97 b-l	5.19 b-v	1.06 l-z
GN-31	0.59 t-r	2.27 f-k	5.56 f-v	22.36 ı-v	2.14 m-2	2.61 b-y	3.25 j-y	12.58 a	7.28 g-r
GN-32	0.59 t-r	3.97 e-j	5.82 f-u	18.91 j-y	8.99 a-r	6.05 a-l	6.28 b-r	4.72 b-v	7.53 f-p
GN-33	1.15 c-p	4.62 e-j	6.34 f-u	21.75 ı-v	7.80 c-z	5.00 a-v	4.96 d-y	4.95 b-v	1.68 l-t
GN-34	1.18 b-o	0.30 g-k	0.31 r-v	0.75 za	0.33 z12	0.06 uvy	0.17 vy	0.36 t-y	0.04 kl
GN-35	0.74 j-l	7.80 b-j	9.41 e-n	18.77 j-y	6.88 d-1	4.74 a-v	6.06 b-r	3.90 d-v	7.23 g-r
GN-36	0.55 t-t	8.46 b-g	12.25 c-f	20.05 j-y	8.59 a-t	6.16 a-l	6.93 b-o	2.26 h-y	1.25 l-y
GN-37	0.60 r-t	9.44 b-g	11.46 c-h	21.81 ı-v	4.86 p-v	3.82 b-y	7.66 b-l	2.85 e-v	8.38 e-k
GN-38	0.65 r-q	7.22 b-j	6.13 f-u	35.42 a-k	0.34 z12	0.09 uvy	0.20 uvy	0.60 r-y	5.09 ı-k
GN-39	0.42 b-v	0.47 ijk	0.70 s-v	32.50 a-p	2.40 k-2	4.48 a-v	3.68 y	8.52 a-l	4.03 jk
GN-40	0.23 n-v	7.16 b-j	5.58 f-v	33.56 a-n	4.76 r-v	3.82 b-y	10.40 bcd	5.25 b-v	13.08 cde
GN-41	0.84 g-e	3.77 e-j	8.56 e-p	30.53 b-r	4.94 o-u	4.72 a-v	8.84 b-l	8.40 a-j	2.76 klm
GN-42	1.23 b-j	9.09 b-h	10.79 c-k	5.18 v-a	8.72 a-s	6.02 a-n	6.42 b-q	5.43 b-v	1.69 l-t
GN-43	1.29 b-g	4.05 e-j	6.03 f-u	28.22 c-r	5.37 m-s	5.05 a-v	6.89 b-o	8.79 a-f	4.26 ı-k

GN-44	0.95 d-z	3.67 e-k	7.96 e-r	24.38 e-u	6.72 d-j	5.20 a-v	3.58 ı-y	6.10 b-v	2.87 klm
GN-45	0.51 v-t	8.66 b-j	0.92 tuv	21.02 ı-y	11.43 abc	7.61 abc	7.13 b-o	0.18 uvy	0.80 c-l
GN-46	1.10 c-r	0.34 g-k	0.80 p-v	7.97 t-a	0.08 -12	0.28 s-y	0.89 r-y	2.80 e-y	0.01 kl
GN-47	0.06 tuv	3.58 e-k	4.37 g-v	44.33 abc	2.77 g-2	3.68 b-y	10.59 bc	0.72 q-y	6.38 g-y
GN-48	0.62 r-q	9.48 b-g	10.52 d-l	21.69 ı-v	7.87 c-z	5.33 a-v	6.45 b-q	2.65 e-y	2.65 k-n
GN-49	0.20 p-v	5.42 d-j	7.99 e-r	25.58 e-t	5.64 k-q	5.17 a-v	7.69 b-l	6.41 a-v	5.62 ggh
GN-50	0.79 h-l	4.96 e-j	7.87 e-r	30.14 b-r	3.58 z-2	6.34 a-j	7.64 b-l	6.62 a-t	5.58 h-k
GN-51	0.54 t-t	6.40 d-j	7.81 e-r	27.28 c-r	7.66 b-c	6.24 a-k	7.70 b-l	5.29 b-v	5.39 h-j
GN-52	1.02 d-t	5.46 d-j	7.66 e-r	21.92 ı-v	9.14 a-p	6.32 a-j	5.40 c-y	6.00 b-v	0.31 h-l
GN-53	0.69 o-p	0.06 h-k	0.98 tuv	41.93 a-e	2.91 f-2	2.47 b-y	4.63 e-y	0.02 vy	23.61 a
GN-54	0.69 p-r	4.42 e-j	7.84 e-r	15.60 o-a	5.21 n-u	5.10 a-v	5.68 b-u	6.14 b-v	5.96 d-h
GN-55	0.69 p-r	4.87 e-j	8.95 e-n	22.72 h-v	6.74 d-j	5.56 a-t	6.76 b-o	6.73 a-t	0.02 kl
GN-56	0.34 f-v	1.01 f-k	7.06 e-s	24.61 e-u	6.51 e-1	5.76 a-p	6.58 b-p	7.87 a-m	0.70 d-l
GN-57	0.44 b-v	7.09 b-j	10.07 e-m	29.82 c-r	2.29 ı-2	3.29 b-y	8.46 b-j	7.61 a-n	2.80 klm
GN-58	1.21 b-m	6.30 d-j	9.35 e-n	23.08 h-u	7.54 c-e	4.42 a-v	5.13 c-y	4.71 b-v	4.57 ı-k
GN-59	0.30 ı-v	0.90 f-k	4.24 g-v	28.51 c-r	4.16 u-2	5.01 a-v	7.00 b-o	7.44 a-o	4.77 ı-k
GN-60	0.58 t-s	1.21 f-k	7.48 e-r	44.03 a-d	2.47 j-2	4.27 a-v	1.63 o-y	4.61 b-v	15.07 bcd
GN-61	0.78 h-l	7.37 b-j	8.96 e-n	22.75 h-v	6.63 d-k	5.26 a-v	7.52 b-l	6.53 a-u	0.01 kl
GN-62	0.66 r-q	5.33 e-j	9.33 e-n	24.95 e-u	5.51 s-s	5.57 a-s	7.42 b-m	6.07 b-v	7.12 g-r
GN-63	0.74 k-m	5.69 d-j	7.71 e-r	15.08 p-a	2.68 h-2	1.05 j-y	4.81 e-y	5.00 b-v	4.41 ı-k
GN-64	0.42 b-v	8.43 b-j	12.34 c-f	19.68 j-y	8.18 b-v	5.93 a-o	6.60 b-p	1.83 k-y	1.66 l-t
GN-65	0.66 r-q	0.86 f-k	2.14 n-v	15.62 o-a	5.76 k-q	5.86 a-o	6.58 b-p	7.44 a-o	6.57 g-v
GN-66	0.81 g-g	2.99 f-k	4.93 f-v	18.66 j-y	3.34 c-2	1.30 t-y	5.22 c-y	8.03 a-l	9.40 e-l
GN-67	0.32 h-v	8.67 b-j	7.09 e-s	26.62 d-s	10.85 a-d	6.47 a-l	9.25 b-h	2.28 h-y	1.42 l-u
GN-68	0.86 g-c	3.36 e-k	4.67 f-v	28.95 c-r	3.98 v-2	4.23 a-v	6.40 b-q	6.65 a-t	10.47 d-h
GN-69	0.60 r-s	5.49 d-j	7.55 e-r	18.08 k-a	9.17 a-o	6.42 a-j	5.97 b-s	2.87 e-v	6.60 g-v
GN-70	0.78 ı-ı	6.97 b-j	9.22 e-n	21.55 ı-y	8.09 b-y	5.40 a-v	6.54 b-p	3.03 e-v	2.91 kl
GN-71	0.60 r-s	7.65 b-j	8.02 e-r	23.12 h-u	4.68 s-y	4.45 a-v	7.69 b-l	5.21 b-v	7.53 f-p
GN-72	0.32 g-v	12.52 b-e	10.87 c-k	36.07 a-j	0.99 t-2	0.79 l-y	0.96 q-y	0.14 uvy	0.73 d-l
GN-73	0.60 r-t	0.90 f-k	4.87 f-v	47.69 ab	9.64 a-m	6.99 a-f	9.08 b-l	5.82 b-v	0.01 kl
GN-74	1.09 c-s	6.01 d-j	9.05 e-n	23.93 f-u	9.68 a-l	6.80 a-g	6.39 b-r	2.63 e-y	6.69 g-u
GN-75	0.76 ı-k	4.25 e-j	3.91 h-v	18.33 k-z	4.02 u-2	3.49 b-y	9.42 b-g	5.32 b-v	6.66 g-u
GN-76	0.44 b-v	14.76 a-d	18.08 bcd	27.98 c-r	6.25 ı-n	4.95 a-v	7.38 b-m	4.04 c-v	5.73 e-h
GN-77	0.86 g-c	7.35 b-j	4.64 f-v	29.22 c-r	6.17 ı-n	4.65 a-v	8.26 b-l	4.38 b-v	5.29 h-k
GN-78	0.52 v-t	5.01 e-j	7.34 e-r	31.20 b-r	7.02 d-g	5.14 a-v	8.41 b-k	2.98 e-v	5.02 ı-k
GN-79	0.63 r-q	2.98 f-k	4.95 f-v	41.15 a-f	4.17 u-1	4.09 b-y	7.51 b-l	2.45 f-y	12.69 c-f
GN-80	0.85 g-d	2.75 f-k	7.35 e-r	19.67 j-y	6.66 d-k	7.75 ab	2.95 k-y	8.77 a-g	7.00 g-s
GN-81	1.42 b-e	5.50 d-j	8.48 e-p	21.81 ı-v	8.20 b-v	5.57 a-s	7.31 b-m	2.73 e-y	5.53 h
GN-82	0.50 v-t	2.75 f-k	5.78 f-u	29.23 c-r	1.58 p-2	6.26 a-k	6.78 b-o	3.91 d-v	8.16 e-l
GN-83	0.54 t-t	5.72 d-j	10.93 c-k	23.53 f-u	4.84 q-v	3.95 b-y	6.84 b-o	1.76 k-y	7.73 f-o
GN-84	0.43 b-v	3.87 e-j	1.93 n-v	15.77 o-a	6.74 d-j	4.49 a-v	24.90 a	1.96 k-y	2.55 o-k
GN-85	0.72 ı-m	3.82 e-j	7.87 e-r	22.63 ı-v	6.52 e-1	5.08 a-v	6.76 b-o	5.21 b-v	6.03 c-h
GN-86	0.33 g-v	1.59 f-k	5.06 f-v	15.99 n-a	4.22 u-1	5.58 a-s	6.81 b-o	7.06 a-q	4.86 ı-k
GN-87	0.68 o-p	7.46 b-j	11.29 c-l	23.21 h-u	4.75 r-y	0.57 o-y	5.93 b-s	0.52 s-y	2.69 klm
GN-88	1.65 b	7.44 b-j	8.98 e-n	24.00 f-u	10.31 a-l	1.47 g-y	0.49 s-y	3.68 d-v	0.80 c-l
GN-89	1.13 c-q	3.95 e-j	7.02 e-s	21.30 ı-y	7.37 c-d	6.47 a-l	6.74 b-o	3.89 d-v	7.71 f-o
GN-90	0.46 z-v	0.04 h-k	2.41 m-v	30.21 b-r	5.91 j-o	6.88 a-f	8.96 b-l	3.63 d-v	4.13 ı-k
GN-91	0.44 b-v	9.11 b-h	0.96 tuv	22.41 ı-v	10.79 a-e	6.09 a-l	7.46 b-l	2.15 l-y	0.86 b-l

GN-92	1.20 b-m	8.03 b-j	10.88 c-k	24.53 e-u	10.08 a-j	5.34 a-v	6.52 b-p	0.87 p-y	7.07 g-r
GN-93	0.94 e-a	4.36 e-j	3.35 j-v	25.34 e-u	3.18 d-2	7.14 a-e	7.49 b-l	7.91 a-m	8.55 e-k
GN-94	0.38 c-v	6.59 d-j	7.60 e-r	24.34 e-u	7.10 d-f	5.54 a-t	7.76 b-l	2.67 e-y	5.44 h-j
GN-95	0.56 t-s	6.79 c-j	9.06 e-n	16.92 m-a	6.89 d-1	6.62 a-l	5.59 b-v	4.39 b-v	0.22 jkl
GN-96	0.89 f-b	7.27 b-j	8.74 e-o	30.59 b-r	7.32 c-d	4.59 a-v	7.45 b-l	5.15 b-v	5.12 ı-k
GN-97	0.47 z-u	5.86 d-j	8.20 e-q	26.67 d-s	7.18 c-f	6.53 a-l	6.29 b-r	5.10 b-v	6.14 b-h
GN-98	0.99 d-v	6.48 d-j	7.70 e-r	30.85 b-r	6.42 g-m	4.70 a-v	7.81 b-l	7.10 a-q	6.44 g-y
GN-99	0.59 t-s	6.48 d-j	7.70 e-r	30.85 b-r	6.42 g-m	4.70 a-v	7.81 b-l	7.10 a-q	6.44 g-y
GN-100	0.58 t-s	7.33 b-j	9.52 e-n	31.76 a-q	7.14 c-f	4.79 a-v	9.23 b-h	1.27 n-y	1.49 l-u
GN-101	0.23 n-v	6.68 d-j	9.36 e-n	23.53 f-u	8.12 b-y	5.88 a-o	7.75 b-l	3.98 c-v	2.00 l-r
GN-102	0.02 uv	4.92 e-j	6.04 f-u	27.12 c-r	4.84 q-v	4.26 a-v	9.46 b-f	3.88 d-v	5.77 e-h
GN-103	0.14 r-v	0.65 jk	1.11 uv	0.70 za	0.97 u-2	0.39 p-y	0.20 uvy	0.14 uvy	0.72 d-l
GN-104	0.52 v-t	9.57 b-g	14.66 cde	38.11 a-l	3.37 b-2	2.25 c-y	6.81 b-o	4.88 b-v	7.07 g-r
GN-105	1.29 b-g	5.80 d-j	10.23 e-m	27.15 c-r	10.73 a-f	6.73 a-h	7.50 b-l	0.53 s-y	6.43 g-y
GN-106	0.83 g-f	16.14 ab	26.87 a	25.62 e-s	7.98 c-y	6.00 a-n	7.64 b-l	4.02 c-v	4.19 ı-k
GN-107	0.86 g-c	16.12 abc	24.25 ab	26.08 e-s	6.60 d-k	5.13 a-v	7.12 b-o	4.99 b-v	1.76 l-s
GN-108	0.97 d-y	3.36 e-k	8.50 e-p	28.93 c-r	4.94 o-u	5.46 a-u	7.10 b-o	7.37 a-o	2.58 k-o
GN-109	0.38 c-v	2.84 f-k	7.14 e-r	27.76 c-r	4.55 s-z	5.01 a-v	7.61 b-l	7.39 a-o	7.72 f-o
GN-110	0.50 v-t	4.77 e-j	5.81 f-u	20.27 j-y	6.65 d-k	7.23 a-d	5.72 b-t	3.72 d-v	4.95 ı-k
GN-111	0.78 h-l	3.32 e-k	8.37 e-p	34.79 a-l	3.92 v-2	4.74 a-v	6.86 b-o	8.95 a-e	6.80 g-t
GN-112	0.60 r-s	9.43 b-g	9.06 e-n	23.97 f-u	10.85 a-d	6.45 a-j	8.11 b-l	0.71 q-y	2.97 kl
GN-113	0.71 ı-n	4.15 e-j	6.03 f-u	28.01 c-r	0.10 -12	0.29 r-y	3.95 g-y	2.99 e-v	11.54 c-g
GN-114	0.41 b-v	2.18 f-k	2.91 f-k	48.90 a	0.62 v-2	1.91 d-y	6.10 b-r	4.72 b-v	5.89 e-h
GN-115	0.71 ı-n	2.56 f-k	2.94 f-k	13.99 r-a	1.67 o-2	2.40 b-y	9.97 b-e	4.92 b-v	4.34 ı-k
GN-116	0.86 g-c	5.03 e-j	5.92 f-u	27.17 c-r	6.00 j-n	4.66 a-v	8.94 b-l	5.37 b-v	1.43 l-u
GN-117	0.50 v-t	3.22 f-j	4.97 f-v	19.80 j-y	6.97 d-h	6.76 a-g	5.76 b-s	3.55 d-v	5.90 e-h
GN-118	1.55 bc	0.42 f-k	4.30 g-v	31.58 a-r	5.70 k-q	5.25 a-v	7.59 b-l	9.48 a-d	5.35 h-j
GN-119	0.51 v-t	9.08 b-h	11.10 c-j	7.77 u-a	1.47 q-2	5.74 a-p	4.81 e-y	3.43 d-v	2.10 k-r
GN-120	0.44 b-v	6.51 d-j	7.27 e-r	25.34 e-u	12.35 ab	6.60 a-l	7.89 b-l	2.83 e-y	1.64 l-t
GN-121	0.30 ı-v	3.72 e-j	7.35 e-r	19.21 j-y	2.37 k-2	4.01 b-y	5.89 b-s	10.33 abc	0.40 f-l
GN-122	0.10 s-v	5.03 e-j	5.18 f-v	25.68 e-s	6.75 d-j	5.42 a-v	7.21 b-n	2.59 e-y	5.00 ı-k
GN-123	1.52 bc	5.21 e-j	8.39 e-p	28.51 c-r	7.56 c-e	6.03 a-m	5.93 b-s	5.29 b-v	6.18 a-h
GN-124	0.58 t-s	8.73 b-ı	9.23 e-n	21.40 ı-y	9.29 a-n	5.08 a-v	7.22 b-n	2.78 e-y	2.60 n-k
GN-125	1.44 bcd	1.32 f-k	11.91 c-g	27.31 c-r	4.68 s-y	4.53 a-v	6.20 b-r	6.97 a-r	6.32 g-z
GN-126	0.57 t-s	5.77 d-j	9.46 e-n	26.28 e-s	7.78 a-c	7.06 a-e	4.35 f-y	1.59 m-y	7.97 e-m
GN-127	0.33 g-v	3.59 e-k	8.19 e-q	28.47 c-r	3.84 y-2	6.65 a-l	6.68 b-o	4.03 c-v	1.32 l-v
GN-128	0.75 j-k	23.73 a	18.44 bc	26.74 c-s	5.72 k-q	5.45 a-v	8.00 b-l	5.08 b-v	4.24 ı-k
GN-129	0.70 n-o	1.54 f-k	4.87 f-v	32.41 a-p	7.40 c-d	5.69 a-r	8.42 b-k	8.49 a-l	1.25 l-y
GN-130	0.68 o-p	7.20 b-j	9.62 e-n	19.60 j-y	9.85 a-k	5.38 a-v	8.30 b-k	0.72 q-y	0.87 b-l
GN-131	0.27 k-v	3.95 e-j	9.17 e-n	32.51 a-p	5.77 k-p	3.65 b-y	8.99 b-l	4.09 c-v	2.76 klm
GN-132	0.35 e-v	4.78 e-j	4.70 f-v	36.23 a-j	1.03 t-2	0.06 uvy	0.22 t-y	1.12 o-y	4.91 ı-k
GN-133	1.19 b-n	5.04 e-j	8.81 e-n	23.03 h-u	7.68 a-c	8.81 a-n	0.21 uvy	0.89 p-y	3.51 jk
GN-134	0.81 g-h	2.06 f-k	4.02 h-v	35.42 a-k	0.03	1.94 d-y	4.31 f-y	5.91 b-v	16.09 bc
GN-135	0.62 r-q	4.60 e-j	6.65 f-u	18.08 k-a	7.89 c-y	7.30 a-d	7.84 b-l	6.80 a-s	0.04 kl
GN-136	0.32 g-v	8.98 b-h	12.30 c-f	19.84 j-y	8.05 c-y	6.33 a-j	5.29 c-y	1.99 k-y	6.39 g-y
GN-137	0.52 u-t	1.42 f-k	4.52 f-v	24.67 e-u	0.36 z12	2.60 b-y	3.76 h-y	7.23 a-p	5.93 d-h
GN-138	0.41 b-v	6.71 d-j	8.56 e-p	16.27 m-a	5.51 ı-r	5.17 a-v	7.03 b-o	7.14 a-p	5.00 ı-k
GN-139	0.67 o-p	5.11 e-j	8.89 e-n	26.93 c-s	9.14 a-p	7.41 abc	5.56 b-y	3.92 d-v	3.27 kl

GN-140	0.28 j-v	1.43 f-k	2.79 ı-v	40.29 a-h	5.11 n-u	3.10 b-y	4.29 f-y	2.38 g-y	19.10 ab
GN-141	0.33 g-v	4.84 e-j	9.09 e-n	29.15 c-r	4.94 o-u	4.33 a-v	8.81 b-l	6.89 a-s	1.37 l-v
GN-142	1.01 d-u	7.17 b-j	8.80 e-n	34.95 a-l	0.29 z12	0.32 q-y	1.94 m-y	7.36 a-o	0.03 kl
GN-143	0.21 o-v	3.28 e-k	8.13 e-r	23.28 h-u	6.17 ı-n	5.95 a-o	5.48 b-y	4.87 b-v	5.20 h-k
GN-144	0.73 l-m	7.08 b-j	10.28 d-l	20.24 j-y	5.97 j-n	4.39 a-v	4.09 f-y	4.62 b-v	0.63 e-l
GN-145	0.49 y-t	6.42 d-j	8.61 e-p	24.80 e-u	6.30 h-m	5.78 a-p	7.33 b-m	5.27 b-v	3.98 jk
GN-146	0.88 f-b	9.03 b-h	10.82 c-k	22.67 h-v	6.51 e-1	4.34 a-v	6.74 b-o	3.75 d-v	5.04 ı-k
GN-147	0.66 r-q	5.33 e-j	7.84 e-r	0.80 za	5.13 n-u	4.83 a-v	3.59 ı-y	5.81 b-v	4.05 jk
GN-148	0.61 r-q	5.29 e-j	7.35 e-r	14.59 q-a	6.27 h-n	5.28 a-v	5.45 b-y	5.86 b-v	4.85 ı-k
GN-149	0.38 c-v	5.32 e-j	9.23 e-n	23.65 f-u	5.00 n-u	5.97 a-o	7.98 b-l	6.16 b-v	2.00 l-r
GN-150	0.43 b-v	0.34 g-k	0.80 p-v	9.47 s-a	1.21 s-2	0.05 vy	5.46 b-y	1.26 n-y	0.03 kl
GN-151	0.35 f-v	0.34 g-k	0.80 p-v	9.47 s-a	1.21 s-2	0.05 vy	5.46 b-y	1.26 n-y	0.03 kl
GN-152	0.48 y-t	0.82 f-k	0.92 o-v	29.80 c-r	3.49 a-2	4.94 a-v	10.95 b	8.13 a-k	7.45 f-q
GN-153	0.95 d-z	6.06 d-j	9.67 e-n	31.14 b-r	7.23 c-e	5.02 a-v	9.04 b-l	2.36 h-y	1.16 l-y
GN-154	2.25 a	5.35 e-j	4.27 g-v	19.85 j-y	4.25 u-1	4.27 g-v	7.60 b-l	4.94 b-v	6.85 g-t
GN-155	0.57 t-s	8.69 b-j	5.79 f-u	23.86 f-u	9.06 a-q	6.57 a-l	8.41 b-k	4.20 b-v	4.18 ı-k
GN-156	0.26 l-v	5.51 d-j	7.08 e-s	24.48 e-u	10.56 a-h	7.08 e-s	6.69 b-o	3.77 d-v	0.87 b-l
GN-157	0.55 t-t	3.83 e-j	0.90 o-v	0.75 za	0.35 z12	0.88 k-y	5.17 c-y	6.63 a-t	8.56 e-k
GN-158	0.41 b-v	3.79 e-j	0.41 q-v	7.92 u-a	1.06 t-2	1.33 h-y	7.03 b-o	6.68 a-t	9.36 e-l
GN-159	0.70 n-o	4.62 e-j	4.18 g-v	23.84 f-u	2.61 ı-2	0.63 m-y	7.58 b-l	3.28 d-v	4.64 ı-k
GN-160	0.36 d-v	4.92 e-j	6.73 f-t	17.00 m-a	2.71 h-2	3.17 b-y	3.75 h-y	6.82 a-s	5.35 h-j

Differences between means indicated with the same letter are not significant. EOC: Essential oil content.

Table S10. Minor essential oil compositions of coriander genotypes.

G/C Code	Monoterpene											Aldehydes		Alcohols
	Camphene	Sabiene	2-β-pinene	a-Mycrene	a-Terpinen	d-Limonen	Cymol	a-Ocimene	ç-terpinene	a-Terpinolene	Epoxi Linalool	n-Octanal	2,2-Decadienal	1-Octanol
**RT**	14.99	15.89	16.01	16.27	17.36	17.77	17.91	18.29	18.72	19.85	20.5	12.6	20.11	20.78
GN-1	0.74	0.53	0.59	1.08	0.08	2.32	4.14	0.08	8.94	0.63	0.14	0.03	0.16	0.17
GN-2	0.03	0.04	0.14	0.02	0.01	0.02	0.01	0.01	0.04	0.02	0.02	0.06	0.02	0.03
GN-3	0.67	0.47	0.61	1.28	0.07	2.87	3.59	0.08	9.20	0.83	0.17	0.02	0.03	0.24
GN-4	1.19	0.60	0.68	1.43	0.15	3.33	2.37	0.11	10.28	1.01	0.11	0.02	0.17	0.16
GN-5	1.04	0.53	0.87	1.25	0.07	2.87	3.71	0.10	8.22	0.75	0.22	0.03	0.17	0.22
GN-6	0.65	0.48	0.90	0.98	0.02	0.07	1.98	3.45	0.18	9.76	0.04	0.02	0.54	0.15
GN-7	0.89	0.53	0.56	1.10	0.05	2.50	4.13	0.09	6.39	0.65	0.19	0.04	0.14	0.17
GN-8	0.18	0.55	0.88	0.58	0.01	0.08	4.67	0.13	9.85	0.12	0.17	0.03	0.16	0.06
GN-9	0.09	0.12	0.16	0.18	0.01	0.39	2.15	0.04	2.91	0.09	0.13	0.02	0.18	0.05
GN-10	1.01	0.73	0.85	1.53	0.11	3.38	5.61	0.11	10.92	0.94	0.22	0.02	0.08	0.31
GN-11	0.04	0.07	0.08	0.03	0.21	0.56	1.07	0.99	0.06	0.01	0.12	0.01	0.20	0.01
GN-12	0.35	0.44	0.51	0.90	0.10	1.65	4.38	0.11	8.66	0.48	0.33	0.05	0.24	0.17
GN-13	0.46	0.28	0.43	0.70	0.02	1.69	4.24	0.06	4.66	0.35	0.26	0.03	0.36	0.04
GN-14	1.00	0.57	0.70	1.24	0.11	2.82	2.52	0.11	9.93	0.84	0.11	0.02	0.00	0.22
GN-15	0.94	0.56	0.90	1.18	0.10	2.53	3.43	0.10	9.20	0.65	0.14	0.03	0.07	0.23
GN-16	0.60	0.39	0.60	0.84	0.05	1.80	3.53	0.07	6.92	0.45	0.20	0.06	0.17	0.08
GN-17	0.01	0.65	0.58	0.90	0.01	1.97	2.02	0.01	4.58	0.45	0.01	0.02	0.01	0.04
GN-18	0.06	0.05	0.08	0.11	0.02	0.31	0.80	1.20	1.80	0.10	0.22	0.01	0.02	0.20
GN-19	0.78	0.49	0.40	1.20	0.10	3.02	3.12	0.13	8.97	0.92	0.24	0.03	0.03	0.31
GN-20	0.32	0.00	0.00	0.32	0.35	0.67	0.09	1.62	3.29	0.71	0.61	0.01	9.34	0.09
GN-21	0.50	0.07	0.38	0.15	0.03	1.23	1.94	0.02	5.43	0.30	0.02	0.11	0.03	0.02
GN-22	0.58	0.46	0.61	1.04	0.08	2.08	4.00	0.07	8.75	0.64	0.14	0.09	0.08	0.24
GN-23	0.08	0.07	0.10	0.19	0.02	0.54	1.67	0.68	3.45	0.20	0.22	0.01	0.03	0.20
GN-24	0.85	0.71	1.29	1.27	0.09	2.13	5.28	0.05	11.14	0.49	0.25	0.04	0.09	0.22
GN-25	1.15	0.77	0.89	1.49	0.19	2.86	2.91	0.09	9.44	0.91	0.05	0.03	0.23	0.11
GN-26	1.30	0.82	0.95	1.57	0.11	3.49	4.95	0.13	11.96	0.98	0.15	0.03	0.07	0.27

GN-27	0.54	0.66	0.89	0.99	0.07	1.72	5.89	0.06	10.77	0.40	0.25	0.04	0.08	0.24
GN-28	0.19	0.37	0.80	0.59	0.04	0.65	4.46	0.09	7.45	0.14	0.24	0.05	0.17	0.10
GN-29	1.58	0.78	0.77	1.67	0.13	3.65	2.60	0.12	11.18	1.09	0.08	0.02	0.03	0.21
GN-30	0.85	0.60	1.07	1.23	0.09	2.38	3.98	0.08	9.91	0.63	0.18	0.04	0.15	0.20
GN-31	0.17	0.24	0.24	0.38	0.03	0.84	4.48	0.03	5.25	0.18	0.20	0.04	0.07	0.09
GN-32	0.88	0.44	0.54	1.03	0.06	2.43	4.02	0.15	6.93	0.63	0.22	0.04	0.15	0.14
GN-33	0.89	0.43	0.48	0.93	0.04	2.35	4.76	0.09	6.03	0.51	0.21	0.04	0.17	0.07
GN-34	*nd*	*nd*	*nd*	*nd*	*nd*	*nd*	*nd*	*nd*	*nd*	*nd*	*nd*	*nd*	*nd*	*nd*
GN-35	1.23	0.66	0.89	1.46	0.09	3.19	3.35	0.09	9.10	0.89	0.14	0.04	0.10	0.21
GN-36	1.71	0.95	0.94	1.97	0.13	4.30	3.97	0.14	11.94	1.25	0.11	0.01	0.02	0.30
GN-37	0.90	0.73	1.05	1.29	0.12	2.31	3.62	0.05	11.15	0.62	0.12	0.02	0.01	0.23
GN-38	0.52	0.34	0.71	0.72	0.03	1.45	3.72	0.04	5.82	0.31	0.15	0.03	0.10	0.07
GN-39	0.03	0.03	0.06	0.04	0.02	0.11	0.42	0.47	0.41	0.02	0.36	0.01	0.01	0.01
GN-40	0.50	0.29	0.83	0.93	0.06	1.87	1.20	0.17	4.78	0.57	0.09	0.02	0.08	0.12
GN-41	0.25	0.31	0.50	0.66	0.05	1.41	3.64	0.22	7.76	0.44	0.14	0.02	0.07	0.08
GN-42	1.90	0.96	1.39	2.16	0.17	4.31	3.78	0.16	10.48	1.32	0.20	0.03	0.21	0.23
GN-43	0.55	0.33	0.45	0.87	0.02	2.04	4.17	0.13	5.23	0.49	0.30	0.05	0.12	0.12
GN-44	0.61	0.42	0.41	0.88	0.07	2.08	3.58	0.12	7.65	0.52	0.15	0.06	0.11	0.15
GN-45	2.48	1.29	1.22	2.68	0.06	0.21	5.42	3.97	0.19	12.84	0.04	0.02	1.71	0.12
GN-46	*nd*	*nd*	0.03	0.01	*nd*	*nd*	*nd*	0.01	*nd*	*nd*	0.09	0.02	*nd*	0.01
GN-47	0.22	0.16	0.36	0.35	0.02	0.68	1.28	0.03	3.57	0.12	0.02	0.02	0.05	0.02
GN-48	1.53	0.73	1.06	1.71	0.09	3.75	3.98	0.10	10.21	1.03	0.13	0.02	0.04	0.20
GN-49	0.58	0.41	0.64	0.91	0.06	2.05	3.75	0.08	7.19	0.62	0.10	0.10	0.09	0.08
GN-50	0.29	0.34	0.54	0.64	0.06	0.90	2.72	0.08	7.07	0.34	0.12	0.02	0.11	0.14
GN-51	1.12	0.49	0.78	1.36	0.08	3.14	2.76	0.10	7.01	0.93	0.14	0.05	0.12	0.15
GN-52	0.97	0.48	0.60	1.19	0.07	2.84	3.29	0.11	7.35	0.75	0.19	0.17	0.03	0.18
GN-53	0.09	0.08	0.11	0.15	0.01	0.47	1.44	2.07	0.13	0.01	0.04	0.02	0.04	0.07
GN-54	0.30	0.45	0.64	0.99	0.08	1.91	5.31	0.19	7.04	0.63	0.40	0.05	0.27	0.37
GN-55	0.71	0.56	0.60	1.21	0.08	2.56	5.64	0.14	8.15	0.78	0.25	0.03	0.21	0.31
GN-56	0.10	0.12	0.15	0.27	0.04	0.73	2.24	1.02	6.75	0.32	0.27	0.01	0.18	*nd*
GN-57	0.29	0.55	0.85	0.69	0.07	0.87	4.66	0.07	9.27	0.19	0.14	0.04	0.16	0.15

GN-58	1.00	0.52	0.68	1.16	0.10	2.66	2.65	0.14	9.04	0.76	0.14	0.04	0.14	0.13
GN-59	0.07	0.07	0.10	0.16	0.02	0.45	1.46	1.00	3.93	0.19	0.21	0.01	0.07	0.22
GN-60	0.08	0.12	0.16	0.22	0.04	0.44	1.97	0.60	6.68	0.15	0.11	0.01	0.03	0.25
GN-61	1.19	0.65	1.03	1.60	0.12	3.07	3.18	0.10	8.16	1.05	0.13	0.05	0.13	0.26
GN-62	0.54	0.54	0.69	1.04	0.11	2.05	3.36	0.13	8.53	0.65	0.14	0.02	0.14	0.24
GN-63	0.51	0.45	0.62	0.78	0.06	1.52	3.67	0.05	7.40	0.36	0.12	0.04	0.10	0.12
GN-64	1.54	0.95	1.06	1.88	0.15	3.95	5.36	0.07	12.03	1.12	0.18	0.02	0.08	0.22
GN-65	0.07	0.06	0.10	0.13	0.02	0.30	0.81	1.33	1.34	0.10	0.23	0.02	0.56	0.11
GN-66	0.34	0.29	0.36	0.62	0.04	1.32	3.21	0.05	4.13	0.31	0.14	0.03	0.05	0.13
GN-67	1.46	0.66	1.11	1.69	0.07	3.72	3.43	0.06	8.20	1.02	0.17	0.02	0.06	0.26
GN-68	0.30	0.50	0.08	0.80	0.01	2.03	2.98	0.01	3.87	0.59	0.01	0.05	0.01	0.02
GN-69	1.25	0.61	0.71	1.37	0.11	3.15	3.22	0.10	8.66	0.90	0.13	0.05	0.19	0.17
GN-70	1.33	0.65	0.75	1.39	0.08	3.28	4.56	0.10	8.91	0.84	0.17	0.05	0.16	0.17
GN-71	0.70	0.52	0.90	1.07	0.05	2.10	4.25	0.07	7.71	0.48	0.23	0.02	0.06	0.25
GN-72	1.66	0.90	1.74	2.44	0.08	4.73	6.48	0.13	11.98	1.23	0.28	0.03	0.13	0.35
GN-73	0.08	0.08	0.12	0.27	0.02	0.89	1.26	0.70	4.07	0.41	0.17	0.01	0.07	0.17
GN-74	1.07	0.66	0.73	1.26	0.12	2.76	3.54	0.11	10.16	0.77	0.16	0.05	0.09	0.21
GN-75	0.45	0.26	0.54	0.70	0.27	1.18	1.14	0.06	5.02	0.41	0.02	0.03	0.09	0.25
GN-76	0.72	0.47	0.67	0.83	0.07	1.74	3.72	0.07	8.05	0.42	0.19	0.03	0.13	0.16
GN-77	0.67	0.35	0.91	0.89	0.05	1.79	1.96	0.07	5.75	0.44	0.11	0.04	0.16	0.09
GN-78	0.61	0.48	0.62	1.02	0.04	2.18	6.28	0.04	8.45	0.47	0.21	0.02	0.06	0.18
GN-79	0.13	0.25	0.40	0.40	0.03	0.54	3.15	0.05	6.06	0.12	0.15	0.02	0.02	0.15
GN-80	0.54	0.33	0.31	0.75	0.07	1.92	2.48	0.19	7.04	0.57	0.13	0.02	0.04	0.15
GN-81	0.77	0.56	0.72	1.13	0.09	2.42	3.99	0.09	9.59	0.67	0.19	0.02	0.19	0.23
GN-82	0.09	0.18	0.32	0.41	0.06	0.62	2.75	0.15	4.98	0.19	0.16	0.03	0.04	0.16
GN-83	0.48	0.67	0.72	0.97	0.11	1.70	5.67	0.06	12.04	0.47	0.13	0.02	0.09	0.10
GN-84	0.74	0.24	0.55	0.84	0.13	2.02	1.00	0.87	3.04	0.67	0.05	0.02	0.11	0.18
GN-85	0.51	0.45	0.53	0.90	0.06	1.94	4.85	0.06	8.98	0.50	0.17	0.03	0.10	0.22
GN-86	0.28	0.31	0.25	0.55	0.03	1.18	4.70	0.07	6.17	0.27	0.25	0.04	0.12	0.13
GN-87	0.53	0.75	0.91	0.99	0.20	1.38	1.91	0.04	12.40	0.37	0.06	0.01	0.02	0.30
GN-88	1.37	0.71	0.92	1.51	0.11	3.34	3.86	0.12	10.09	0.90	0.22	0.02	0.09	0.21

GN-89	0.71	0.44	0.52	0.96	0.09	2.12	2.63	0.18	8.13	0.60	0.10	0.05	0.18	0.15
GN-90	0.09	0.07	0.12	0.17	0.03	0.46	1.13	0.96	3.52	0.20	0.09	0.01	0.09	0.15
GN-91	1.83	1.01	1.25	2.16	0.04	0.17	4.50	3.87	0.15	12.62	0.06	0.01	1.34	0.13
GN-92	0.27	0.19	0.49	0.53	0.01	0.94	2.30	0.07	2.55	0.20	0.29	0.02	0.23	0.16
GN-93	1.26	0.74	0.92	1.64	0.11	3.57	4.34	0.14	11.52	1.01	0.17	0.01	0.07	0.24
GN-94	0.75	0.49	0.85	1.10	0.07	2.24	3.82	0.07	8.71	0.63	0.18	0.02	0.07	0.16
GN-95	1.51	0.68	0.72	1.47	0.07	3.37	5.30	0.10	8.75	0.89	0.17	0.07	0.12	0.17
GN-96	1.14	0.63	0.78	1.28	0.07	2.71	3.39	0.04	7.94	0.69	0.14	0.12	0.12	0.17
GN-97	0.87	0.48	0.63	1.14	0.10	2.53	2.77	0.06	7.40	0.70	0.13	0.04	0.08	0.13
GN-98	0.67	0.39	0.73	1.09	0.07	2.20	2.60	0.07	6.90	0.60	0.13	0.04	0.08	0.15
GN-99	0.50	0.30	0.16	1.00	0.03	1.25	2.36	0.08	4.12	0.45	0.50	0.08	0.17	0.05
GN-100	0.72	0.55	0.86	1.12	0.06	2.16	5.60	0.06	10.63	0.52	0.19	0.03	0.07	0.19
GN-101	1.07	0.61	0.86	1.19	0.11	2.51	3.36	0.10	10.47	0.67	0.09	0.03	0.19	0.14
GN-102	0.52	0.35	0.62	0.77	0.04	1.58	3.33	0.09	7.15	0.38	0.16	0.04	0.26	0.07
GN-103	*nd*	*nd*	*nd*	*nd*	*nd*	*nd*	*nd*	*nd*	*nd*	*nd*	*nd*	*nd*	*nd*	*nd*
GN-104	0.04	0.03	0.06	0.07	0.01	0.22	0.67	0.74	1.56	0.09	0.14	0.01	0.02	0.14
GN-105	1.23	0.68	0.71	1.49	0.12	3.42	3.20	0.15	11.34	1.04	0.13	0.02	0.03	0.24
GN-106	0.89	0.60	0.56	1.28	0.10	3.00	3.97	0.10	9.83	0.83	0.14	0.07	0.03	0.19
GN-107	0.83	0.62	0.79	1.24	0.11	2.48	4.15	0.09	9.55	0.70	0.21	0.05	0.19	0.18
GN-108	0.38	0.40	0.34	0.65	0.05	1.50	4.51	0.07	7.70	0.38	0.17	0.05	0.24	0.15
GN-109	0.32	0.34	0.34	0.65	0.02	1.55	5.91	0.05	6.34	0.34	0.22	0.04	0.13	0.17
GN-110	0.68	0.44	0.69	0.99	0.04	2.17	5.13	0.09	6.92	0.52	0.22	0.05	0.24	0.04
GN-111	0.27	0.31	0.36	0.57	0.05	1.21	3.67	0.05	7.57	0.31	0.16	0.05	0.09	0.13
GN-112	2.13	0.84	1.25	2.20	0.13	4.78	2.97	0.11	10.17	1.45	0.11	0.02	0.04	0.16
GN-113	0.01	0.60	0.10	1.00	0.00	2.60	3.00	0.03	5.23	0.80	0.02	0.05	0.01	0.05
GN-114	0.14	0.10	0.89	0.24	0.02	0.54	0.85	0.01	2.59	0.14	0.02	0.03	0.04	0.02
GN-115	0.23	0.23	0.29	0.99	0.08	0.67	2.96	2.35	2.63	0.16	0.04	0.03	0.21	0.14
GN-116	0.48	0.29	0.62	0.86	0.04	1.99	3.32	0.06	7.03	0.57	0.14	0.03	0.14	0.10
GN-117	0.64	0.37	0.46	0.82	0.05	1.97	3.30	0.07	6.08	0.50	0.13	0.13	0.15	0.05
GN-118	*nd*	0.07	0.10	0.19	0.02	0.61	1.89	0.92	3.50	0.25	0.30	0.51	0.12	0.21
GN-119	0.47	0.57	1.03	1.06	0.14	1.83	4.58	0.18	10.30	0.46	0.25	0.14	0.17	0.20

GN-120	1.63	0.63	0.78	1.65	0.09	3.96	3.10	0.13	8.38	1.11	0.17	0.02	0.11	0.17
GN-121	0.26	0.28	0.39	0.54	0.03	1.04	4.14	0.07	7.04	0.25	0.23	0.03	0.09	0.11
GN-122	0.64	0.32	0.64	0.87	0.05	1.94	2.28	0.06	6.29	0.52	0.13	0.03	0.20	0.06
GN-123	0.92	0.47	0.55	1.01	0.08	2.36	3.14	0.14	7.59	0.64	0.16	0.07	0.17	0.09
GN-124	1.83	0.76	1.00	1.91	0.09	4.27	2.17	0.15	8.92	1.28	0.14	0.02	0.04	0.22
GN-125	0.06	0.05	0.07	0.10	0.02	0.30	1.05	0.91	2.50	0.13	0.24	0.01	0.04	0.21
GN-126	0.90	0.54	0.64	1.33	0.09	3.17	3.56	0.05	9.15	0.87	0.09	0.03	0.10	0.21
GN-127	0.46	0.42	0.41	0.81	0.04	1.86	5.18	0.08	7.39	0.45	0.26	0.03	0.27	0.14
GN-128	0.96	0.46	0.73	1.20	0.06	2.84	3.11	0.07	6.83	0.76	0.17	0.04	0.13	0.15
GN-129	0.18	0.13	0.22	0.45	0.02	1.31	2.46	0.37	4.07	0.39	0.18	0.01	0.19	0.06
GN-130	1.53	0.92	0.97	1.86	0.13	3.92	3.75	0.13	10.73	1.14	0.12	0.01	0.02	0.31
GN-131	0.44	0.33	0.43	0.87	0.06	2.04	3.28	0.07	8.86	0.57	0.15	0.02	0.02	0.20
GN-132	0.15	0.21	0.53	0.49	0.01	0.66	3.73	0.10	4.39	0.13	0.32	0.04	0.07	0.07
GN-133	0.76	0.46	0.63	1.09	0.06	2.68	4.06	0.48	8.50	0.79	0.22	0.04	0.09	0.26
GN-134	0.23	0.13	0.19	0.36	0.02	0.96	1.20	0.02	3.22	0.24	0.01	0.01	0.05	0.01
GN-135	0.71	0.30	0.20	1.05	0.06	2.43	2.83	0.38	5.85	0.85	0.14	0.03	0.20	0.02
GN-136	1.47	0.79	1.02	1.64	0.17	3.44	3.46	0.12	11.99	0.98	0.12	0.02	0.07	0.14
GN-137	0.90	0.43	0.10	0.56	0.08	1.85	2.01	0.06	4.21	0.90	0.30	0.32	0.10	0.04
GN-138	0.54	0.54	1.07	1.24	0.10	2.03	4.49	0.18	7.76	0.77	0.32	0.04	0.47	0.22
GN-139	0.96	0.56	0.62	1.06	0.12	2.44	2.63	0.06	10.00	0.67	0.10	0.02	0.12	0.15
GN-140	0.21	0.18	0.23	0.32	0.02	0.73	1.84	0.02	3.90	0.18	0.11	0.02	0.03	0.10
GN-141	0.47	0.50	0.54	0.90	0.05	1.79	5.58	0.05	8.29	0.42	0.26	0.03	0.10	0.22
GN-142	0.51	0.42	0.81	0.86	0.06	1.52	3.33	0.09	8.00	0.38	0.15	0.03	0.12	0.09
GN-143	0.41	0.43	0.46	0.81	0.09	1.78	4.13	0.04	9.24	0.50	0.14	0.05	0.07	0.16
GN-144	1.10	0.61	0.88	1.39	0.11	2.92	3.15	0.10	7.81	0.91	0.11	0.06	0.12	0.19
GN-145	1.19	0.68	1.02	1.32	0.12	2.74	3.38	0.09	10.51	0.74	0.12	0.02	0.09	0.21
GN-146	0.71	0.67	0.84	1.49	0.15	2.36	5.05	0.34	7.04	0.94	0.49	0.11	0.58	0.36
GN-147	0.36	0.44	0.59	0.85	0.06	1.74	5.13	0.09	9.55	0.45	0.16	0.03	0.07	0.17
GN-148	0.64	0.39	0.68	1.19	0.06	2.67	3.45	0.07	8.43	0.86	0.08	0.21	0.17	0.10
GN-149	*nd*	0.01	*nd*	*nd*	*nd*	*nd*	*nd*	*nd*	*nd*	*nd*	0.85	0.01	*nd*	0.01
GN-150	*nd*	1.24	0.64	0.72	1.46	0.12	3.48	2.95	0.11	9.74	0.06	0.03	0.96	0.10

GN-151	0.06	0.05	0.08	0.11	0.01	1.05	1.10	1.88	0.12	0.03	0.11	0.01	0.18	0.08
GN-152	0.56	0.54	0.70	0.97	0.07	1.87	4.92	0.05	10.78	0.46	0.20	0.02	0.06	0.27
GN-153	0.32	0.31	0.65	0.54	0.02	0.99	3.85	0.05	5.38	0.23	0.14	0.04	0.25	0.12
GN-154	1.33	0.51	1.11	1.44	0.06	3.21	2.49	0.06	6.90	0.87	0.15	0.03	0.08	0.13
GN-155	1.22	0.57	0.66	1.27	0.06	3.07	4.22	0.11	8.19	0.76	0.22	0.09	0.15	0.18
GN-156	0.38	0.26	0.45	0.69	0.04	0.01	1.53	2.34	0.10	4.62	0.13	0.02	0.47	0.34
GN-157	1.39	0.74	1.01	1.51	0.12	3.29	3.81	0.09	12.17	0.88	0.11	0.02	0.05	0.19
GN-158	0.30	0.45	0.65	0.07	0.03	2.15	2.27	0.04	3.87	0.01	0.05	0.10	0.09	0.06
GN-159	1.15	0.65	0.79	1.36	0.13	3.03	2.44	0.09	9.97	0.89	0.08	0.06	0.11	0.18
GN-160	0.10	0.08	0.11	0.17	0.01	0.03	0.50	0.97	1.48	2.76	0.04	0.01	0.24	0.19
Mean	0.70	0.45	0.61	0.96	0.08	1.97	3.30	0.32	7.02	0.88	0.17	0.04	0.21	0.16
SD	0.50	0.25	0.33	0.53	0.12	1.12	1.37	0.67	3.16	1.77	0.11	0.05	0.76	0.08

Table S11. UPOV criteria results of coriander genotypes.

G/C	Vegetative Traits				Flower and Umbel Traits								Fruit and Seed Traits			
	ACH	CS	PH	NBL	LD	GCP	LP	TLS	DLD	LMS	PL	ACF	FS	FBCI	FSH	TBF
GN-1	1	1	3	7	7	7	1	7	7	5	5	1	5	5	1	3
GN-2	1	1	3	7	7	7	1	7	7	5	7	1	7	5	1	3
GN-3	1	1	3	7	7	7	1	7	7	5	7	1	5	7	2	3
GN-4	1	2	5	7	7	7	1	7	7	5	5	1	3	3	1	3
GN-5	1	1	7	5	5	5	1	5	5	3	7	1	7	5	1	7
GN-6	1	2	5	7	7	7	1	7	7	5	7	1	7	5	1	5
GN-7	1	1	3	7	7	7	1	7	7	5	5	1	3	5	1	3
GN-8	1	1	7	7	7	5	1	7	7	5	7	1	7	5	1	5
GN-9	1	2	7	7	5	5	1	5	5	3	5	1	3	5	1	3
GN-10	1	1	7	7	7	7	1	7	7	5	5	1	7	7	1	5
GN-11	1	1	3	7	7	7	1	7	7	5	7	1	7	5	1	3
GN-12	1	2	3	5	5	7	1	5	7	5	5	1	7	5	1	3
GN-13	1	1	3	7	7	7	1	7	7	5	7	1	5	3	1	3
GN-14	1	1	5	7	7	7	1	7	7	3	7	1	7	5	3	7
GN-15	1	1	5	7	7	7	1	7	7	3	7	1	7	7	1	7
GN-16	1	2	7	5	5	5	1	5	7	5	5	1	3	5	1	7
GN-17	1	1	3	7	5	5	1	7	7	5	5	1	7	7	1	3
GN-18	1	2	3	7	7	7	1	7	7	5	7	1	7	5	2	3
GN-19	1	2	7	7	7	7	1	7	7	5	7	1	3	3	3	7
GN-20	1	1	3	7	7	5	1	5	5	5	5	1	3	5	3	5
GN-21	1	2	3	5	5	5	1	5	7	5	5	1	5	3	2	3
GN-22	1	1	7	7	7	7	1	7	7	5	5	1	3	5	1	7
GN-23	1	1	3	5	5	5	1	5	3	5	3	1	3	3	3	3
GN-24	1	2	7	7	7	7	1	7	7	3	7	1	3	7	1	5
GN-25	1	2	5	7	7	7	1	7	7	5	5	1	5	5	2	5
GN-26	1	2	5	7	7	7	1	7	3	3	7	1	5	5	1	5
GN-27	1	2	3	7	7	7	1	7	5	5	7	1	7	5	1	5
GN-28	1	2	5	5	5	5	1	5	5	5	5	1	7	7	2	7
GN-29	1	2	7	5	5	5	1	5	5	5	5	1	3	5	3	7
GN-30	1	2	7	7	7	7	1	7	7	5	7	1	7	5	1	5
GN-31	1	1	3	7	7	7	1	7	7	5	7	1	7	5	2	3
GN-32	1	2	7	7	7	7	1	7	7	5	7	1	5	5	3	7
GN-33	1	2	7	7	7	7	1	5	7	3	7	1	5	5	1	7
GN-34	1	1	5	7	7	7	1	7	7	5	7	1	5	5	2	5
GN-35	1	2	5	7	7	7	1	7	7	3	5	1	5	5	2	5
GN-36	1	2	7	7	7	7	1	7	7	3	7	1	7	5	1	7
GN-37	1	2	7	7	7	7	1	7	5	5	5	1	5	5	2	7
GN-38	1	2	7	5	5	5	1	5	5	5	3	1	7	5	3	7
GN-39	1	2	3	7	7	7	1	7	7	5	7	1	7	3	1	3
GN-40	1	2	3	7	7	5	1	5	5	5	5	1	7	5	3	5
GN-41	1	1	7	7	7	7	1	7	7	5	5	1	5	5	1	7
GN-42	1	2	7	5	5	5	1	5	7	5	5	1	5	3	3	3
GN-43	1	1	7	7	7	7	1	7	7	5	5	1	7	3	2	7
GN-44	1	1	7	5	5	5	1	7	7	5	5	1	7	7	1	3

GN-45	1	2	5	7	7	5	1	7	7	5	7	1	3	7	1	7
GN-46	1	1	3	7	7	7	1	7	7	5	5	1	5	3	1	3
GN-47	1	1	3	7	7	7	1	7	7	5	5	1	5	5	1	3
GN-48	1	2	5	7	7	7	1	7	5	5	5	1	7	5	2	5
GN-49	1	2	7	7	7	7	1	7	7	3	5	1	5	5	1	7
GN-50	1	2	3	5	5	7	1	5	7	5	5	1	3	5	2	3
GN-51	1	1	3	7	7	7	1	5	7	5	5	1	5	3	2	5
GN-52	1	1	3	7	7	7	1	7	7	5	7	1	5	5	1	3
GN-53	1	1	3	7	7	7	1	7	7	5	7	1	7	7	1	3
GN-54	1	2	3	5	5	1	1	5	7	5	5	1	7	5	2	3
GN-55	1	1	3	7	7	7	1	7	7	5	5	1	5	5	2	3
GN-56	1	2	3	7	7	7	1	7	5	3	7	1	3	7	1	3
GN-57	1	1	3	7	7	7	1	7	7	5	5	1	5	5	1	3
GN-58	1	2	7	5	5	5	1	5	7	3	5	1	5	7	1	3
GN-59	1	1	3	7	7	7	1	7	7	3	7	1	3	7	3	5
GN-60	1	2	5	7	7	7	1	7	7	5	7	1	7	5	1	3
GN-61	1	2	5	7	7	7	1	7	7	5	5	1	3	5	3	5
GN-62	1	1	5	7	7	7	1	7	7	5	5	1	7	5	1	5
GN-63	1	1	5	5	5	5	1	5	7	3	7	1	7	5	1	7
GN-64	1	2	7	7	7	5	1	5	7	5	5	1	7	7	1	7
GN-65	1	2	3	7	7	7	1	7	7	5	5	1	5	5	2	3
GN-66	1	2	3	7	7	7	1	7	7	5	5	1	5	5	1	3
GN-67	1	2	7	7	7	7	1	7	7	5	7	1	5	5	1	5
GN-68	1	1	3	7	7	7	1	7	7	5	5	1	5	5	1	3
GN-69	1	2	3	7	7	7	1	7	7	5	7	1	5	5	2	3
GN-70	1	1	7	7	7	7	1	7	7	3	7	1	7	5	2	7
GN-71	1	1	7	7	7	7	1	7	7	3	5	1	5	5	2	3
GN-72	1	2	7	7	7	7	1	7	7	5	7	1	3	5	3	5
GN-73	1	1	5	5	5	7	1	5	7	5	5	1	5	5	1	5
GN-74	1	2	7	7	7	7	1	7	7	5	7	1	5	5	1	7
GN-75	1	2	7	7	7	7	1	7	7	5	7	1	5	5	1	7
GN-76	1	1	7	7	7	7	1	7	7	3	7	1	7	5	1	5
GN-77	1	2	7	7	7	7	1	7	7	5	7	1	5	3	1	7
GN-78	1	1	3	7	7	7	1	7	7	5	7	1	5	5	1	3
GN-79	1	1	5	7	7	7	1	7	7	3	7	1	7	5	3	3
GN-80	1	2	7	7	7	7	1	7	5	3	5	1	5	5	3	3
GN-81	1	1	3	7	7	7	1	7	7	5	7	1	7	5	1	3
GN-82	1	1	3	5	5	1	1	5	7	5	5	1	7	5	2	3
GN-83	1	1	3	7	7	7	1	7	7	5	7	1	7	7	1	5
GN-84	1	2	7	7	7	7	1	7	7	5	7	1	5	5	1	7
GN-85	1	2	3	7	7	7	1	7	7	5	7	1	5	7	2	3
GN-86	1	1	7	7	7	7	1	7	7	5	7	1	3	5	1	7
GN-87	1	1	3	7	7	7	1	7	7	5	7	1	3	5	1	3
GN-88	1	1	7	7	7	7	1	7	7	5	7	1	5	5	1	7
GN-89	1	1	5	7	7	7	1	7	7	5	7	1	7	7	2	5
GN-90	1	3	3	7	7	7	1	7	7	5	7	1	7	7	1	3
GN-91	1	1	7	7	7	5	1	7	7	5	7	1	5	7	1	7
GN-92	1	2	3	5	5	1	1	5	7	5	5	1	5	5	1	3

GN-93	1	1	7	7	5	5	1	7	7	3	7	1	5	5	1	5
GN-94	1	2	7	7	7	7	1	7	7	5	7	1	5	5	2	3
GN-95	1	2	7	5	5	5	1	5	5	3	3	1	3	5	1	5
GN-96	1	1	5	7	7	5	1	7	7	5	5	1	5	3	1	7
GN-97	1	1	3	7	5	5	1	5	7	5	5	1	5	3	1	7
GN-98	1	2	5	7	7	7	1	7	7	5	5	1	3	5	1	7
GN-99	1	2	3	7	7	7	1	7	7	5	5	1	3	3	2	3
GN-100	1	2	7	7	7	7	1	7	7	5	7	1	3	5	2	7
GN-101	1	1	7	7	7	7	1	7	7	5	7	1	7	5	1	7
GN-102	1	2	3	7	7	7	1	7	7	5	7	1	7	5	2	5
GN-103	1	1	5	7	7	7	1	7	7	5	7	1	3	5	1	7
GN-104	1	1	3	7	7	7	1	7	5	5	5	1	7	5	2	3
GN-105	1	2	5	7	7	7	1	7	7	5	7	1	5	5	1	7
GN-106	1	1	5	7	7	5	1	5	5	5	7	1	5	3	2	7
GN-107	1	2	7	7	7	7	1	7	5	3	5	1	5	5	2	5
GN-108	1	2	3	7	7	7	1	7	7	5	5	1	7	5	2	3
GN-109	1	1	3	7	7	7	1	5	7	5	5	1	3	5	1	3
GN-110	1	1	5	7	7	7	1	7	7	5	7	1	3	5	1	5
GN-111	1	1	3	7	5	5	1	7	7	5	5	1	7	5	2	3
GN-112	1	2	5	7	7	7	1	5	7	5	7	1	7	5	3	5
GN-113	1	1	3	7	5	5	1	7	7	5	5	1	3	3	2	3
GN-114	1	2	7	7	5	5	1	5	5	5	5	1	7	5	1	7
GN-115	1	2	7	5	5	5	1	5	7	5	5	1	5	3	3	3
GN-116	1	1	7	7	7	7	1	7	7	5	7	1	7	5	3	7
GN-117	1	2	5	7	7	7	1	7	7	5	7	1	3	5	1	7
GN-118	1	2	3	7	7	5	1	7	7	3	5	1	3	3	2	5
GN-119	1	2	3	5	5	1	1	5	7	5	5	1	5	5	1	5
GN-120	1	1	3	7	7	7	1	7	7	5	7	1	5	5	3	7
GN-121	1	1	3	5	5	5	1	3	5	5	3	1	5	5	3	3
GN-122	1	1	3	7	7	7	1	7	7	5	7	1	3	3	3	5
GN-123	1	2	5	7	5	5	1	7	7	5	5	1	7	7	1	7
GN-124	1	1	3	5	5	5	1	3	3	5	3	1	5	5	3	3
GN-125	1	1	3	5	5	7	1	5	3	3	5	1	7	5	1	3
GN-126	1	2	3	7	5	5	1	7	7	5	5	1	3	5	3	3
GN-127	1	1	3	5	5	7	1	5	7	5	5	1	7	5	2	3
GN-128	1	2	3	7	7	7	1	7	5	3	7	1	3	3	3	5
GN-129	1	1	5	7	7	5	1	7	7	5	5	1	3	3	1	5
GN-130	1	2	7	7	7	7	1	7	7	5	7	1	3	5	1	5
GN-131	1	2	7	7	7	7	1	7	7	3	7	1	5	7	1	7
GN-132	1	2	3	5	5	5	1	5	7	5	5	1	7	7	2	3
GN-133	1	1	7	7	7	7	1	7	7	3	7	1	7	7	1	5
GN-134	1	1	3	7	7	7	1	7	7	5	5	1	7	7	1	3
GN-135	1	1	3	7	7	7	1	7	7	5	5	1	7	5	3	3
GN-136	1	2	7	7	5	5	1	5	5	3	7	1	5	7	2	7
GN-137	1	2	5	7	7	7	1	7	7	5	7	1	7	5	1	5
GN-138	1	1	3	7	7	7	1	7	7	5	5	1	3	7	1	3
GN-139	1	2	7	7	7	7	1	7	7	5	7	1	7	5	2	7
GN-140	1	1	3	7	7	7	1	7	7	5	7	1	7	5	2	3

GN-141	1	1	7	7	7	7	1	7	7	5	5	1	5	5	3	5
GN-142	1	2	3	7	5	5	1	7	7	5	5	1	3	3	1	5
GN-143	1	1	3	7	7	7	1	7	7	5	7	1	7	5	1	3
GN-144	1	1	7	7	7	7	1	7	7	5	7	1	7	5	1	7
GN-145	1	2	7	7	7	7	1	7	7	5	5	1	7	5	1	5
GN-146	1	1	3	7	7	7	1	7	7	5	7	1	7	5	2	5
GN-147	1	2	5	7	7	7	1	7	7	5	5	1	3	5	3	3
GN-148	1	1	3	7	7	7	1	7	7	3	5	1	7	3	1	3
GN-149	1	1	7	7	7	5	1	7	7	5	5	1	5	3	3	7
GN-150	1	1	3	5	5	7	1	5	7	5	5	1	5	3	2	3
GN-151	1	2	7	7	7	5	1	7	7	5	5	1	5	5	3	7
GN-152	1	2	3	7	7	7	1	7	7	5	5	1	5	3	2	3
GN-153	1	2	3	7	7	7	1	7	7	5	7	1	5	7	1	3
GN-154	1	2	3	5	5	5	1	5	5	5	5	1	7	7	2	5
GN-155	1	2	5	7	7	7	1	7	7	5	7	1	5	5	3	5
GN-156	1	1	5	7	7	7	1	7	7	5	7	1	5	5	1	7
GN-157	1	1	3	3	5	1	1	5	7	5	5	1	7	3	2	3
GN-158	1	1	7	7	7	7	1	5	5	3	7	1	7	5	1	3
GN-159	1	1	3	7	7	5	1	7	7	5	7	1	5	5	2	3
GN-160	1	2	3	7	7	7	1	7	7	5	5	1	5	5	1	3

ACH: Anthocyanin Coloration of Hypocotyl, SC: Shape of Cotyledon, PH: Plant Height, NBL: Number of Basal Leaves, LD: Leaf Density, GCP: Green Color of Plant, LP: Leaf Pubescence, STL: Size of Terminal Leaf, ILS: Intensity of Leaf Serration, LMS: Leaf Margin Status, PL: Petiole Length, ACF: Anthocyanin Coloration of Flower, FS: Fruit Size, IBCF: Intensity of Brown Color in Fruit, FSH: Fruit Shape, TBF: Time of Beginning of Flowering.
